# Stabilization of Single Metal Atoms on Graphitic Carbon Nitride: Synthetic Strategies and Emerging Applications

**DOI:** 10.1002/advs.202516977

**Published:** 2026-02-18

**Authors:** Wenyao Zhang, Junjie Cui, Zhenjie Yao, Boyuan Zhu, Nithinraj Panangattu Dharmarajan, Ajayan Vinu, Junwu Zhu

**Affiliations:** ^1^ Key Laboratory for Soft Chemistry and Functional Materials Ministry of Education Nanjing University of Science and Technology Nanjing 210094 China; ^2^ Global Innovative Centre for Advanced Nanomaterials College of Engineering Science and Environment The University of Newcastle Newcastle NSW 2308 Australia

**Keywords:** electrocatalysis, graphitic carbon nitride, organic chemical transformations, photocatalysis, single‐atom catalysts

## Abstract

Emerging as a new frontier in catalysis science, single‐atom catalysts (SACs) have sparked broad attention owing to their maximum atom utilization efficiency, well‐defined active sites, and unique structures and properties. The prerequisite for the scientific research and practical application of SACs is to stabilize and maintain the highly reactive isolated metal atoms on suitable supports. Graphitic carbon nitride (g‐C_3_N_4_), with characteristic 2D architecture, abundant unsaturated nitrogen coordination sites, and periodic structural cavities, serves as an exceptional substrate for stabilizing isolated SACs, while the inherent semiconductor nature further enables visible‐light‐responsive photocatalytic activity for solar energy conversion and environmental remediation. Importantly, an intensive investigation has pushed the study of SACs@g‐C_3_N_4_ far beyond what people can imagine in the beginning, but less well summarized. Herein, a panorama review of recent advances in SACs@g‐C_3_N_4_ catalysts, including i) advanced synthetic strategies for SACs@g‐C_3_N_4_ with special emphasis on the stabilization of isolated metal atoms against migration and aggregation during the synthesis processes, and ii) appealing achievements of SACs@g‐C_3_N_4_ in a variety of emerging applications, including electrocatalysis, photocatalysis, and organic chemical transformations. Finally, current challenges are highlighted and an outlook on the prospects for future development of this fascinating research hotspot is presented based on pioneering studies.

## Graphical abstract

This review highlights recent advancements in stabilizing single metal atoms on graphitic carbon nitride emphasizing innovative synthesis strategies and emerging applications in electrocatalysis, photocatalysis and organic transformations, along with key challenges and future perspective.

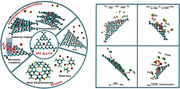

## 1 Introduction

Catalysis represents a vital chemical process of both academic and industrial interest since it is the most reasonable, economical, and environmentally benign strategy for achieving chemical transformation, energy conversion, and environmental remediation.^[^ ^1^, ^2^, ^3^, ^4^ ^]^ Over 90% of globally produced chemicals involve catalysts in at least one of their steps.^[^ ^5^, ^6^, ^7^ ^]^ Consequently, catalyst synthesis continues to hold the key to a successful chemical industry. Considerable research efforts have been devoted to developing highly active, selective, and cost‐effective catalysts, as well as to understanding their catalytic mechanisms and structure‐property relationships.^[^ ^8^, ^9^ ^]^ Additionally, the past decades have witnessed revolutionary breakthroughs in the development of nanomaterial science and the advancement of characterization techniques,^[^ ^10^, ^11^, ^12^, ^13^ ^]^ which provide an opportunity to design and fabricate catalysts at the molecular or atomic level.

In particular, single‐atom catalysts (SACs) recently emerged as a new research hotspot in the catalysis community.^[^ ^14^ ^]^ The SACs with isolated atomic metal species on solid support surfaces present the ultimate goal of bridging the gap between homogeneous and heterogeneous catalysts to deliver the combined merits of both.^[^ ^15^, ^16^, ^17^ ^]^ The development of SACs has attracted extensive interdisciplinary attention, ranging from typical thermochemical catalysis to photochemical and electrochemical conversions.^[^ ^18^, ^19^, ^20^ ^]^ Such catalysts can fully expose the metal active sites for triggering catalytic reactions and have great potential to enable the use of metal resources and maximum atom‐utilization efficiency.^[^ ^21^, ^22^ ^]^ To obtain the atomic distribution against aggregation into particles, SACs are typically anchored by the surrounding coordination species of the substrates through strong interactions or considerable charge transfer, which in turn renders the SACs with unique electronic structures that are distinct from conventional metal nanocatalysts. The fully exposed active sites and unique structural properties of SACs have been proven to result in remarkable improvements in the catalytic activity in a variety of reactions.^[^ ^23^, ^24^, ^25^ ^]^ Moreover, the uniform and spatially isolated active sites in SACs are similar to those of homogeneous catalyst analogs, which are beneficial for achieving enhanced catalytic chemoselectivity owing to their similar electronic interactions with substrates.^[^ ^26^, ^27^ ^]^ Structural simplicity can effectively inhibit the formation of detrimental intermediates produced by side reactions on metal nanocatalysts.^[^ ^28^, ^29^ ^]^ Therefore, SACs hold great promise as an ideal platform for understanding the relationship between structure and catalytic performance as well as for investigating catalytic mechanisms at the atomic or molecular level, which promotes the reasonable design of targeted catalysts for desired catalytic performances.^[^ ^21^, ^23^ ^]^

The prerequisite for scientific research and practical application of SACs is to effectively stabilize single metal atoms on suitable supports. Carbon nitride is an appealing class of nanomaterials that can complement carbon in a variety of applications,^[^ ^30^ ^]^ thereby numerous structures of carbon nitride have been constructed since the first example in the family reported by Berzelius and Liebig in 1834.^[^ ^31^, ^32^ ^]^ Among these allotropes, graphitic carbon nitride (g‐C_3_N_4_) was identified as the most stable under ambient conditions.^[^ ^33^, ^34^ ^]^ The g‐C_3_N_4_ possesses a typical 2D structure stacked through van der Waals forces, and each layer consists of *tri‐s*‐triazine subunits coupled via planar tertiary amino groups, resulting in periodic structural N‐coordinating cavities in the lattice.^[^ ^33^, ^35^, ^36^, ^37^ ^]^ The high level of pyridinic nitrogen in each cavity is capable of firmly capturing metal ions, benefiting from the nature of the abundant electron lone pairs. Owing to its high nitrogen content, g‐C_3_N_4_ is considered an excellent substrate for stabilizing isolated SACs, particularly those with high mass loading. On the other hand, as a fascinating metal‐free semiconductor, g‐C_3_N_4_ has a moderate bandgap of ca. 2.7 eV and exhibits great potential in fields related to solar energy conversion and environmental remediation.^[^ ^38^, ^39^, ^40^, ^41^, ^42^ ^]^

Specifically, harnessing the unique metal‐free architecture and an inherently visible‐light‐active electronic structure, g‐C_3_N_4_ emerges as a uniquely versatile material platform that transcends the limitations of conventional metal‐based semiconductors (e.g., metal oxides) and high‐cost 2D materials (e.g., MXenes, TMDs). In photocatalysis, it drives a suite of solar‐fuel and environmental reactions—including hydrogen evolution, CO_2_ reduction, pollutant degradation, and bacterial disinfection—with high photon efficiency and operational stability seldom achieved by its metallic counterparts. For energy storage, while its intrinsic conductivity is lower than that of MXenes,^[^ ^43^, ^44^ ^]^ the abundant pyrrolic and pyridinic nitrogen sites within its matrix act as powerful anchoring centers for charge storage, enabling the design of high‐performance supercapacitor electrodes. Furthermore, its rich surface functionality and tunable band structure facilitate the seamless construction of type‐II or Z‐scheme heterojunctions with metal oxides (e.g., TiO_2_, ZnO) and TMDs,^[^ ^45^ ^]^ which not only boost charge separation kinetics beyond the capabilities of the individual components but also unlock advanced sensing and catalytic functionalities. This synergistic multifunctionality, coupled with the material's low cost, scalable synthesis, and biocompatibility, positions g‐C_3_N_4_ as a distinctly sustainable and economically viable alternative in the broader landscape of functional materials. Owing to its extremely inexpensive and abundant raw materials, simple and scalable synthesis, combined with its high physicochemical stability, tunable optical properties, and appealing electronic structure, g‐C_3_N_4_ is expected to be one of the most promising candidates for next‐generation photocatalysts, which is considered to have great potential to provide a promising solution strategy for future energy issues.^[^ ^46^, ^47^, ^48^, ^49^, ^50^ ^]^

Importantly, the fundamental chemical and catalytic properties of g‐C_3_N_4_ depend significantly on its original molecular structure. Enormous efforts have been devoted to engineering the molecular structure of g‐C_3_N_4_, including nanostructuring,^[^ ^38^, ^51^, ^52^, ^53^, ^54^ ^]^ supramolecular preorganization,^[^ ^55^, ^56^, ^57^, ^58^ ^]^ copolymerization of monomers,^[^ ^59^, ^60^, ^61^ ^]^ delamination to a few‐layer thickness,^[^ ^51^, ^62^, ^63^, ^64^, ^65^ ^]^ and heteroatom doping.^[^ ^66^, ^67^, ^68^, ^69^, ^70^, ^71^, ^72^, ^73^, ^74^ ^]^ Among them, doping by the incorporation of metal ions or single metal atoms is an effective approach to introduce active sites into the g‐C_3_N_4_ framework to modify the electronic structures and energy band configurations, further improving the catalytic performance.

**Figure 1:**
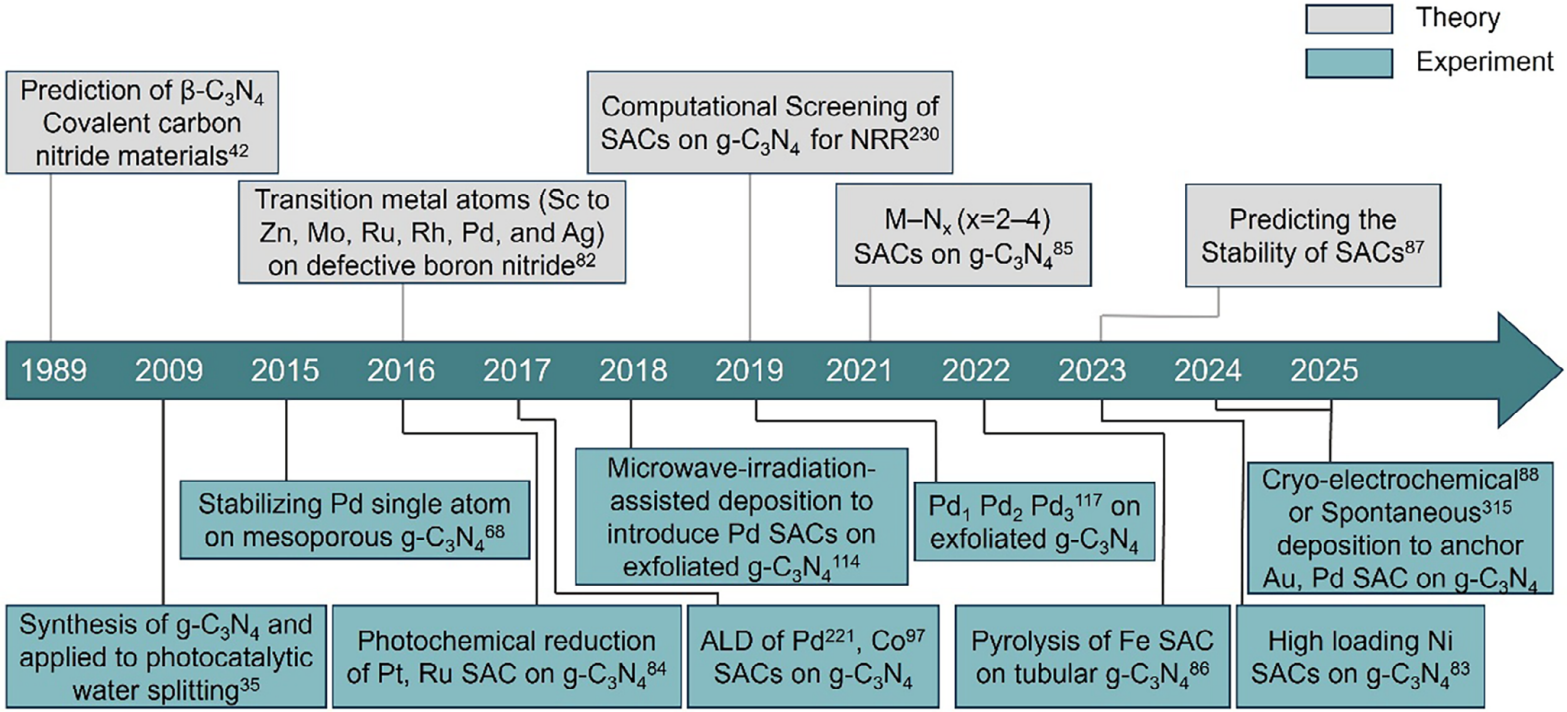
A timeline of theoretical and experimental advances in SACs@g‐C_3_N_4_. Over the last two decades, the elucidation of theoretical predictions has gradually deepened, and much more advanced methods have been developed to take advantage of SACs and g‐C_3_N_4_.

However, few published reviews have summarized the literature from the perspective of synthesis strategies and emerging applications for stabilizing SACs on g‐C_3_N_4_ (SACs@g‐C_3_N_4_). Since the first report from Javier Perez‐Ramírez, Zupeng Chen, and their colleagues in 2015 on [Pd]mpg‐C_3_N_4_ for the three‐phase hydrogenation of alkynes and nitroarenes,^[^ ^68^ ^]^ the study of SACs@g‐C_3_N_4_ has rapidly grown into one of the new frontiers in materials science and has recently inspired intensive research enthusiasm.^[^ ^10^, ^75^, ^76^ ^]^ In the SACs aspect, g‐C_3_N_4_, as a support, can not only achieve precise control of the coordination environment and geometric configurations via abundant and uniform periodic metal coordination sites but also, more importantly, provide more precise information for the identification of catalytically active sites, which is an intractable challenge for SACs.^[^ ^77^, ^78^, ^79^, ^80^, ^81^, ^82^ ^]^ Consequently, even if g‐C_3_N_4_ itself is inert to several catalytic systems, such as electrochemical reactions, there are numerous outstanding reports regarding SACs@g‐C_3_N_4_ for emerging electrocatalytic processes.^[^ ^83^ ^]^ From the g‐C_3_N_4_ aspect, the incorporation of SACs can effectively optimize the electronic structures of g‐C_3_N_4_ and accelerate the interfacial reaction dynamics, which is crucial for strengthening the activity and providing atomic‐level insights into the intrinsic active sites and catalytic pathways.^[^ ^84^ ^]^ That is, the combination of SACs and g‐C_3_N_4_ can work in concert to express the specific characteristics of the individual components, and remarkably broaden their applications outside of conventional catalysis.^[^ ^85^, ^86^, ^87^, ^88^ ^]^ The timeline of major breakthroughs in SACs@g‐C_3_N_4_ during the past decades is summarized in **Figure** **1**. The boundaries of this area have possibly been pushed far beyond what people initially imagined.

**Figure 2:**
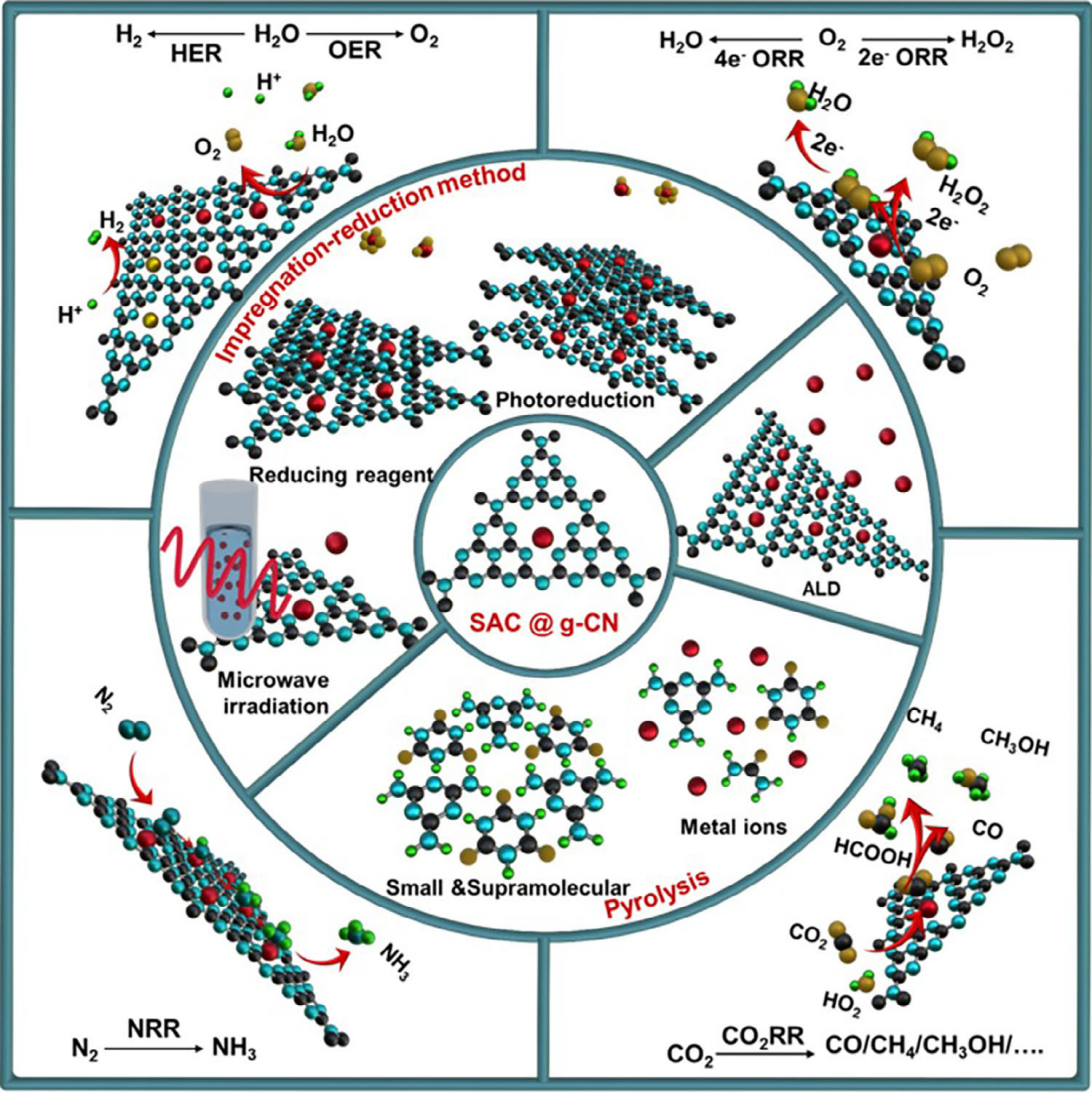
Overview of the topics covered in this review.

In this review, we summarize recent advances in synthetic strategies for SACs@g‐C_3_N_4_ with special emphasis on how to stabilize isolated metal single atoms against migration and aggregation during the synthesis processes. Furthermore, we highlight the achievements of SACs@g‐C_3_N_4_ in a variety of emerging applications, including electrocatalysis, photocatalysis, and organic chemical transformations (**Figure** **2**). The introduction of the applications starts by discussing the roles of SACs and g‐C_3_N_4_ in a certain reaction under theoretical and experimental investigations, then delineating the correlation between the structural features and specific catalytic properties of SACs@g‐C_3_N_4_, and finally expanding the tuning or influence of the catalytic behaviors by tailoring the electronic interactions between the isolated metal atoms and the g‐C_3_N_4_ support. Finally, based on the pioneering studies, we present an outlook on the prospects and challenges for future research in this fascinating research hotspot.

## 2 Synthetic Methods for SACs@g‐C_3_N_4_

The exploration and development of synthetic methodologies for SACs@g‐C_3_N_4_ have attracted considerable attention. However, owing to their high surface energies, single metal atoms are often susceptible to migration and agglomeration into clusters or particles.^[^ ^89^, ^90^, ^91^ ^]^ Therefore, the stabilization of SACs and the realization of uniform atomic dispersion of metal species under realistic synthesis and reaction conditions remain challenging.^[^ ^92^ ^]^ The central goal of this study lies in the formation of strong interactions (bonds) between the metal atoms and the supports. To date, a range of preparation methods have been developed, and on the basis of the integration mode of the components, these methods can be summarized into three categories: atomic layer deposition (ALD), impregnation‐reduction method, and pyrolysis (**Table** **1**). In this section, we systematically discuss the recent progress in the synthesis of SACs@g‐C_3_N_4_ materials according to the above three categories.

**Table 1:** Representative SACs@g‐C_3_N_4_ synthesized via various methods.

Method	Samples	Precursors	Reaction conditions	Refs.
ALD	Co_1_/PCN	bis(cyclopentadienyl)cobalt, phosphorus‐doped g‐C_3_N_4_	Co ALD, 150 °C, 225 s O_3_ treatment, 150 s	[97]
	Pd_1_/C_3_N_4_	palladium hexafluoroacetylacetate, bulk g‐C_3_N_4_	Pd ALD, 150 °C, 120 s Ultrahigh purity N_2_ protection	[220]
Impregnation‐reduction strategy	Pt‐CN	H_2_PtCl_6_, bulk g‐C_3_N_4_	125 °C, 1 h, Ar atmosphere	[84]
	PtSA‐CN	H_2_PtCl_6_, N‐vacancy‐riched g‐C_3_N_4_	ice‐assistant photodeposition, 300 W Xe lamp, 3 min	[127]
	Pt_x_‐CN	H_2_PtCl_6_, g‐C_3_N_4_	Photodeposition, visible light irradiated (λ > 420 nm) for 4 h	[159]
	[Pd]mpg‐C_3_N_4_	PdCl_2_, Mesoporous g‐C_3_N_4_	NaBH_4_ reduction	[68]
	saNi‐nC_3_N_4_	Ni(NO_3_)_2_·6H_2_O, dicyanamide synthesized g‐C_3_N_4_	NaBH_4_ reduction	[111]
	Cu@mpgC_3_N_4_	CuCl_2_ 6H_2_O, NH_2_CN g‐C_3_N_4_	NaBH_4_ reduction, microwave irradiation	[112]
	Pd‐BCN‐BA‐0.9	Pd‐(NH_3_)_4_(NO_3_)_2_, Mesoporous C‐doped g‐C_3_N_4_	Microwave‐assisted deposition, 100 W, 5 min	[113]
	Pd‐ECN	Pd‐(NH_3_)_4_(NO_3_)_2_, Mesoporous g‐C_3_N_4_	Microwave‐assisted deposition, 100 W, 5 min	[114]
	ECN‐Pd	Pd‐(NH_3_)_4_(NO_3_)_2_, Exfoliated g‐C_3_N_4_	Microwave‐assisted deposition, 100 W, 5 min	[116]
	Co@g‐C_3_N_4_	CoCl_2_ 6H_2_O, g‐C_3_N_4_	Microwave irradiation	[119, 120]
	M/g‐CN (M = Pt, Pd)	PdCl_2_, H_2_PtCl_6_, g‐C_3_N_4_ nanosheets	Photodeposition, 350 W Xe lamp, 30–60 min	[157, 162]
	RuSA‐mC_3_N_4_	RuCl_3_, Mesoporous g‐C_3_N_4_	Microwave, 2 min, 18‐20 run	[118]
	Ru‐C_3_N_4_	RuCl_3_, Exfoliated g‐C_3_N_4_	Reflux, 4 h	[133, 134, 135]
	Ru_1_/mpg‑C_3_N_4_	RuCl_3_, Mesoporous g‐C_3_N_4_	300 °C, 2 h, N_2_ atmosphere	[141]
	PtCu‐crCN	H_2_PtCl_6_, CuCl_2_, crystalline g‐C_3_N_4_	Photodeposition, 300 W Xe lamp, 10 min	[168]
	PtRuSA‐CN	H_2_PtCl_6_, RuCl_3_, N‐vacancy‐rich g‐C_3_N_4_	Photodeposition, 300 W Xe lamp, 10 min	[167]
	Au_1_/mpg‐C_3_N_4_	HAuCl_4_, Mesoporous g‐C_3_N_4_	300 °C, 2 h, Ar/H_2_ atmosphere	[140]
	Au_1_N*_x_* single‐site/g‐C_3_N_4_	HAuCl_4_, ammonia‐treatment g‐C_3_N_4_	Room temperature stirring, 8 h	[130]
	M‐ECN‐P (M = Pt, Ir, Pd)	H_2_PtCl_6_ or K_2_IrCl_6_·6H_2_O or PdCl_2_, Exfoliated g‐C_3_N_4_	NaBH_4_ reduction	[108]
	Co_1_P_4_@g‐C_3_N_4_	Co(NO_3_)_2_, g‐C_3_N_4_	300 °C, 2 h, Ar atmosphere with NaH_2_PO_2_·H_2_O	[126]
	g‐C_3_N_4_/CoPc‐COOH	Cobalt(II) phthalocyanine, g‐C_3_N_4_	Stirring absorption, 48 h	[170]
	Fe_2_/mpg‐C_3_N_4_	Fe_2_O_4_C_14_H_10_, Mesoporous g‐C_3_N_4_	300 °C, 2 h, Ar/H_2_ atmosphere	[142]
	FePc/CN	Iron(II) phthalocyanine, g‐C_3_N_4_	ice‐assisted stirring, 3 h	[169]
	g‐C_3_N_4_/ZnTcPc	Zinc tetracarboxyphthalocyanine, g‐C_3_N_4_	Ultrasonication, 5 h	[172, 173]
	(M_1_/CN, M= Ag, Cu, Ni, Co, V, Fe, Zn, Ru, Ce, Sm, Mo, W, Bi	Metal target, g‐C_3_N_4_	Electrochemical deposition, 50 V	[174]
	Cu SAs/CN	Copper target, g‐C_3_N_4_	Electrochemical deposition, 30 V	[175]
Pyrolysis	M‐MCN‐D (M= Pt, Ir, Au, Pd, and Ag)	H_2_PtCl_6_·xH_2_O, K_2_IrCl_6_·6 H_2_O,PdCl_2_, K_2_Pt(CN)_4_·xH_2_O, KAu(CN)_2_, K_2_Pd(CN)_4_·xH_2_O, Ag(C_4_N_3_), cyanamide	550 °C, 4 h, N_2_ atmosphere	[108]
	g‐C_3_N_4_‐Pt^2+^	H_2_PtCl_6_, dicyandiamide	425 °C, 4 h, Ar atmosphere	[109]
	Co‐C_3_N_4_	CoCl_2_·6H_2_O, dicyandiamide or melamine	500‐600 °C, N_2_ atmosphere	[69, 186, 208]
	Co‐g‐C_3_N_4_	Cobalt salen complex, melamine	600 °C, Ar atmosphere	[214]
	Co_1_/C_3_N_4_	Co(OAc)_2_, dicyandiamide	550 °C, 4 h, N_2_ atmosphere	[185]
	Ni‐CN_x_	NiCl_2_, cyanuric acid, 2,4‐diamino‐6‐phenyl‐1,3,5‐triazine	550 °C, 4 h, N_2_ atmosphere	[205]
	Ni‐FCN	NiCl_2_, dicyandiamide	520 °C, 2 h, N_2_ atmosphere, H_2_SO_4_ wash	[191]
	Fe‐g‐C_3_N_4_	FeCl_2_, dicyandiamide	600 °C, 4 h, N_2_ atmosphere	[183]
	Fe@g‐C_3_N_4_	FeCl_2_ 4H_2_O, dicyandiamidine nitrate	550 °C, 4 h, N_2_ atmosphere	[209]
	FeN_x_/g‐C_3_N_4_	FeSO_4_·7H_2_O, 2‐methylimidazole, PVP, melamine	600 °C, 5 h, N_2_ atmosphere	[176]
	Mn@g‐C_3_N_4_	Mn(acac)_2_, urea	550 °C, 22 h, N_2_ atmosphere	[218]
	Cu‐C_3_N_4_	CuCl_2_, dicyandiamide	600 °C, N_2_ atmosphere, HNO_3_ wash	[66]
	Cu/PCN	CuCl_2_, urea	550 °C, 4 h,	[187]
	g‐C_3_N_4_‐Mn	KMnO_4_, urea	550 °C, 4 h, N_2_ atmosphere	[190]
	AgTCM‐mpg‐CN	Silver tricyanomethanide, cyanamide	550 °C, 4 h, N_2_ atmosphere	[70]
	SDAg‐CQDs/UCN	Silver tricyanomethanide, dicyandiamide	550 °C, 3 h, N_2_ atmosphere	[210]
	AgTCM/UCN	Silver tricyanomethanide, dicyandiamide	500 °C, 3 h	[211]
	Ag/mpg‐C_3_N_4_	Silver tricyanomethanide, cyanamide	550 °C, N_2_ atmosphere	[212]
	Ru‐Ala‐C_3_N_4_	RuCl_3_, melamine, L‐alanine	550 °C, 2 h, N_2_ atmosphere	[194]
	La‐CN	La_2_(CO_3_)_3_ 8H_2_O, Urea	550 °C, 2 h, N_2_ atmosphere	[195]
	W‐CN	Ammonium metatungstate hydrate, dicyandiamide	515 °C, 3 h, N_2_ atmosphere	[219]

### 2.1 Atomic Layer Deposition

Atomic layer deposition (ALD) is a powerful technique for the preparation of supported metal clusters or SACs because of its ability to precisely control the size and regulate the surface structure through self‐limiting binary reactions between gaseous precursors and the substrate.^[^ ^93^, ^94^, ^95^, ^96^ ^]^ Cao et al.^[^ ^97^ ^]^ devoted much effort to the synthesis of SACs on g‐C_3_N_4_ using the ALD method. To obtain a well‐defined, uniform, single metal cobalt catalyst firmly anchored on g‐C_3_N_4,_ a bis(cyclopentadienyl)cobalt was used as the metal source, followed by O_3_ treatment to remove the surface ligand. Experimental assessments revealed unexpected activity, and outstanding stability was ascribed to the strong coordinate attachment of Co atoms to g‐C_3_N_4_ with the Co_1_–N_4_ geometry. The main matrix network and morphology of g‐C_3_N_4_ showed nearly no influence after the Co ALD process. Lu et al.^[^ ^98^ ^]^ also constructed single Cu atoms on g‐C_3_N_4_ (Cu_1_/g‐C_3_N_4_)using the ALD method. Then, a dual‐atom catalyst, Ni_y_Cu_1_/g‐C_3_N_4_ catalysts (y is the atomic ratio of Ni to Cu) were fabricated through depositing Ni species on the pre‐prepared Cu_1_/g‐C_3_N_4_ using NiO_x_ ALD technology, which was determined as high metal loading of Cu (8.1 wt.%) and Ni (3.1 wt.%). However, the main limitations of ALD for synthesizing SACs@g‐C_3_N_4_ are their high cost and low yield, which fundamentally hinder their industrial adoption. The process requires expensive, high‐purity precursors and sophisticated vacuum equipment, while its layer‐by‐layer nature is inherently slow and not scalable for mass production.

### 2.2 Impregnation‐Reduction Method

As one of the most common methods for preparing metal SACs, the impregnation‐reduction approach has distinct advantages, such as simple operation, no requirement for additional complicated steps, and specialized equipment.^[^ ^99^, ^100^ ^]^ A typical impregnation‐reduction approach involves three processes: i) introduction of metal precursors on support materials by impregnation absorption under appropriate conditions, ii) drying, and iii) suitable reduction procedures to strengthen the interactions between SACs and the support. During these processes, controlling the content and appropriate reduction and activation steps are of great importance for the preparation of SACs. First, the target metal species in the isolated form are always anchored on the supports by either physical absorption or chemical coordination. Then, depending on the nuclear pattern (mononuclear or multinuclear), this mixture can be reduced to obtain SACs, metal clusters, or nanoparticles.

**Figure 3:**
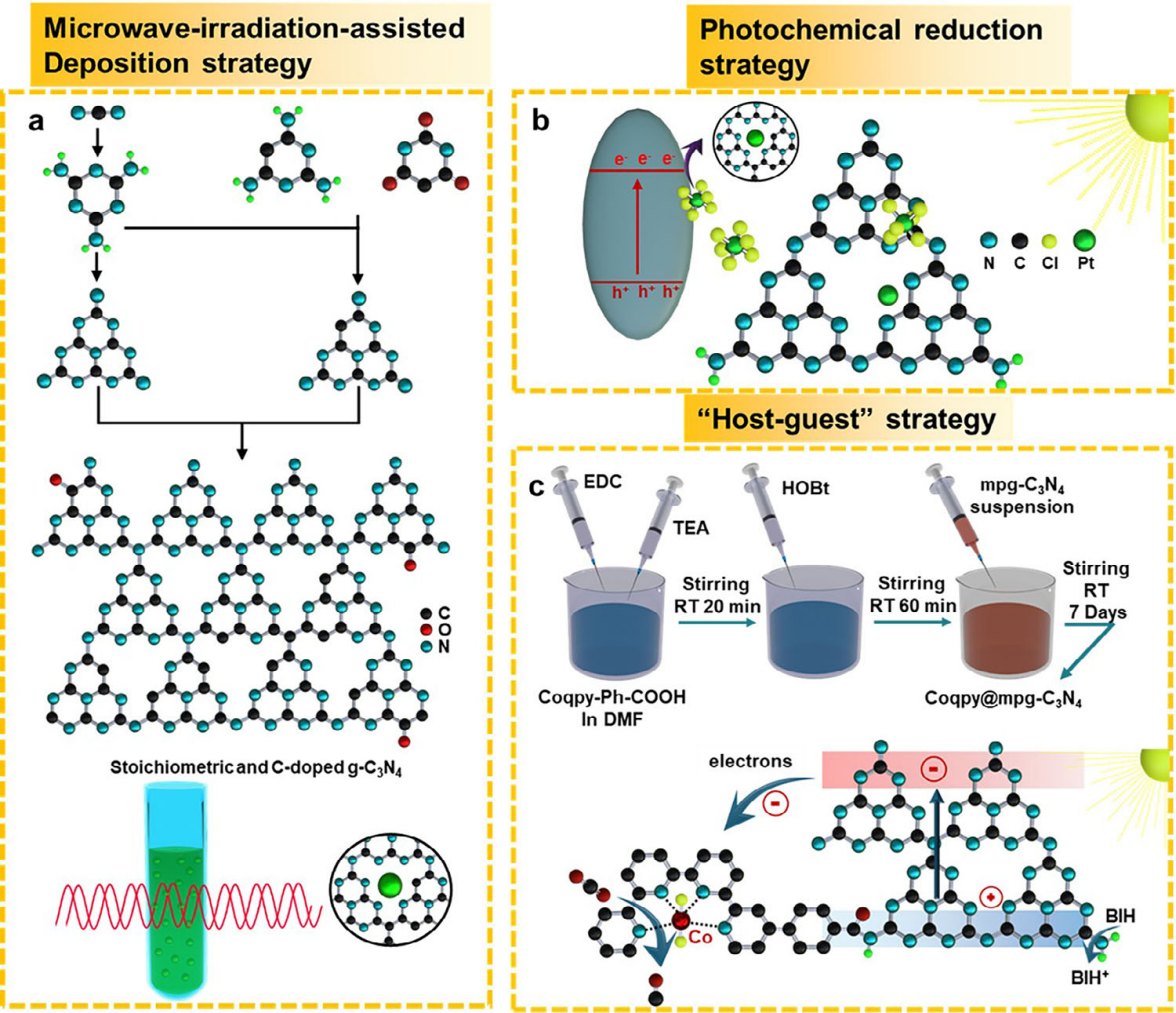
Representative examples of metal SACs supported on g‐C_3_N_4_ through an impregnation‐reduction strategy. a) Schematic illustration of the synthesis procedure for C‐doped g‐C_3_N_4_ and subsequent introduction of palladium by microwave irradiation‐assisted metal deposition. Adapted with permission.^[^ ^113^ ^]^ Copyright 2017, RSC. b) Illustration of the synthesis for Pt single atoms anchored on g‐C_3_N_4_ nanosheets by photoexcited electrons via the photodeposition process. Adapted with permission.^[^ ^158^ ^]^ Copyright 2018, American Chemical Society. c) Preparation of the coqpy@mpg‐C_3_N_4_ hybrid assembly and illustration of the visible‐light driven CO_2_ to CO reduction process, Adapted with permission.^[^ ^171^ ^]^ Copyright 2020, American Chemical Society.

Owing to the high surface energy of isolated metal atoms, their migration and aggregation into clusters or particles is another significant obstacle in the preparation process. Thus, the strong interactions between the metal precursors and the supports are considered to be key factors that guarantee the atomic dispersion of isolated metal single atoms and hinder the agglomeration of atoms. g‐C_3_N_4_, as an ideal carbon‐based support, offers the promise of efficiently and effectively stabilizing SACs owing to its high level of pyridine‐like nitrogen in its intrinsic structure, which could provide abundant and uniform electron lone pairs to capture metal species.^[^ ^25^, ^101^, ^102^ ^]^ Unlike metal–nitrogen/carbon (M–N_x_/C) coordination materials, g‐C_3_N_4_ can homogeneously coordinate with metal species in the impregnation stage and firmly anchor with isolated metal atoms to prevent aggregation during reduction. The impregnation‐reduction strategy for obtaining SACs@g‐C_3_N_4_ can be divided into five classes:

1. Reducing reagent route: The chemical‐reducing reagent route is a straightforward and powerful approach for loading metal clusters or particles onto support materials.^[^ ^103^, ^104^, ^105^, ^106^, ^107^ ^]^ This route can also be used to load SACs on g‐C_3_N_4_ under the premise of controlling the metal content and concentration of the reducing reagent. For example, Gianvito Vile et al.^[^ ^68^ ^]^ synthesized SACs@g‐C_3_N_4_ (labeled [Pd]mpg‐C_3_N_4_) by impregnation of an aqueous solution of palladium(II) chloride and sodium chloride with mesoporous g‐C_3_N_4_ under ultrasonication and reduction with sodium borohydride. The resulting [Pd]mpg‐C_3_N_4_ composite contains 0.5 wt.% Pd, and the isolated single Pd atoms can be directly confirmed by Aberration‐Corrected Scanning Transmission Electron Microscopy (AC‐STEM). They, for the first time, demonstrate that the “sixfold cavities” in the structure of g‐C_3_N_4_ can act as “cages”, thus providing high‐density and homogeneously distributed anchoring sites for trapping isolated Pd atoms. In comparison with the Pd deposition behavior on other supports, such as alumina, even though alumina possesses much more accessible sites, a rapid aggregation phenomenon of Pd can be observed, suffering from the nature of their weak interactions. On the other hand, this confirms that g‐C_3_N_4_ provides not only a strong interaction with the isolated Pd atoms but also a large number of uniformly anchored sites, which play critical roles in the stabilization of Pd SACs. In a similar fashion, Chen et al.,^[^ ^108^ ^]^ Li et al.,^[^ ^109^ ^]^ Mondelli et al.,^[^ ^110^ ^]^ Gianvito Vilé et al.,^[^ ^111^, ^112^ ^]^ also demonstrated the synthesis of single‐atom platinum,^[^ ^108^, ^109^ ^]^ palladium,^[^ ^108^, ^110^ ^]^ gold,^[^ ^110^ ^]^ iridium,^[^ ^108^ ^]^ ruthenium,^[^ ^110^ ^]^ nickel,^[^ ^111^ ^]^ copper^[^ ^112^ ^]^ on g‐C_3_N_4_ with a relatively low metal loading to achieve atomic dispersion of metal species.
2. Microwave‐irradiation‐assisted deposition route: As illustrated in **Figure** **3**a,J. Perez‐Ramirez and co‐workers^[^ ^113^, ^114^, ^115^, ^116^, ^117^ ^]^ developed a microwave‐irradiation‐assisted deposition approach to effectively introduce SACs on g‐C_3_N_4_. As a typical example, the Pd SACs@g‐C_3_N_4_ (denoted as Pd‐ECN) was prepared by impregnating a Pd(NH_3_)_4_(NO_3_)_2_ solution with exfoliated g‐C_3_N_4_, which was subsequently reduced by microwave irradiation.^[^ ^114^ ^]^ High‐Angle Annular Dark‐Field STEM (HAADF‐STEM) analysis of the atomic positions of the Pd‐ECN sample showed a good match with the calculated Rayleigh distributions and the measured nearest‐neighbor distances, confirming a random distribution of single Pd atoms over g‐C_3_N_4_. X‐ray Absorption Spectroscopy (XAS) demonstrate the coordination environment of Pd to nitrogen, and possibly to carbon, in Pd‐ECN, and importantly, no Pd–Pd bonds can be detected, indicating a high single‐atom loading efficiency with the metal contents close to 0.5 wt.% via this microwave‐irradiation‐assisted deposition approach. Further efforts were made to increase the metal content and loading efficiency using this approach. Other g‐C_3_N_4_ scaffolds, including linear melem oligomers and poly(triazine/heptazine imides), were also considered to stabilize isolated palladium atoms,^[^ ^115^ ^]^ which revealed that the metal‐support interaction can be effectively manipulated depending on the framework structure of g‐C_3_N_4_. Enhanced interaction between metal atoms and the g‐C_3_N_4_‐based support is usually detected in the g‐C_3_N_4_‐based structure with a larger cavity size and the presence of chloride ions in polyimide, which is beneficial for increasing the metal loading efficiency. X‐ray photoelectron spectroscopy (XPS) analyses and Density Function Theory (DFT) calculations prove that the variation in the microstructure of g‐C_3_N_4_ leads to a change in the oxidation state of the metal and the catalytic activity of the material, ascribed to the different charge‐transfer mechanisms between the anchoring sites and the isolated metal atoms. Rational tuning of conditions such as microwave power, reaction media, and a series of SAC@g‐C_3_N_4_ have been reported, including Ru@g‐C_3_N_4_^[^ ^118^ ^]^ and Co@g‐C_3_N_4_.^[^ ^119^, ^120^ ^]^
3. Defect engineering route: Controlled construction of deficient sites on supports is an effective strategy to hinder the migration of metal atoms and hence prepare SACs.^[^ ^24^, ^89^, ^121^, ^122^, ^123^, ^124^, ^125^ ^]^ A recent study indicated that optimizing the microstructure of g‐C_3_N_4_ via doping with heteroatoms^[^ ^113^, ^116^, ^126^ ^]^ or direct integration with vacancies^[^ ^127^, ^128^, ^129^ ^]^ is beneficial for introducing structural defects. These defect sites within g‐C_3_N_4_ create electron‐deficient carbon centers and lead to additional unsaturated coordination anchoring sites, which could form strong M–C/N bonds with metal atoms, thereby modifying the surrounding electronic structures to optimize intermediate adsorption. This effect significantly enhances the metal content by effectively capturing metal precursors and anchoring metal atoms during post‐treatment. As a class of typical defects, nitrogen vacancies have been exploited in the structure of g‐C_3_N_4_ for the stabilization of SACs,^[^ ^127^ ^]^ in which a high Pt loading of up to 2.3 wt.% can be achieved. Due to the electron deficiency in the defect sites, the Pt precursors can be selectively reduced at the nitrogen vacancies, restricting the leaching and aggregation of isolated Pt atoms. Engineering the structure of the g‐C_3_N_4_ support may activate the bonding abilities of their structural nitrogen atoms and lead to a moderate reductive nature for the metal precursors, Au,^[^ ^130^ ^]^ Pd,^[^ ^131^ ^]^ Fe,^[^ ^132^ ^]^ Ru^[^ ^133^, ^134^, ^135^ ^]^ isolated atoms were successfully anchored on the surface of functionalized g‐C_3_N_4_ without adding other reductive reagents. Optimizing the morphology and surface area of the g‐C_3_N_4_ support can greatly enhance the proportion of accessible surface “cages” for metal precursor trapping, thus strengthening interactions with metal species. Even during calcination reduction, homogeneous atomic dispersion of metal species can be accomplished against sintering, owing to the abundant anchoring sites for effective stabilization of isolated atoms of Pt,^[^ ^84^, ^136^, ^137^, ^138^ ^]^ Au,^[^ ^139^, ^140^ ^]^ Pd,^[^ ^128^ ^]^ Ru,^[^ ^141^ ^]^ Fe,^[^ ^142^ ^]^ Zn,^[^ ^143^ ^]^ Sb.^[^ ^129^ ^]^ Moreover, excessive vacancy generation disrupts the structural integrity of the heptazine framework, leading to irregular coordination environments and reduced mechanical stability that may facilitate metal aggregation or leaching under operational conditions. Thus, while moderate defect engineering enhances SAC stability and activity, an over‐engineered vacancy concentration ultimately compromises the durability of the catalytic system.
4. Photochemical reduction route: A photochemical reduction strategy was used to synthesize precious metal nanocrystals on the photoresponse substrate.^[^ ^144^, ^145^, ^146^, ^147^ ^]^ Since g‐C_3_N_4_ is a typical carbon‐based semiconductor with a bandgap of ≈2.7 eV,^[^ ^35^, ^39^, ^46^, ^56^, ^148^, ^149^, ^150^, ^151^, ^152^, ^153^, ^154^ ^]^ the exciton can split upon irradiation at an appropriate wavelength, and the photogenerated electrons are expected to reduce the nearby coordinated metal species, as illustrated in Figure 3b. The adsorption of photons and electronically excited states is two critical steps in the photochemical reduction process.^[^ ^155^, ^156^ ^]^ To ensure the uniform atomic dispersion of the metal species in the solution, Zhou et al.^[^ ^127^ ^]^ developed an ice‐assisted phytochemical reduction strategy for the synthesis of high‐density Pt single atoms on g‐C_3_N_4_. The mixture of g‐C_3_N_4_ substrate and the Pt sources was first frozen with liquid nitrogen and then irradiated with a 300 W Xe lamp to achieve atomic Pt materials. For comparison, the same procedure was conducted without freezing. However, Pt nanoparticles were clearly observed after irradiation, even though the Pt precursors were atomically dispersed in the solution. This suggests that the ice‐assisted procedure plays a key role in the synthesis of Pt single‐atom materials by restricting the diffusion of photogenerated electrons into the ice layer, thus preventing the reduction and aggregation of non‐adsorbed Pt precursors. Cao et al.^[^ ^157^ ^]^ reported a similar photochemical reduction method for the fabrication of atomically dispersed Pd on g‐C_3_N_4_. The isolated Pd atoms were linked both in the bridged sites of adjacent layers and the surface sites of g‐C_3_N_4_, which offered directional charge‐transfer channels and target active sites for photocatalytic reactions. The introduction of SACs not only optimizes the electronic and geometric structures of g‐C_3_N_4_ but also acts as co‐catalysts, and constructs a built‐in electric field within this structure to boost the corresponding photocatalytic reactions.^[^ ^126^, ^158^, ^159^ ^]^ The photochemical‐assisted deposition successfully stabilized Pt,^[^ ^160^, ^161^, ^162^ ^]^ Pd,^[^ ^163^, ^164^ ^]^ Ru,^[^ ^165^ ^]^ Ag^[^ ^166^ ^]^ single atoms, and was further applied to synthesize Pt–Ru^[^ ^167^ ^]^ and Pt–Cu^[^ ^168^ ^]^ dual‐atom catalysts (DACs).
5. Other synthetic routes: In addition to the aforementioned reduction approaches, some other clever methods have been developed for the stabilization of SACs on g‐C_3_N_4_. Considering the intrinsically π‐conjugated systems in g‐C_3_N_4_, several recent studies have reported the combination of various types of g‐C_3_N_4_ with metal complexes, such as Fe,^[^ ^142^, ^169^ ^]^ Co,^[^ ^170^, ^171^ ^]^ and Zn.^[^ ^172^, ^173^ ^]^ This “host‐guest” construction derives from the intimate connection of two‐dimensional domains with different electron affinities, thus forming an in‐plane π‐conjugated single‐atom heterostructure system. Ma et al.^[^ ^171^ ^]^ demonstrated an amide linkage route to chemically integrate a Co‐quaterpyridine molecular complex with mesoporous g‐C_3_N_4_. As illustrated in Figure 3c, covalent grafting occurs with the assistance of 1‐ethyl‐3‐(3‐(dimethylaminopropyl)carbodiimide, triethylamine, and 1‐hydroxybenzotriazole in the DMF reaction medium. This relatively complicated reaction procedure ensured the realization of the designed covalent link between the two moieties. The covalent attachment of the molecular catalyst to mesoporous g‐C_3_N_4_ is key for improving the electron transfer dynamics, thus allowing for better control of the catalyst structure and better selectivity and activity. Yan et al.^[^ ^174^ ^]^ reported an electrochemical method for anchoring a wide range of SACs covering Ag, Cu, Ni, Co, V, Fe, Zn, Ru, Ce, Sm, Mo, W, and Bi on g‐C_3_N_4_, which was performed in constant potential mode with a voltage of 50 V. Chen et al.^[^ ^175^ ^]^ reported an ingenious electrochemical deposition method for synthesizing Cu SACs nanozymes on g‐C_3_N_4_.

The synthesis of SACs@g‐C_3_N_4_ through defect engineering, photochemical reduction, and microwave‐assisted deposition demonstrates fundamentally distinct mechanistic pathways in establishing metal‐support interactions: defect engineering creates nitrogen vacancies as electron‐deficient anchoring sites for covalent M–N/C bonding; photochemical reduction employs photoexcited electrons to reduce and stabilize metal atoms via charge transfer; while microwave deposition utilizes rapid thermal energy to simultaneously generate defects and incorporate metal species. Despite the versatility of these approaches, conventional impregnation‐reduction strategies face significant limitations in scalability and control, primarily due to the difficulty in achieving uniform metal reduction and suppressing nanoparticle formation without compromising the structural integrity of g‐C_3_N_4_, ultimately hindering their reproducibility for industrial applications.

### 2.3 Pyrolysis

The pyrolysis approach is currently considered to be the simplest, most promising, and successful method for stabilizing SACs on g‐C_3_N_4_, which is achieved by thermal copolymerization of the precursors, including metal sources and g‐C_3_N_4_ precursors, at elevated temperatures under a controlled atmosphere (i.e., Ar, H_2_, N_2_, or NH_3_). There are versatile precursors to choose for the stabilization of metal SACs and nanostructure engineering, which are crucial in determining their activities for certain applications. In general, the precursors come from three different classes: hybrids of metal ions and simplex nitrogen‐rich precursors, supramolecular precursors, and small molecular precursors.

**Figure 4:**
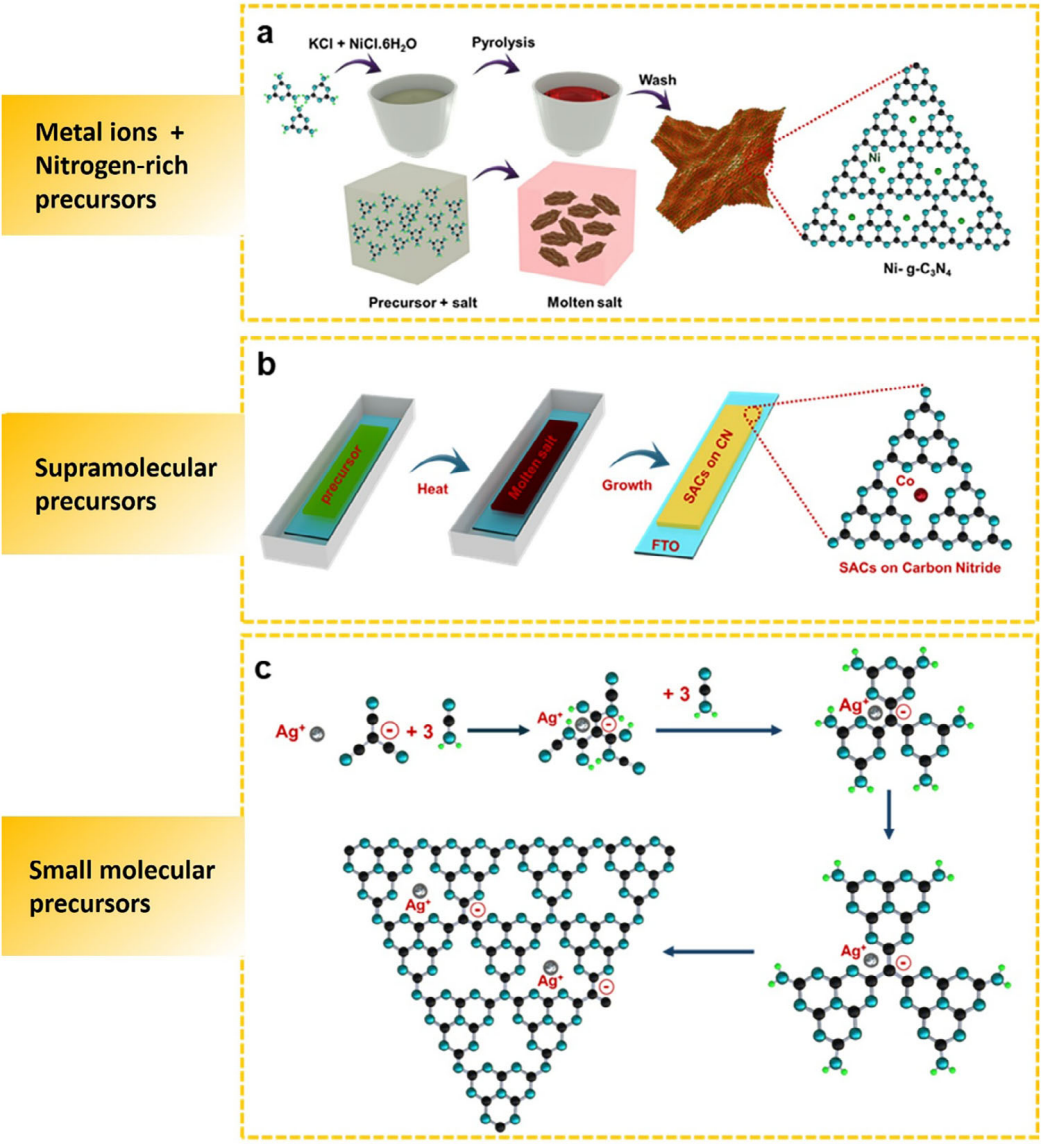
Representative examples of isolated metal SACs supported on g‐C_3_N_4_ through a pyrolysis route with various precursors. a) The synthesis of NiN_x_/g‐C_3_N_4_ catalysts. b) Schematic illustration for preparing g‐C_3_N_4_ films on FTO substrate (left), and the illustration of the structure of Co@g‐C_3_N_4_ catalysts (right). c) The synthesis of Ag@mpg‐C_3_N_4_ catalysts from silver tricyanomethanide and cyanamide. Adapted with permission.^[^ ^70^ ^]^ Copyright 2016, American Chemical Society.

1. Hybrid of metal ions and simplex nitrogen‐rich precursors: This is a widely used approach for the preparation of SACs on g‐C_3_N_4_. **Figure** **4**a shows a typical schematic illustration of the synthesis of high‐density single‐atom iron sites embedded in the g‐C_3_N_4_ frameworks.^[^ ^176^, ^177^ ^]^ Briefly, metal ions and melamine (a typical precursor for g‐C_3_N_4_) with a certain ratio were fully mixed in an agate mortar, and then the mixture was transferred into a furnace for calcination with the procedure for preparing g‐C_3_N_4_ under the protection of a nitrogen atmosphere.^[^ ^178^ ^]^ In the final products, a high density of single‐atom iron atoms was found to be embedded in g‐C_3_N_4_. This route is particularly useful in the synthesis of SACs (e.g., Na,^[^ ^179^, ^180^ ^]^ K,^[^ ^181^, ^182^ ^]^ Fe,^[^ ^69^, ^176^, ^183^ ^]^ Co,^[^ ^69^, ^184^, ^185^, ^186^ ^]^ Cu,^[^ ^66^, ^187^, ^188^, ^189^ ^]^ Mn,^[^ ^190^ ^]^ Ni,^[^ ^69^, ^191^, ^192^ ^]^ Zn^[^ ^193^ ^]^ Ru^[^ ^194^ ^]^ La,^[^ ^195^ ^]^ and K_1_Na_1_^[^ ^196^ ^]^) on g‐C_3_N_4_, and two main aspects should be taken into consideration: i) the ratio of the two precursors must be sufficient for full metal coordination during the pyrolysis process; ii) the protected atmosphere must be ventilated to prevent oxidation. Qiao and co‐workers^[^ ^66^, ^69^ ^]^ theoretically predicted that in the structure of g‐C_3_N_4_, one tri‐s‐triazine unit (C_6_N_8_) can coordinate one cobalt atom, which means that two g‐C_3_N_4_ moieties can coordinate one cobalt atom. After considering the yield of g‐C_3_N_4_ from dicyandiamide, it is expected that 3 mol. of the g‐C_3_N_4_ precursor can coordinate with 1 mol. of cobalt. To ensure coordination efficiency and account for other inevitable factors such as sublimation, it is better to excite the nitrogen‐rich precursor of g‐C_3_N_4_. For those involving a template to either enhance the porosity and surface area or obtain a certain morphology during the pyrolysis process, this method is also suitable. Liu et al.^[^ ^185^ ^]^ used NaCl as the sacrificial template, and after mixing it with dicyanamide and cobalt sources, the mixture was immersed in liquid nitrogen to allow growth into crystals. Through confinement pyrolysis and water washing, an ultrathin two‐dimensional g‐C_3_N_4_ nanosheet anchored with atomically dispersed cobalt sites was obtained. Note that the g‐C_3_N_4_‐based materials possess low conductivities, and this method can also be used for preparing other SACs materials with an elevated temperature beyond the decomposition limitation of g‐C_3_N_4_, where the SACs@g‐C_3_N_4_ phases act as the chelation anchoring intermediate to improve the single metal atom loading efficiency and the metal content. Zhao et al.^[^ ^197^ ^]^ reported a universal method to produce stable M‐NC SACs with high‐density metal‐N_x_ sites using g‐C_3_N_4_ species. During pyrolysis, melamine can polymerize to g‐C_3_N_4_ (≈500 °C) and bond with metal atoms to form M–N_x_ moieties, taking over the protection and preventing the aggregation of metal atoms, whereas the M–N_x_ moieties decomposed from M@g‐C_3_N_4_ at higher temperatures (>≈600 °C) can subsequently be deposited on the surface of the carbon support. This sequential protection strategy allows for the preparation of a wide range of carbon‐supported single‐atomic metals (e.g., Fe, Co, Cu, Mn, Ni, Mo) with high‐density metal loadings of up to 12 wt.%. Moreover, Hareesh and his co‐worker further demonstrated the possibility of g‐C_3_N_4_ in supporting abundant single‐atom sites.^[^ ^177^ ^]^ Through a high‐temperature polymerization process involving melamine, urea, and a copper salt precursor, SACs@g‐C_3_N_4_ with tunable copper loadings were successfully synthesized by systematically varying the initial Cu salt concentration. Benefiting from the rich pyridinic nitrogen content and edge defects of carbon nitride formed from melamine and urea, the Cu precursor was efficiently anchored during pyrolysis, and the Cu content in the synthesized material was as high as 28.2 wt.%, as determined by the inductively coupled plasma mass spectrometry analysis. Other SACs have also been produced similarly, such as the exclusive Ni–N_4_ sites in graphitic carbon^[^ ^198^ ^]^ and N‐doped graphene^[^ ^199^ ^]^ and Fe–N_5_ sites in N‐doped graphene.^[^ ^200^, ^201^ ^]^
2. Supramolecular precursors: The supramolecular chemistry approach provides a great opportunity for the preparation of nanostructured materials without any further templating techniques (Figure 4b).^[^ ^202^, ^203^ ^]^ Shalom and co‐workers^[^ ^39^, ^56^, ^57^, ^58^, ^154^, ^204^ ^]^ devoted much effort to the synthesis of ordered structures of g‐C_3_N_4_, including spherical macroscopic assemblies, spheres, and hollow boxes through a supramolecular chemistry approach. In addition, this approach can be applied to deposit g‐C_3_N_4_‐based materials on different substrates.^[^ ^39^, ^154^, ^205^, ^206^ ^]^ Xu et al.^[^ ^39^ ^]^ reported a liquid‐mediated pathway for the growth of continuous g‐C_3_N_4_ films on various substrates. The key point of this deposition method lies in the employment of supramolecular complexes that transform into a liquid state before direct thermal condensation into g‐C_3_N_4_ films. Zhang et al.^[^ ^205^ ^]^ taking advantage of the transformation of the liquid‐mediated phase for this supramolecular complex, designed and developed a single‐atom Ni‐sites‐embedded g‐C_3_N_4_ film. During pyrolysis, the Ni ions can be fully dissolved in the liquid phase of the organic molecules, which greatly promotes uniform and complete coordination between the metal ions and g‐C_3_N_4_ precursors, which is significantly superior to that of mechanical mixing. One of the distinct advantages is that the single metal atom loading can reach up to 5 wt.% without any other post‐treatments such as acid washing. Li et al.^[^ ^207^ ^]^ designed a supramolecular complex‐derived strategy to prepare abundant Fe single atoms on bamboo‐like carbon nanotubes. We recently reported a similar supramolecular chemistry approach, starting from a single molecular source of dicyandiamide, to fabricate single‐metal atom dispersed g‐C_3_N_4_ materials.^[^ ^208^, ^209^ ^]^ A dicyandiamidine nitriate crystal was obtained after HNO_3_ treatment of dicyandiamide, which can spontaneously transform into melamine and cyanuric acid to preorganize into a supramolecular precursor and can efficiently capture metal ions via strong chelation during the relatively complicated polycondensation reaction. The metal chelate is able to join in the formation of a supramolecular complex, playing an important role in the engineering and construction of metal ion‐modified precursors. The subsequent pyrolysis accomplishes in situ polymerization, where the confinement effect derived from the robust coordinate trap bonds guarantees the atomic dispersion of metal atoms.
3. Small molecular precursors: Metal‐containing small molecular precursor routes are considered a straightforward way to stabilize isolated metal atoms on g‐C_3_N_4_. However, these small molecules usually possess a relatively low decomposition temperature, which renders it difficult to avoid metal aggregation. Hence, the selection of molecular precursors with proper coordination structures that can directly participate in the polymerization of g‐C_3_N_4_ is a key point. For example, Chen et al.^[^ ^70^ ^]^ presented an approach using silver tricyanomethanide (AgTCM) as a reactive comonomer to co‐polymerize with cyanamide and successfully merged atomically dispersed silver and g‐C_3_N_4_ into a joint electronic system (Figure 4c). Wang et al.^[^ ^210^, ^211^ ^]^ and Wang et al.^[^ ^212^ ^]^ also prepared the Ag@g‐C_3_N_4_ by the same silver molecular precursor, and further integrated with carbon quantum dots for improving the photocatalytic activities. Gao et al.^[^ ^213^ ^]^ synthesized a copper‐melamine molecular network [Cu_2_Cl_2_(µ‐Cl)(µ‐OCH_3_)(CH_3_OH)(MA)_2_·2Et_2_O] as the starting precursor and fabricated a single Cu‐atom‐embedded g‐C_3_N_4_ material. Furthermore, a wide range of SACs have been obtained through pyrolysis with small metal molecules, including Co@g‐C_3_N_4_, through the utilization of Co(salen),^[^ ^214^ ^]^ Co(phthalocyanine),^[^ ^215^ ^]^ and Co(acetylacetonate)^[^ ^216^ ^]^ complexes, Ni@g‐C_3_N_4_ through Ni(acetate)^[^ ^217^ ^]^ complexes, Mn@g‐C_3_N_4_ through Mn(acetate)^[^ ^218^ ^]^ complexes and W@g‐C_3_N_4_ through Ammonium metatungstate hydrate.^[^ ^219^ ^]^

Among the various strategies for fabricating single atoms on g‐C_3_N_4_, the pyrolysis of a hybrid of metal ions and nitrogen‐rich precursors emerges as a particularly robust and scalable approach for achieving high metal loadings with exceptional thermal stability. This method capitalizes on the in situ formation of strong coordinative M–N_x_ bonds during the thermal polycondensation process, effectively trapping metal atoms within the developing heptazine framework. In contrast, while ALD affords unparalleled precision in site‐specific deposition, its practical application is often constrained by complexity and cost. Similarly, the conventional impregnation‐reduction method, despite its simplicity, frequently suffers from inadequate stabilization of metal species, leading to aggregation under operational conditions. Consequently, the direct pyrolysis strategy stands out for its unique combination of efficacy, scalability, and the ability to generate catalytically active sites that are both dense and structurally integral, making it the most promising pathway for industrial‐scale implementation of SACs@g‐C_3_N_4_ in advanced energy and environmental technologies.

## 3 Advanced Analysis and Theoretical Tools for SACs@g‐C_3_N_4_

To establish the structure‐activity relationship and clarify the synergistic mechanism of the designed SACs@g‐C_3_N_4_ materials, the structural characterization of the materials is essential, which represents the research foundation for subsequent predicting potential applications. Thus, the characterization methods and theoretical calculation methodology are briefly introduced, which are divided into three categories: direct visualization techniques, structure identification, and theoretical calculations.

Direct visualization technology serves as a fundamental characterization approach for identifying the spatial distribution and atomic dispersion of metal species within SACs@g‐C_3_N_4_ materials, providing primary evidence of their single‐atom nature.^[^ ^43^, ^221^ ^]^ Despite its capability to directly observe metal atoms, this methodology offers limited power in resolving the precise coordination microenvironment of active sites. Conventional imaging techniques, including Scanning Electron Microscopy (SEM), Transmission Electron Microscopy (TEM), and Atomic Force Microscopy (AFM), are generally constrained by resolution limitations that prevent clear differentiation of single‐atom features.^[^ ^222^ ^]^ Although advanced imaging strategies such as spherical aberration‐corrected high‐resolution TEM (AC‐HRTEM), Scanning Tunneling Microscopy (STM), and AC‐HAADF STEM have pushed resolution to the atomic scale, they still encounter substantial challenges in distinguishing isolated metal atoms with electron cloud densities comparable to the nitrogen‐rich carbon substrate of g‐C_3_N_4_.^[^ ^223^ ^]^ This critical limitation can be effectively overcome through coupling with Electron Energy‐Loss Spectroscopy (EELS), which provides complementary nanoscale elemental mapping and electronic structure information. When integrated with AC‐HAADF STEM, EELS enables simultaneous atomic‐scale imaging and chemical identification, offering a powerful solution for unambiguous verification of metal single atoms.^[^ ^224^ ^]^ Consequently, despite their indispensable role in preliminary characterization, visualization techniques alone remain inadequate for the comprehensive determination of coordination configurations, necessitating integration with spectroscopic methods for complete structural elucidation in SACs@g‐C_3_N_4_ systems.

To complement direct visualization techniques and gain deeper insights into the coordination environment of active centers, core structural characterization methods such as Mössbauer spectroscopy and XAS are indispensable.^[^ ^44^, ^225^ ^]^ Mössbauer spectroscopy, leveraging the resonant emission and absorption of gamma rays, is a powerful probe for deciphering the chemical states, coordination symmetry, and spin states of specific nuclides, most notably iron, in SACs@g‐C_3_N_4_ materials. Its exceptional energy resolution allows it to detect subtle changes in the nuclear energy levels caused by the surrounding electronic environment, providing fingerprints for different iron species. In parallel, XAS, comprising the X‐ray Absorption Near‐Edge Structure (XANES) and the Extended X‐ray Absorption Fine Structure (EXAFS), provides comprehensive electronic and structural information without requiring long‐range order, making it ideal for amorphous catalysts. The XANES region, particularly the pre‐edge and edge features, revealed the local geometry, electronic structure, and formal oxidation state of the absorber atom. Complementarily, EXAFS analyzes the oscillatory fine structure beyond the absorption edge to yield quantitative details on the coordination numbers, bond lengths, and chemical identities of the atoms immediately surrounding the central metal.^[^ ^226^ ^]^ Together, this suite of techniques moves beyond mere visualization to deliver a multi‐faceted, atomic‐level description of the catalytically active sites, which is fundamental for establishing robust structure‐activity relationships.

DFT calculations serve as a powerful computational approach for predicting and rationalizing the potential applications of SACs@g‐C_3_N_4_ materials, providing atomic‐level insights into their catalytic mechanisms.^[^ ^227^, ^228^ ^]^ Despite the remarkable capability of experimental techniques in characterizing structural properties, their utility in precisely forecasting catalytic performance and identifying reaction pathways remains limited. This limitation can be effectively addressed by DFT‐based approaches, which enable the precise determination of electronic structures and adsorption energies. Moreover, when integrated with machine learning algorithms, DFT calculations can rapidly screen potential SACs configurations and predict their catalytic activities for various reactions.^[^ ^229^, ^230^, ^231^ ^]^ Furthermore, computational screening through DFT can rapidly identify promising single‐atom configurations and their potential applications in energy conversion and environmental remediation. The appropriate integration of DFT with direct visualization techniques and structural identification methods, therefore establishes a comprehensive strategy for deciphering the structure‐activity relationships in SACs@g‐C_3_N_4_ systems. This synergistic approach not only validates computational predictions with experimental evidence but also enables the rational design of high‐performance single‐atom catalysts, ultimately accelerating the development of advanced SACs@g‐C_3_N_4_ materials for sustainable energy applications.

Consequently, while invaluable for mechanistic understanding, standalone DFT calculations benefit greatly from coupling with experimental validation. This combination of various approaches provides a comprehensive framework for designing high‐performance SACs@g‐C_3_N_4_ materials, enabling rational catalyst development rather than relying solely on empirical discoveries. These approaches are presented in detail in the following sections and demonstrate the promising potential in proving the atomic dispersion of active sites, determining the coordination environment, and predicting the performance for potential applications.

## 4 Emerging Applications of SACs@g‐C_3_N_4_

**Figure 5:**
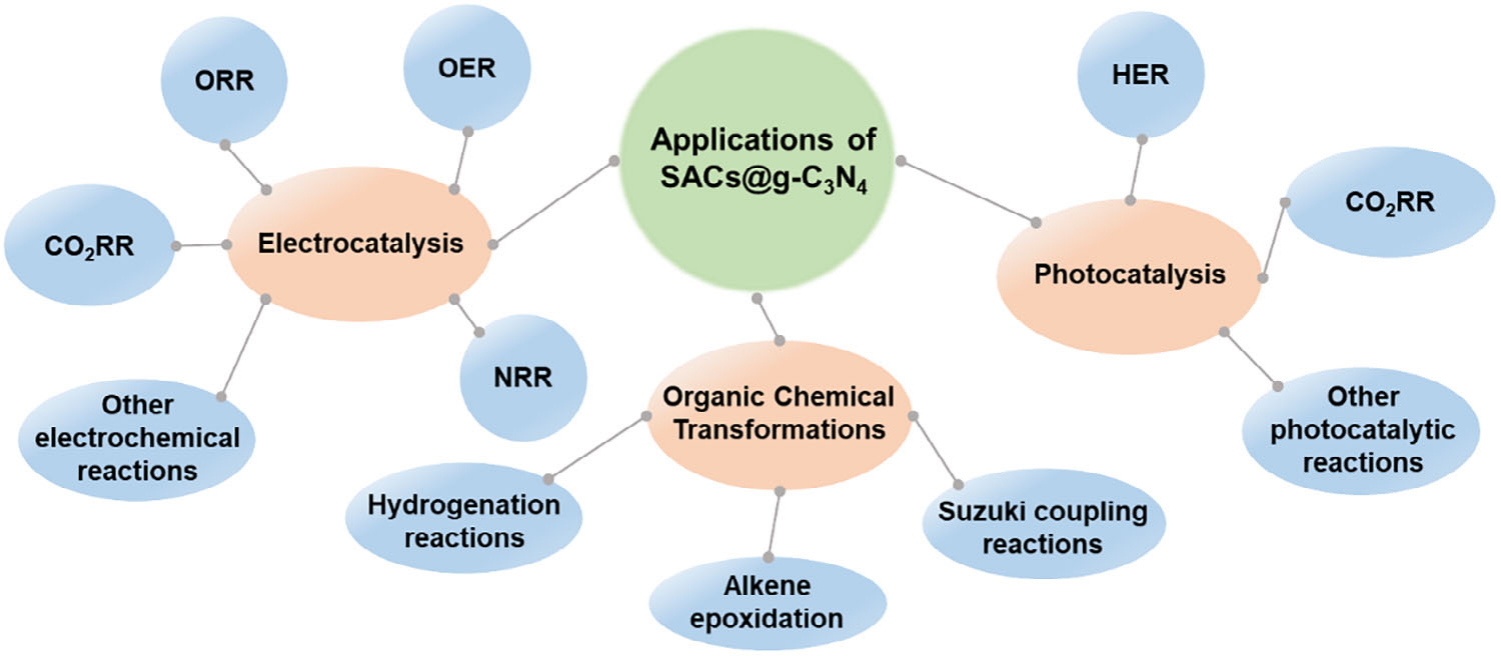
A diverse emerging applications of SACs@g‐C_3_N_4_.

In recent years, many SACs@g‐C_3_N_4_ catalysts have been reported to accelerate various reactions, ranging from typical thermochemical reactions to photochemical and electrochemical conversions. The benefits of SACs@g‐C_3_N_4_ over other conventional catalysts are multifarious and may not only lie in the atomically dispersed metal atoms but also the well‐defined active sites, distinctive coordination structures, and robust metal‐support interactions. In the following section, we introduce and summarize recent examples to demonstrate the capability and great potential of SACs@g‐C_3_N_4_ for several typical chemical conversion processes (**Figure** **5**).

### 4.1 Electrocatalysis

Electrochemical energy conversion has been considered the key component that responds to global energy demands and sustainable carbon‐neutral development goals.^[^ ^27^, ^232^, ^233^, ^234^, ^235^ ^]^ The performance of these electrochemical devices strongly depends on the properties of the materials used in each component, particularly the electrocatalysts.^[^ ^236^, ^237^ ^]^ Consequently, increasing attention has been paid to the development of highly efficient, durable, and economical electrocatalysts. However, this remains a major challenge. As stated above, SACs@g‐C_3_N_4_ electrocatalysts with unique textural features offer great promise for effectively boosting the development and commercial applications of electrochemical energy conversion technologies, such as the oxygen reduction reaction (ORR), oxygen evolution reaction (OER), CO_2_ reduction reaction (CO_2_RR), N_2_ reduction reaction (NRR) and other electrochemical reactions.

#### 4.1.1 ORR

The electrocatalytic ORR involves multistep proton‐coupled electron transfer reactions and proceeds via either a two‐electron (2e^–^) or a four‐electron (4e^–^) pathway in both alkaline and acidic media. The 2e^–^ pathway provides a green synthesis approach for the versatile clean oxidant H_2_O_2_ compared to the conventional anthraquinone redox process. The 4e^–^ pathway, which reduces O_2_ directly to H_2_O, is highly desired in various energy applications, including fuel cells and metal‐air batteries, owing to its high energy‐conversion efficiency. Pt‐based materials remain the most promising catalysts for the 4e^–^ process, but the high cost and scarcity of Pt largely limit their further use, and hence, significantly stimulate studies in the development of low‐Pt metal catalysts and Pt‐free catalysts.

(1) $$2e^{-}\mathrm{path}\mathrm{way}:O_{2}+2H^{+}+2e^{-}\to \;H_{2}O_{2}\left(\mathrm{acid}\mathrm{ic}\right)$$

(2) $$O_{2}+H_{2}O+2e^{-}\;\to \mathrm{OO}H^{-}+OH^{-}\;\left(\mathrm{alka}\mathrm{line}\right)$$

(3) $$4e^{-}\mathrm{path}\mathrm{way}:O_{2}+4H^{+}+4e^{-}\;\to H_{2}O\;\left(\mathrm{acid}\mathrm{ic}\right)$$

(4) $$O_{2}+H_{2}O+4e^{-}\;\to 4OH^{-}\;\left(\mathrm{alka}\mathrm{line}\right)$$

Benefited from the abundant coordinated nitrogen species and tunable defect properties on g‐C_3_N_4_, the coordination environment of the M–N_x_ site can be precisely modulated to achieve a moderate d‐band center of active metal centers, which achieves a moderate adsorption energy for the various reaction intermediates, thereby guiding the reaction toward the 2e^–^/4e^–^ pathway.^[^ ^238^, ^239^, ^240^, ^241^, ^242^ ^]^ For example, Li and co‐workers^[^ ^243^ ^]^ reported a highly active and stable isolated single‐atom Fe/N‐doped porous carbon (Fe ISAs/CN) catalyst with a Fe–N_4_ active center, which shows a half‐wave potential (E_1/2_) of 0.9 *V*_RHE_, even better than that of commercial Pt/C (≈0.84 V_RHE_) in an alkaline solution. Through a controlled experiment of occupying and releasing the atomically dispersed Fe–N_4_ sites, it is clear that the active center on Fe ISAs/CN for ORR is the isolated Fe–N_4_ coordination sites. However, recent theoretical predictions suggest that the Fe–N_4_ sites are not the centers with the highest activities because of their strong interactions with *OH and *O_2_ intermediates. Guo and coworkers^[^ ^244^ ^]^ introduced a template casting strategy that atomically disperses FeN_2_ moieties onto the surface/edges of N‐doped ordered mesoporous carbon (FeN_2_/NOMC) for boosting ORR electrocatalysis. Characterization by EXAFS and Mössbauer spectroscopy validated that the coordination numbers of Fe and N are 2.0 in FeN_2_/NOMC architectures. The unique textural features make FeN_2_/NOMC exhibit superior ORR performance in alkaline solutions with an E_1/2_ of 0.863 *V*_RHE_, suggesting better activity than that of Fe–N_4_. Further DFT computations confirmed that Fe–N_2_ outperforms Fe–N_4_ because Fe–N_2_ interacts more weakly with *OH and *O_2_ intermediates and exhibits enhanced electron transport. In another study, Yin et al.^[^ ^245^ ^]^ modulated the metal–N coordination environment by altering the pyrolysis temperature and obtained Co single‐atoms/N‐doped porous carbon (Co SAs/N‐C) catalysts with planar Co–N_4_ and Co–N_2_ coordination sites, respectively. DFT calculations and experimental evaluation revealed that the Co SAs/N‐C with Co–N_2_ center possesses higher activity toward ORR than the Co–N_4_ center in alkaline media, which originates from the stronger interaction of peroxide with Co–N_2_ species than Co–N_4_ species, promoting the 4e^–^ ORR process for enhanced performance. In other words, precise control of the coordination environment and geometric configurations at the atomic scale can be used to tune the catalytic properties and deeply understand the ORR mechanism of single‐atom M–N_x_/C catalysts.^[^ ^243^, ^246^, ^247^ ^]^ However, knowledge of the nature of the active sites in these single‐atom M–N_x_/C catalysts is insufficient because of the complexity of the various nanostructures and metal coordination compositions.

**Figure 6:**
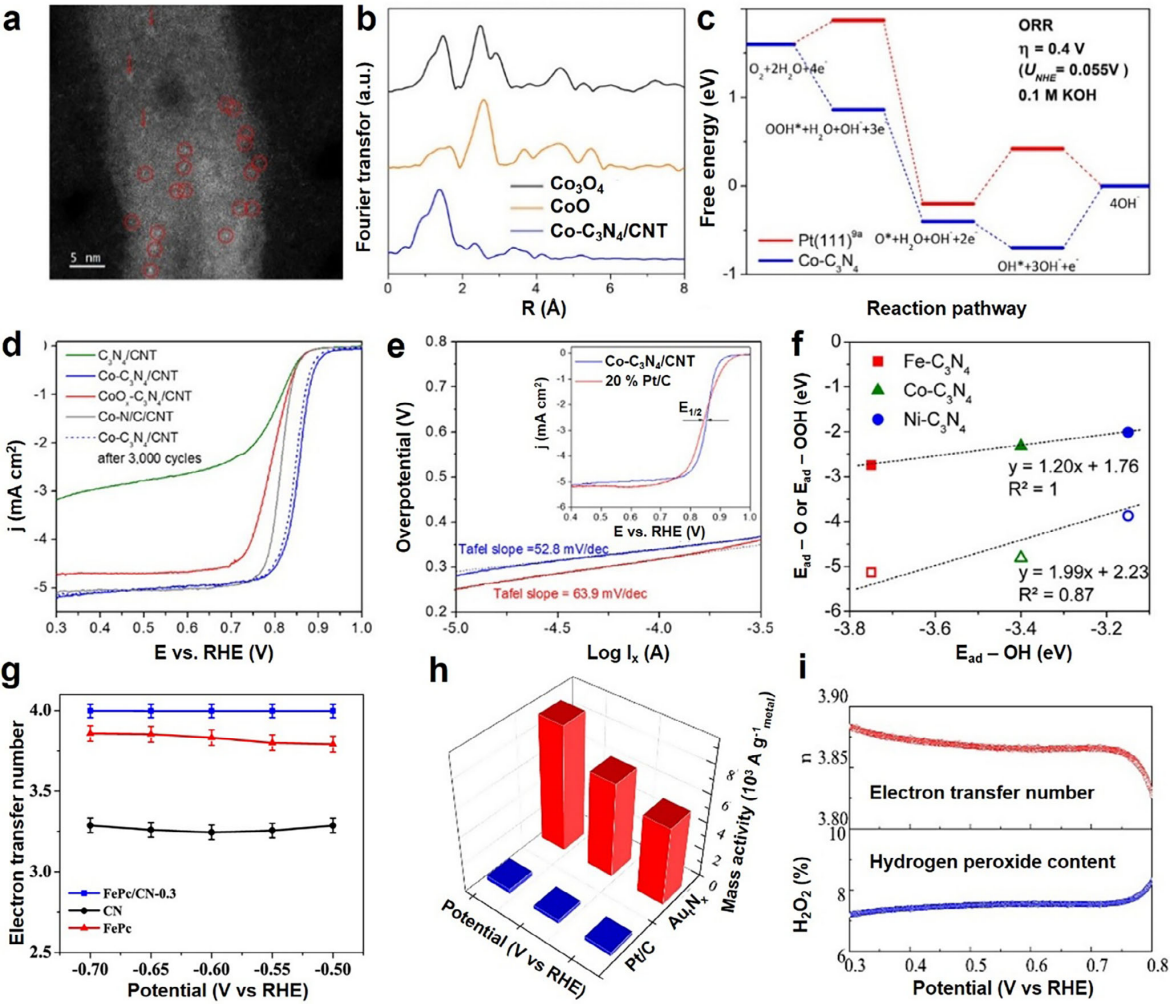
a) HAADF‐STEM image of Co‐C_3_N_4_/CNT; (b) FT‐EXAFS at the Co K‐edge of Co‐C_3_N_4_/CNT, CoO, Co_3_O_4_ samples; c) Free energy diagram of ORR on Co‐C_3_N_4_ and Pt (111) surfaces; d) ORR polarization curves of different Co‐based catalysts; e) Tafel plots calculated from polarization curves (inset) of Co‐C_3_N_4_/CNT and Pt/C; f) Scaling relationship of E_ad_–OH* versus E_ad_–OOH* (filled symbols) or E_ad_–OH* versus E_ad_–O* (open symbols) on M‐C_3_N_4_ models. Reproduced with permission.^[^ ^69^ ^]^ Copyright 2017, American Chemical Society. g) The results for calculation of electron transfer number of CN, FePc, and FePc/CN–0.3 nanocomposites during the ORR based on the RRDE measurements. Reproduced with permission.^[^ ^169^ ^]^ Copyright 2019, American Chemical Society. h) Mass activity of ORR for Pt/C and Au_1_N_x_@C_3_N_4_; i) Electron transfer number (n; top) and H_2_O_2_ yield (bottom) for Au_1_N_x_@C_3_N_4_; Reproduced with permission.^[^ ^130^ ^]^ Copyright 2018, Elsevier.

Point to this, Qiao and co‐workers^[^ ^69^ ^]^ reported a range of g‐C_3_N_4_ coordinated single‐site transition metal (M‐C_3_N_4_) catalysts, where the support of g‐C_3_N_4_ not only provides a large number of homogeneous nitrogen coordinators for securely anchoring metal atoms but also offers more precise information for the identification of catalytically active sites. As a preliminary trial, the Co‐C_3_N_4_ catalyst was comprehensively investigated via DFT calculations and experimental measurements. As shown in **Figure** **6**a, the atomic Co components (marked with red circles) were distributed evenly across the g‐C_3_N_4_ matrix. The Fourier‐transformed EXAFS (FT‐EXAFS) of Co‐C_3_N_4_ exhibited a main peak at 1.40 Å in the *R* space that was assigned to Co–N scattering paths, while no Co–O and Co–Co scattering paths were detected, suggesting the presence of mononuclear Co centers in a relatively long‐range order structure (Figure 6b). Further fitting analysis revealed the precise formation of Co–N_2_ moieties in the Co‐C_3_N_4_ catalyst, which can be reasonably assumed to be active sites for the ORR. Theoretical calculations confirm that the Co–N_2_ sites contribute to the rate‐determining step of OH* desorption, resulting in energy consumption close to that of the benchmarked Pt (111) surfaces under the same conditions (Figure 6c). Subsequently, considering the electron conductivity of Co‐C_3_N_4_, the as‐obtained Co‐C_3_N_4_ was integrated with CNT. The overall catalyst shows excellent ORR performance with an onset potential of 0.9 V_RHE_ in alkaline media, comparable with commercial Pt/C catalysts judged on both overpotentials and Tafel slope values (Figure 6d,e). DFT calculations were conducted to establish design principles for different M‐C_3_N_4_ systems and evaluate their ORR activity from a molecular perspective (Figure 6f). Liu et al.^[^ ^248^ ^]^ reported an outstanding ORR performance for Fe‐g‐C_3_N_4_ supported on a hierarchical porous N‐doped carbon polyhedral derived ZIF‐8 framework (HPNCP), which also provided an appreciable long‐term durability after 5000 cycles. Additionally, Shi et al.^[^ ^169^ ^]^ fabricated a “host‐guest” structure where single Fe centers are hosted on the surface of g‐C_3_N_4_ by π‐electron conjugated interactions. The resultant FePc/CN exhibited better ORR activity and durability in alkaline solutions than the individual Fe centers on FePc and pristine g‐C_3_N_4_. The rotating ring‐disk electrode (RRDE) method confirmed that g‐C_3_N_4_ played a pivotal role in facilitating the overall catalyst proceeds predominantly via the desired 4e^–^ pathway, thus effectively improving the energy‐conversion efficiency (Figure 6g). Liu et al.^[^ ^130^ ^]^ reported an atomic Au_1_N_x_ single‐site confined on g‐C_3_N_4_ support as an efficient and durable ORR electrocatalyst (Figure 6e). The strong charge‐transfer interactions between Au and g‐C_3_N_4_ make the atomic Au_1_N_x_ site possess a unique valence state of Au^1+^, facilitating the enhanced adsorption of oxygen and favoring the cleavage of O–O bond and allow the oxygen reaction through a favorable 4e^–^ involved process (Figure 6i). Furthermore, the periodic structure of g‐C_3_N_4_ provides a perfect coordination environment via the out‐of‐plane sp^3^ interactions, and this impervious confinement effect ensures that the atomic Au_1_N_x_ single‐site shows durable stability during the catalytic process. Consequently, Au_1_N_x_/g‐C_3_N_4_ exhibits prominent ORR performance with a high mass activity of ∼9000 A g_Au_^−1^, which is far superior to the benchmark Pt/C materials. Other isolated metal atoms stabilized on g‐C_3_N_4_ were widely studied as ORR electrocatalysts in the past few years, such as Cu@g‐C_3_N_4_,^[^ ^249^ ^]^ Co@g‐C_3_N_4_,^[^ ^250^ ^]^ which demonstrates the great potential of g‐C_3_N_4_ supports in anchoring atomic‐level transition metal active sites and well‐defined and periodic structures, offering a more precise platform for identifying the true nature of the active centers, thereby overcoming the complexity often encountered in other M–N_x_/C materials.

It is important to note that g‐C_3_N_4_ is enriched in both pyridinic and graphitic nitrogen moieties, which are considered the active nitrogen species for electrocatalysis, rendering it one of the most promising metal‐free catalysts. However, the low electron conductivity resulting from the intrinsic porous structure of g‐C_3_N_4_ materials constitutes a major obstacle to their practical application. In the past few years, tremendous research has been reported on designing and developing productive strategies to realize the use of g‐C_3_N_4_ in electrocatalysis. Typical strategies for application in ORR include the chemical integration of g‐C_3_N_4_ with carbon materials,^[^ ^251^, ^252^, ^253^, ^254^, ^255^ ^]^ fabrication of charge transfer paths by introducing porous structures,^[^ ^53^, ^256^, ^257^, ^258^ ^]^ optimization of the electronic structure via doping with heteroatoms.^[^ ^73^, ^259^, ^260^, ^261^, ^262^ ^]^ All these functionalizations yielded a positive response, which suggests that the textural features of g‐C_3_N_4_ significantly favor the electrocatalytic ORR process. Combined with the above‐mentioned benefits of g‐C_3_N_4_ on the precise control of the coordination environment of the metal and identification of catalytically active sites at an atomic scale,^[^ ^69^, ^263^ ^]^ several recent studies have opened up a new possibility in exploiting g‐C_3_N_4_ as the interphase for effectively stabilizing single metal atoms on the desired substrates.

**Figure 7:**
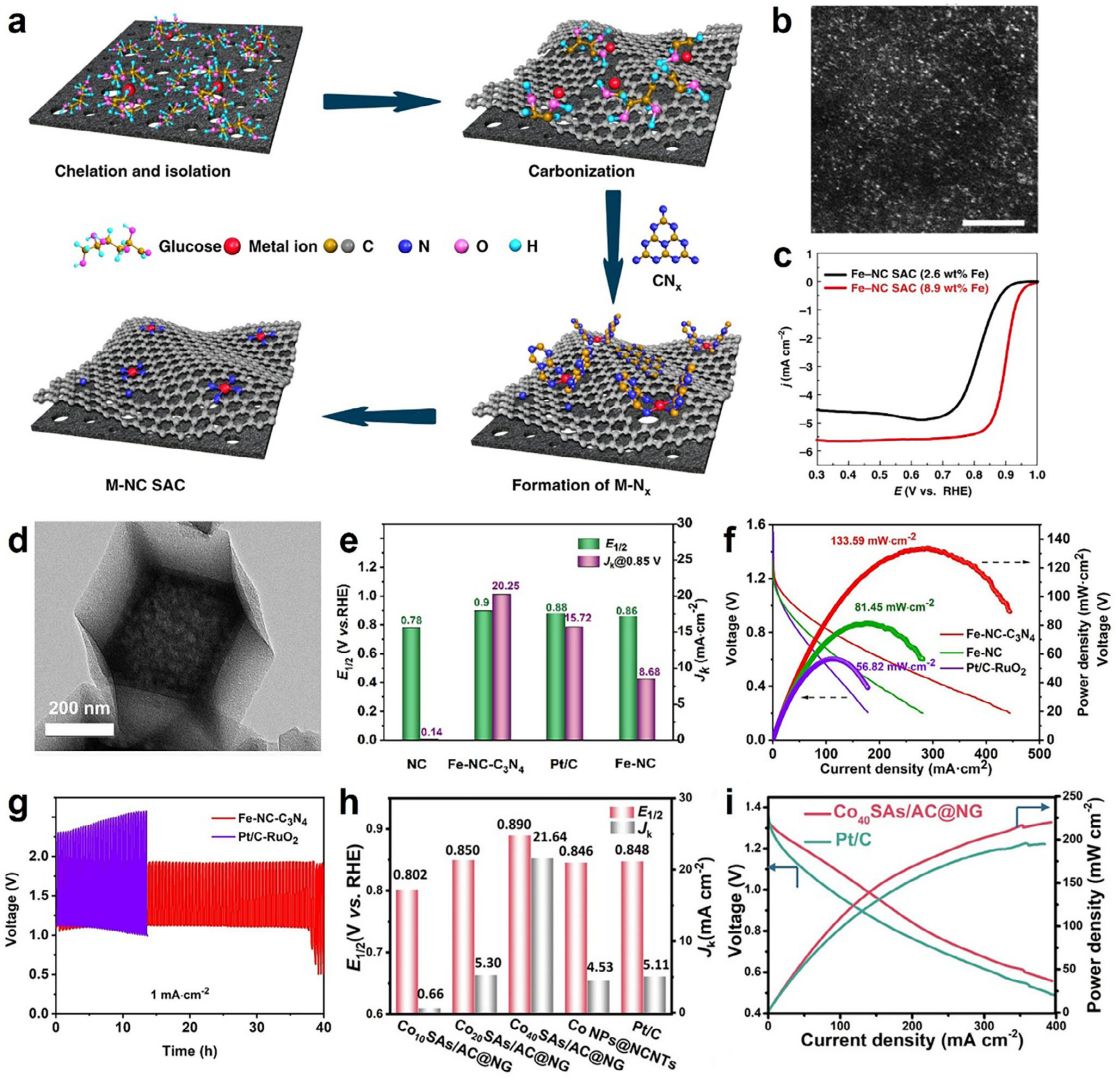
a) Illustration of the cascade anchoring strategy for the general production of high‐loading M–NC SAC. b) HAADF–STEM image of Fe–NC SAC. c) Steady‐state ORR polarization curves of Fe–NC SAC with different atomic Fe loading. Reproduced with permission.^[^ ^197^ ^]^ Copyright 2019 Springer Nature. d) TEM images of Fe‐NC‐C_3_N_4_ materials. e) Comparison of E_1/2_ values ​​and kinetic current density *J*_k_ values ​​of Fe‐NC‐C_3_N_4_ and reference sample at 0.85 V. f) Discharge polarization curves of liquid ZAB and g) discharge‐charge curves of flexible ZAB between Fe‐NC‐C_3_N_4_ and reference samples. Reproduced with permission.^[^ ^264^ ^]^ Copyright 2024 Elsevier. h) The ORR performances of the *J*_k_ and E_1/2_ between Co_40_SAs/AC@NG and reference samples. i) Polarization curves and power‐density comparison between Co_40_SAs/AC@NG and Pt/C catalysts. Reproduced with permission.^[^ ^265^ ^]^ Copyright 2022 John Wiley and Sons.

For instance, Zhao et al.^[^ ^197^ ^]^ reported a cascade anchoring strategy to prepare diverse SACs with atomic metal–N_x_ moieties anchored on carbon supports. During pyrolysis synthesis, the in situ formation of the g‐C_3_N_4_ phase was used to effectively stabilize the metal atoms at the atomic scale, and the subsequent degradation of M@g‐C_3_N_4_ directly resulted in the uniform dispersion of M–N_x_ moieties on the surface of the carbon support (**Figure** **7**a). As a representative sample, Fe‐NC SACs exhibit an unprecedented high metal loading of up to 12.1 wt.%, which was confirmed by the HAADF‐STEM and statistical analysis, suggesting that these high‐density Fe atoms were mainly in the single‐atomic state (Figure 7b). When tested in alkaline media, these as‐obtained Fe‐NC SACs show a positively shifted E_1/2_ of 0.90 *V*_RHE_ and a kinetic mass current of 100.7 A g^−1^ at 0.90 *V*_RHE_, which are 50 mV and 65 A g^−1^ higher than those for the Pt/C catalyst, respectively. A comparison with and without SCN^–^‐poisoned, as well as a low Fe loading, corroborates that the Fe–N_x_ sites in Fe‐NC SACs are efficient active centers for delivering a high ORR property, and the loading of Fe–N_x_ sites greatly affects the activity (Figure 7c). In another study, Xu and co‐workers reported a strategy consisting of space confinement and pore‐making engineering with g‐C_3_N_4_ as the source of corrosive gas molecules, successfully fabricated a N‐doped Fe‐NC‐C_3_N_4_ catalyst.^[^ ^264^ ^]^ The introduction of g‐C_3_N_4_ served not only as a gasification and protective agent to prevent the aggregation of Fe species, but also as an efficient pore‐forming agent to increase the pore size distribution on the carbon skeleton (Figure 7d). Benefited from the abundant pore distribution and accessible highly active FeN_4_ sites, the constructed Fe‐NC‐C_3_N_4_ materials demonstrates exceptional bifunctional oxygen electrocatalytic activity in alkaline media, achieving a remarkable E_1/2_ of 0.90 *V*_RHE_ for the oxygen reduction, superior than commercial Pt/C benchmark (0.88 *V*_RHE_) (Figure 7e), while maintaining a low overpotential of merely 0.305 *V*_RHE_ at 10 mA·cm^−2^ for the oxygen production. Thus, the lab‐made liquid zinc‐air battery (ZAB) with Fe‐NC‐C_3_N_4_ as the air cathode exhibited superior performance, achieving a peak power density of 133.59 mW·cm^−2^ and a specific capacity of 882.58 mAh·g^−1^, which substantially surpasses those obtained with the commercial Pt/C‐RuO_2_ benchmark (56.82 mW·cm^−2^ and 643.87 mAh·g^−1^, respectively) (Figure 7f). Moreover, the Fe‐NC‐C_3_N_4_‐based flexible ZAB devices also display an excellent long lifespan of 37 h with 111 cycles, significantly superior to Pt/C‐RuO_2_, indicating excellent bifunctional catalyst performance and application prospects in wearable devices (Figure 7g). Furthermore, Liu and colleagues also demonstrated this innovative application of g‐C_3_N_4_ as a sacrificial template for synthesizing highly active bifunctional SACs for ORR/OER,^[^ ^227^ ^]^ which subsequently exhibited outstanding performance in ZAB applications, highlighting its significant potential in advanced energy storage systems.

In another study, Xu and co‐workers developed a facile self‐sacrificing template strategy for synthesizing a Co single atom along with Co atomic clusters co‐anchored on porous‐rich nitrogen‐doped graphene (Co SAs/AC@NG) through thermally annealing the compounds of dicyandiamide and a certain amount of CoCl_2_·6H_2_O under a two‐step pyrolysis process.^[^ ^265^ ^]^ HAADF‐STEM and EXAFS studies corroborated the successful introduction of Co species with the co‐existence of Co single atoms and clusters in the structure, which demonstrated a high ORR property in an alkaline solution. The polymerization behavior of dicyandiamide into layered g‐C_3_N_4_ under low temperature worked as a temporary layer template for subsequent carbonization, which could provide a large amount of nitrogen‐containing small molecules, playing a critical role in the formation of 2D sheet structure of Co SAs/AC@NG, and the high Cu loading of 14.0 wt.%. As shown in Figure 7h of the ORR performance, the optimized Co_40_SAs/AC@NG catalysts display the best performance among the related materials, with E_1/2_ of 0.89 V, *J*_k_ of 17.07 mA cm^−2^, and mass activity of 305 A g^−1^ at 0.85 V, outperforming the commercial Pt/C (E_1/2_ of 0.84 V, *J*_k_ of 4.84 mA cm^−2^, and mass activity of 60.5 A g^−1^). Additionally, the home‐made ZAB based on the Co_40_SAs/AC@NG also delivered encouraging practical application potentials, which displayed the maximum power density of 221 mW cm^−2^ and high cycling stability (Figure 7i). This result is in accordance with the findings on the M–N_x_/C system.^[^ ^243^, ^244^, ^245^ ^]^ In addition to these two studies, other SACs synthesized with the assistance of g‐C_3_N_4_ have also been demonstrated as excellent ORR electrocatalysts with a favorable 4e^–^ pathway that has comparable performance in contrast to the benchmark Pt/C catalysts.^[^ ^263^, ^266^, ^267^, ^268^, ^269^, ^270^, ^271^ ^]^

In summary, g‐C_3_N_4_ is an exceptional support for single‐atom ORR catalysts due to its homogeneous nitrogen coordinators that create well‐defined, uniform active sites. This defined structure not only facilitates high activity but also enables precise identification of active centers, overcoming the complexity of other M–N_x_/C materials. The strong metal‐support interaction optimizes electronic states, enhancing oxygen intermediate adsorption and promoting the efficient 4–electron pathway. Additionally, the robust coordination environment and confinement effect ensure outstanding catalyst durability and stability during operation.

#### 4.1.2 OER

The electrocatalytic OER is considered the reverse reaction of the ORR, which is another important process in energy‐conversion technologies.^[^ ^272^, ^273^ ^]^ Active and stable electrocatalysts are urgently needed to achieve favorable reactions to improve the intrinsically sluggish kinetics of the OER. Jiang and co‐workers^[^ ^274^ ^]^ simulated transition metal single‐atoms supported on g‐C_3_N_4_ as advanced OER electrocatalysts using DFT calculations. Transition metal single atoms provide open sites to bind OH^–^ in alkaline media for further activation, acting as efficient active centers. Interestingly, the redox reaction of the transition metal centers occurs and is involved in every OER step, which plays a key role in determining catalytic performance.

**Figure 8:**
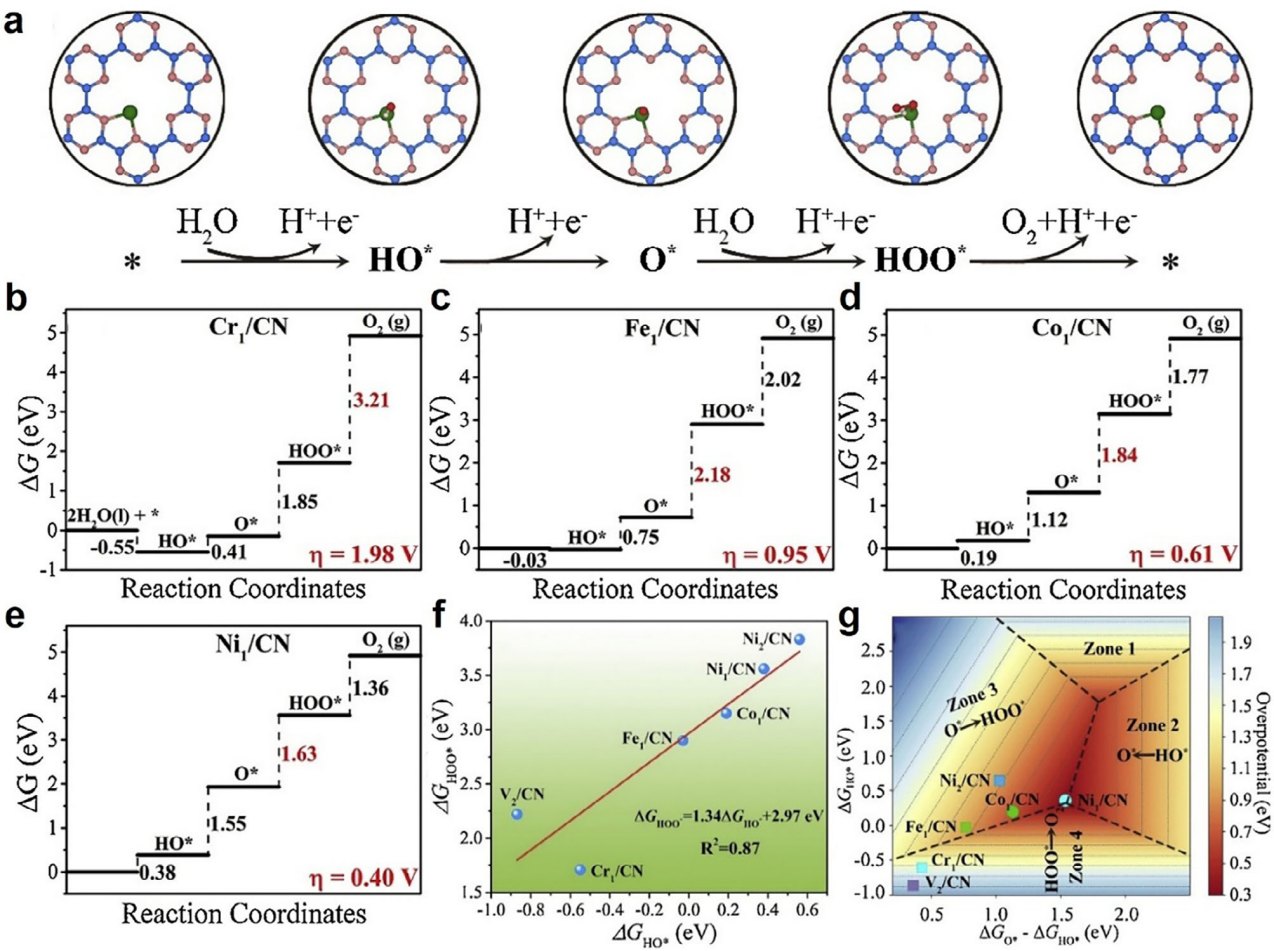
a) Reaction scheme of the OER process. Free energy diagram for b) Cr_1_/g‐CN, (c) Fe_1_/g‐CN, d) Co_1_/g‐CN, e) Ni_1_/g‐CN for OER at U = 0, where the elementary reaction with ΔG in red represents the rate‐determining step. f) Scaling relationship for Gibbs free energies of HOO* (ΔG_HOO*_) and HO* (ΔG_HO*_). g) OER activity volcano showing the overpotentials as a function of Gibbs free energies of the reaction intermediates. The color bar represents the value of OER overpotential. The black dashed lines divide the 3D activity volcano into four zones based on the potential‐determining step. Reproduced with permission.^[^ ^275^ ^]^ Copyright 2020 Elsevier.

In the recent report, Dai and co‐workers^[^ ^275^ ^]^ demonstrated a consistent result and revealed that the local structure and chemical environment of the active centers were highly dependent on the type of transition metal, which has paramount effects on OER activity. Isolated Co or Ni on g‐C_3_N_4_ was found to be energetically favorable for the rate‐determining step of the formation of OOH* for the OER process in these M@g‐C_3_N_4_ systems (**Figure** **8**a–e). The other studied metals, including V, Fe, and Cr, showed strong interactions with HO*, accounting for the poisoning phenomenon of the active centers, which is adverse to the OER process. Further DFT calculations revealed that the OER activities of M@g‐C_3_N_4_ were correlated with the *d*‐band centers of the metal atoms, and a lower *d*‐band center is associated with a weaker binding between the metal active sites and the adsorbates. Thus, a principle for improving the OER activity is established: adjusting the *d*‐band centers toward a volcano value of –2.76 eV for M@g‐C_3_N_4_. All these theoretical calculations provide a new clue for OER catalyst design, and subsequent experimental investigations validated this prediction (Figure 8f,g). Diverse transition metal single atoms were stabilized in the intestine structure of g‐C_3_N_4_ as a high‐performance OER catalyst.^[^ ^69^ ^]^ As expected, the Co@g‐C_3_N_4_ possesses excellent OER activity with an onset potential of ≈1.5 V_RHE_ and a kinetic area current of 10 mA cm^−2^ at a potential of 1.61 *V*_RHE_, comparable to those of IrO_2_ benchmarks. In addition, Co@g‐C_3_N_4_ exhibited durable stability with no obvious deterioration, even after 3000 cycles. In conclusion, g‐C_3_N_4_ furnishes an ideal substrate with uniform coordination motifs for anchoring single‐atom active sites. This precise configuration permits the rational design of active centers to adjust the adsorption energy of the OER‐related oxygen‐containing intermediates, thereby optimizing the reaction pathway and ultimately yielding catalysts with remarkable activity and robust stability.

#### 4.1.3 CO_2_RR

Sustainable carbon cycle utilization has received intensive attention recently under the double pressures of both environmental protection and market economies. The electrochemical reduction of CO_2_ into value‐added products offers a practical way to address this challenge and is an effective approach to relieve CO_2_ emissions and accumulation.^[^ ^276^, ^277^, ^278^ ^]^ However, the inert C═O bonds require a high reduction potential to overcome the energy barriers during the conversion process, which may cause competition with the H_2_ evolution side reaction, resulting in low conversion efficiency and poor selectivity of the product.^[^ ^279^, ^280^ ^]^ Thus, much effort has been devoted to the design and development of efficient electrocatalysts with fast kinetics and high selectivity.

**Figure 9:**
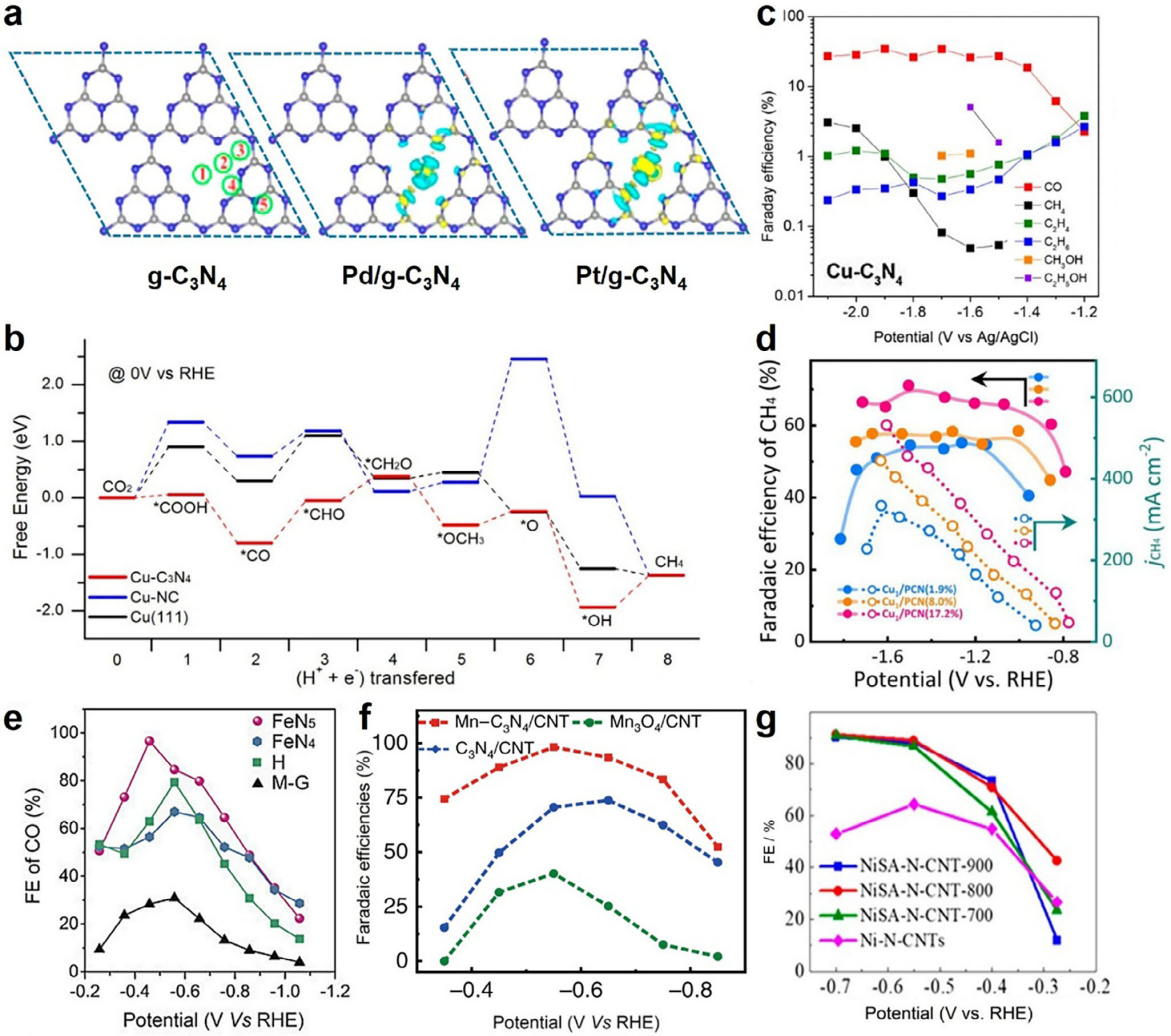
a) Optimized structure of pristine g‐C_3_N_4_ and plots of 3D differential charge densities of Pd/g‐C_3_N_4_ and Pt/g‐C_3_N_4_. Reproduced with permission.^[^ ^281^ ^]^ Copyright 2016 American Chemical Society. b) Free energy diagram of the CO_2_ reduction pathway to CH_4_ on Cu‐C_3_N_4_, Cu‐NC, and Cu(111). c) Faradaic efficiencies of various products on Cu‐C_3_N_4_. Reproduced with permission.^[^ ^66^ ^]^ Copyright 2017 American Chemical Society. d) Comparison of FE_CH4_ and *j*_CH4_ with different loading amounts of Cu_1_/PCN (x%). Reproduced with permission.^[^ ^102^ ^]^ Copyright 2025 John Wiley and Sons. e) Faradaic efficiencies for CO on FeN_5_ catalyst and reference catalysts. Reproduced with permission.^[^ ^200^ ^]^ Copyright 2019 John Wiley and Sons. f) Faradaic efficiencies for CO on Mn‐C_3_N_4_/CNTt and reference catalysts. Reproduced with permission.^[^ ^228^ ^]^ Copyright 2020 Springer Nature. g) Faradaic efficiencies for CO on NiSA‐N‐CNT catalysts and Ni‐N‐CNTs. Reproduced with permission.^[^ ^282^ ^]^ Copyright 2018 American Chemical Society.

Among them, SACs gradually stand out owing to their high ratio of low‐coordinated metal atoms, special electronic structures, and technically uniform structures, which energetically favor the catalytic process, and importantly, are beneficial for the understanding of structure‐activity relationships. Gao et al.^[^ ^281^ ^]^ constructed a structural model in which a single Pt or Pd atom was anchored on g‐C_3_N_4_ and investigated their CO_2_ reduction behavior via DFT calculations (**Figure** **9**a). During CO_2_ reduction, the isolated metal atoms functioned as the active centers, with the preferred product on the Pd/g‐C_3_N_4_ catalyst being HCOOH, whereas that on Pt/g‐C_3_N_4_ was CH_4_. Qiao et al.^[^ ^66^ ^]^ reported atomically dispersed Cu atoms on g‐C_3_N_4_ for efficient CO_2_RR. Figure 9b displayed the free energy diagram of the CO_2_ reduction pathway to CH_4_ on different Cu‐based catalysts, where the Cu (111) slab surface and single‐site Cu supported on nitrogen‐doped graphene (Cu‐NC) were studied for comparison. The reaction pathway of the first four‐electron transfer steps included the commonly considered rate‐determining steps for CO_2_ reduction, hydrogenation of *CO_2_ to *COOH, and *CO to *CHO, and was found to proceed with identical reaction intermediates on all three catalysts. However, it diverged from the fifth proton and electron pair transfer process. The *OCH_3_ group was formed with oxygen, and the isolated CH_4_ molecule was produced in the case of Cu‐g‐C_3_N_4_ and Cu (111) surfaces, while on the Cu‐NC surface, another two hydrogenation steps of the generation of *CH_2_OH and *CH_2_ were needed to form the final CH_4_ product. Despite the different reduction pathways, Cu‐g‐C_3_N_4_ showed the strongest binding to reaction intermediates and hence the lowest free‐energy pathway, which led to a more facile CO_2_ reduction with the only rate‐determining step of *CHO. The d‐orbital position toward the Fermi level of copper was significantly shifted when it was embedded in g‐C_3_N_4,_ and an intramolecular synergistic effect was induced toward CO_2_RR; both the metallic site of Cu and the neighboring C site worked synergistically as active centers during the reaction process. As a result, a wide variety of C_2_ products (C_2_H_5_OH, C_2_H_6_, and C_2_H_4_) and high selectivity for deep reduction products (CH_4_ and CH_3_OH) were achieved on the Cu‐g‐C_3_N_4_ electrocatalyst (Figure 9c). To further improve the CH_4_ production efficiency of Cu‐N‐C catalysts, Feng et al. utilized a sequential ion exchange strategy to prepare well‐defined Cu SACs periodically dispersed on a layered g‐C_3_N_4_ substrate with the 17.2 wt.% Cu loading, in which the diagonally coordinated N–Cu–N configuration hosted low‐valent Cu^δ+^ centers,^[^ ^102^ ^]^ verifying the excellent potential of g‐C_3_N_4_ for synthesizing high‐loading catalysts. Experimental results and theory analysis elucidated that the constructed 2*Cu–N_2_ spatial configuration within the Cu_1_/PCN (17.2%) catalyst displayed a strong *CO binding affinity, effectively suppressing the desorption of *CO and the competitive C–C coupling, which delivered a superior CH_4_ production efficiency of 71.1% with partial current density of 426.6 mA cm^−2^ at –1.50 *V*_RHE_ (Figure 9d).

As mentioned in the ORR section, g‐C_3_N_4_ is typically used as an assisted interphase to effectively realize the deposition of SACs on the desired substrates. For example, using prolonged thermal pyrolysis of hemin and melamine molecules on graphene, Zhang et al.^[^ ^200^ ^]^ reported FeN_5_ SACs with an extremely high Faradaic efficiency exceeding 97% for achieving the CO_2_ to CO conversion at a low overpotential of 0.35 V (Figure 9e). The atomically dispersed FeN_5_ active site possesses an additional axial ligand coordinated to FeN_4_, which contributes to the depletion of the electron density of the Fe 3d orbital and decreases the Fe–CO π back donation, thereby enabling the rapid desorption of CO and high selectivity for CO production. Zhang and co‐workers^[^ ^228^ ^]^ rationally designed a Mn‐coordinated g‐C_3_N_4_ material with Mn–N_3_ sites (Mn‐C_3_N_4_/CNT) as an efficient catalyst for catalyzing CO_2_RR. During the synthesis process, dicyandiamide (DCD) was applied as the precursor for g‐C_3_N_4_, which was proposed to provide abundant nitrogen species for the anchoring of Mn atoms to the maximum extent and prevent the aggregation and oxidation of Mn species. HADDF‐STEM observation and FT‐EXAFS analysis clearly confirmed the presence of abundant and exclusive Mn–N_3_ sites, which were identified as the active centers and enhanced the activity and selectivity for CO_2_ to CO conversion, exhibiting a faradaic efficiency reached 98.8% with a *j*_CO_ of 14.0 mA cm^−2^ under 0.44 *V*_RHE_ in CO_2_‐saturated KHCO_3_ electrolyte, even a higher *j*_CO_ of 29.7 mA cm^−2^ under ionic liquid solution (Figure 9f). Thermodynamic DFT calculations combined with *in‐situ* XAS revealed that the Mn–N_3_ active sites predominantly contributed to reducing the energy barrier and boosting charge transfer for CO_2_ conversion, especially for the formation of the key intermediate *COOH, thus positively influencing the activity and selectivity for CO production and suppressing competitive hydrogen evolution reactions. Zhao et al.^[^ ^282^ ^]^ reported another atomic Ni catalyst with a bamboo‐shaped tubular morphology that was transformed from a stacked and layered Ni@g‐C_3_N_4_ structure via a solid‐to‐solid curling or rolling‐up mechanism. This atomically dispersed Ni catalyst displayed high‐efficiency CO_2_RR activity with a remarkable turnover frequency (TOF) of 11.7 s^−1^ at –0.55 *V*_RHE_ and great Faradaic efficiency exceeding 90% at –0.55 *V*_RHE_ to –0.7 *V*_RHE_ toward CO production (Figure 9g). Serves as an exceptional platform, g‐C_3_N_4_ enables the precise stabilization of various single metal centers. Furthermore, through the rational design of active sites on carbon nitride, the binding strength of key reaction intermediates (such as *COOH and *CO) can be effectively optimized, steering reaction pathways towards specific valuable products.

#### 4.1.4 NRR

The activation of dinitrogen (N_2_) to synthesize ammonia (NH_3_) is a requisite transformation for sustaining life on Earth. To date, industrial‐scale NH_3_ production has primarily progressed by the Haber‐Bosch process with heterogeneous Fe‐based catalysts under drastic reaction conditions, which is greatly plagued by high energy demand and fossil fuel‐derived CO_2_ emissions.^[^ ^283^, ^284^, ^285^ ^]^ The electrochemical NRR, proposed as a clean and sustainable alternative for NH_3_ synthesis, has received considerable attention because it offers the promise to be conducted with electrical energy at ambient conditions.

**Figure 10:**
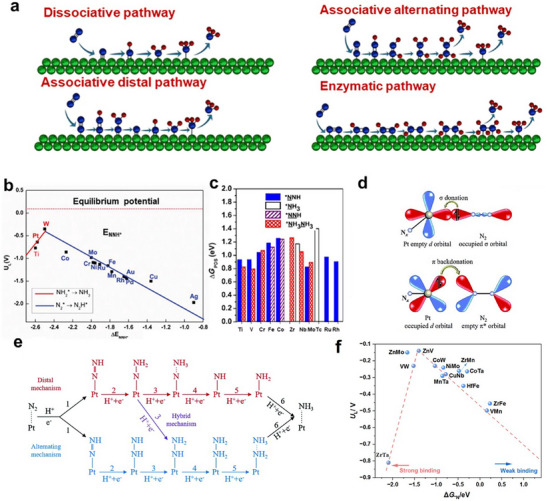
The possible NRR reaction pathways on catalyst surfaces: a) direct dissociative pathway, and enzymatic associative pathways. Adapted with permission.^[^ ^291^ ^]^ Copyright 2021 John Wiley and Sons. b) The computed NRR limiting potential (U_L_) as a function of the adsorption energy of NNH* species (∆E_NNH*_). Reproduced with permission.^[^ ^286^ ^]^ Copyright 2018 John Wiley and Sons. c) Reaction‐free energy change at PDS (ΔG_PDS_) on M/g‐C_3_N_4_‐N_2_ complex. Filling columns represent the product at the PDS. d) Charge density difference for N_2_ adsorption on Pt/g‐C_3_N_4_. Red and blue regions represent charge accumulation and depletion, respectively. The isosurfaces value is 0.002 e Å^−3^. e) Simplified schematic of N_2_ bonding to Pt/g‐C_3_N_4_. (g) Schematic depiction of the three reaction mechanisms for the electrochemical NRR on Pt/g‐C_3_N_4_. Reproduced with permission.^[^ ^231^ ^]^ Copyright 2019 RSC. f) A volcano plot between the U_L_ and *N adsorption Gibbs free energy among the constructed DACs models. Reproduced with permission.^[^ ^290^ ^]^ Copyright 2025 RSC American Chemical Society.

However, experimental studies on SACs for NRR applications have rarely been reported. Using DFT computations, Chen et al.^[^ ^286^ ^]^ evaluated the NRR catalytic performance of a series of single metal atoms embedded in the structure of g‐C_3_N_4_. The electrochemical NRR involves six consecutive proton‐coupled electron transfer reactions and may proceed via two types of reaction mechanisms: the dissociative pathway, in which the N ≡ N triple bond splits into two individual N atoms before being hydrogenated, and the associative pathway, in which the N─N bond cleaves simultaneously with the release of NH_3_. (**Figure** **10**a,b). All possible intermediates, including N_2_H*, N_2_H_2_*, N_2_H_3_*, N_2_H_4_*, N*, NH*, NH_2_*, and the formation of NNH* and the final NH_2_ desorption or NH_3_ release, were considered as the potential‐determining steps (PDS).^[^ ^230^ ^]^ Among all the studied SACs@g‐C_3_N_4_ systems, W@g‐C_3_N_4_ was the most promising candidate for electrochemical NRR and shows remarkably high catalytic activity with a limiting potential of –0.35 V via an associative enzymatic pathway (Figure 10c). Meanwhile, the competitive hydrogen evolution reaction was effectively suppressed by the Heyrovsky step on W@g‐C_3_N_4_. The high NRR activity and selectivity of W@g‐C_3_N_4_ were attributed to: i) the electrical conductivity greatly enhanced upon W anchoring, ensuring efficient electron transfer during the reduction process; ii) the spin moment on the W atom can be transferred to the inert N_2_ molecule, thus accelerating its further activation and subsequent reactions; and iii) moderate adsorption strength with NRR intermediates improves the reaction dynamics by facilitating and triggering the subsequent reduction pathways after each step.

Moreover, the DFT evaluation of g‐C_3_N_4_ supported transition metal single atoms as electrocatalysts for NRR was also conducted, which found that Mo@g‐C_3_N_4_ showed the highest catalytic activity for NRR in the standing‐on adsorbed N_2_ cases, while for lying‐on adsorbed N_2_ cases, V@g‐C_3_N_4_ exhibited better NRR performance.^[^ ^287^ ^]^ Yin et al.^[^ ^231^ ^]^ revealed that isolated Pt atoms and g‐C_3_N_4_ in the Pt@g‐C_3_N_4_ system work in concert to stabilize *NNH and destabilize the *NH_2_ intermediate, thereby synergistically offering highly active sites for electrochemical NH_3_ production. Furthermore, Pt@g‐C_3_N_4_ exhibited predominant adsorption behavior toward N_2_ (Figure 10d,e), over that of H atoms, which greatly hinders the hydrogen evolution process and improves the selectivity for NH_3_. Besides, the SACs@g‐C_3_N_4_ with different C/N ratios (e.g., g‐C_2_N, g‐CN, g‐C_3_N_4_, g‐C_4_N_3_, and g‐C_9_N_4_) have also been widely explored based on the deep neural network (DNN) classification model and the extreme gradient boosting (XGBoost) model, indicating that the bond length of N ≡ N and the number of outermost d electrons of TM (N_d_) play a critical role in the NRR activities and selectivity.^[^ ^288^, ^289^ ^]^

Thanks to the development of artificial intelligence and machine learning, Lei and co‐worker systematically investigated and designed 120 DACs doped with 15 transition metals (MM’@g‐C_6_N_6_ (M/M’ = Ti, V, Cr, Mn, Fe, Co, Ni, Cu, Zn, Zr, Nb, Mo, Hf, Ta, W) for predicting the electrocatalytic NRR activity and providing theoretical insights into the NRR electrocatalyst rational design.^[^ ^290^ ^]^ To efficiently screen the designed DACs for MM’@g‐C_6_N_6_, a 3+1 high‐throughput screening strategy was adopted to evaluate the NRR activities. Before evaluating the adsorption stability of N_2_ on the MM’@g‐C_6_N_6_ surface, the thermodynamic stability of DACs was preliminarily calculated. And, as the potential rate‐determining steps, the first hydrogenation step of N_2_ (*N_2_+H→*N_2_H) and the last hydrogenation step (*NH_2_+H→*NH_3_ were then investigated. Finally, the limiting potential (U_L_) of the NRR reaction was compared with the U_L_ value of HER (U_L_(NRR)−U_L_(HER)>0) to ensure their NRR selectivity. Theory analysis demonstrated that both ZnV@g‐C_6_N_6_ and ZnMo@g‐C_6_N_6_ was consider as the most potential structures for the high electrocatalytic nitrogen reduction activities, which displayed a more efficient d−π* orbital hybridization between the doped TM centers and *N_2_, the doped TM donates electrons to the antibonding orbitals of N_2_ combined within the lone pair of N_2_ occupy the d orbitals of TM atoms (Figure 10f). These theoretical predictions not only confirm the promising electrocatalytic performance of SACs@g‐C_3_N_4_ toward NRR but also establish a fundamental framework for understanding the structure‐activity relationships at the atomic level. The insights gained from these computational studies, particularly regarding the critical role of metal‐center electronic structure in N_2_ activation and intermediate stabilization, provide invaluable guidance for the rational design of next‐generation SACs.

#### 4.1.5 Other Electrochemical Reactions

The electrocatalytic oxidation of small organic molecules, such as methanol, formic acid, and ethanol, is another important branch in developing high‐efficiency and low‐cost clean energy conversion technologies, enabling the construction of types of liquid fuel cells, such as direct methanol fuel cells, direct formic acid fuel cells, and direct ethanol fuel cells.^[^ ^292^, ^293^ ^]^ Increasing efforts are being devoted to precisely controlling the structure and size of electrocatalysts to minimize the use of noble metals and promote catalytic activity. Hence, the fabrication of SACs seems to be a promising approach, inspired by the above design strategies for electrocatalysis.

**Figure 11:**
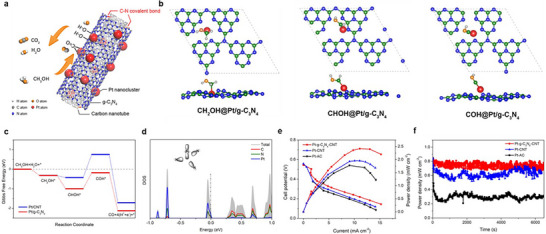
a) Schematic diagram of the methanol electrocatalytic processes on the Pt‐g‐C_3_N_4_‐CNT catalyst. b) Top and side views of atomic configurations of the intermediate states during methanol electrooxidation. Green, blue, gold, white, and red represent C, N, O, H, and Pt atoms, respectively. c)Free energy diagram of the methanol electrooxidation on Pt/g‐C_3_N_4_ and Pt/CNT. d) pDOS plots of Pt/g‐C_3_N_4_. e) Steady‐state polarization curves, power‐density curves, and f) discharge curves at 0.35 V of different materials as anode catalysts with 1 M methanol at 60 °C. Reproduced with permission.^[^ ^297^ ^]^ Reproduced with permission.^[^ ^299^ ^]^ Copyright 2019, American Chemical Society.

However, for methanol oxidation, the reaction follows a dual‐path mechanism. One indirect path proceeds through the formation of CO, where the “poison” of CO occupies the Pt catalytic sites and subsequently converts by reaction with adsorbed H_2_O or OH into CO_2_; the other direct path goes by way of formaldehyde and formic acid to CO_2_.^[^ ^294^, ^295^ ^]^ Mitani and co‐workers^[^ ^296^ ^]^ exhaustively studied the size effects on Pt catalysts for the methanol oxidation reaction and found that single Pt atoms have nearly no activity compared with Pt nanoparticles. This result is reasonable because methanol electrooxidation requires at least 3 Pt atoms to serve as active sites according to the proposed mechanism. Therefore, we recently reported a sub‐nanometric Pt catalytic system achieved by g‐C_3_N_4_ trapping for highly active methanol electrooxidation (**Figure** **11**a).^[^ ^297^ ^]^ DFT calculations were used to evaluate the effects of g‐C_3_N_4_ on the catalytic mechanism of the Pt atom on methanol oxidation. As shown in Figure 11b,c, all the reaction steps were thermodynamically downhill, except for the rate‐determining step of the formation of COH*. The g‐C_3_N_4_ as a support significantly improved the reactivity of the Pt catalysts by strengthening its binding with the intermediates during the methanol electrooxidation process, particularly for the rate‐determining step, and energetically reducing the energy barriers in the multistep reaction pathways. Another important role of g‐C_3_N_4_ is the facilitation of water dissociation to form OH* at a lower potential, which efficiently promotes the elimination of CO* poisoning and the release of active catalytic sites. Further study on the partial density of states (pDOS) reveals a high energy level of the d‐band position on Pt/g‐C_3_N_4_, again proving the enhanced adsorption behaviors for the key reaction intermediates (Figure 11d). As a result, Pt/g‐C_3_N_4_ exhibited excellent electrocatalytic performance, including high power density and reliable long‐term discharge stability, as an anode material for applications in direct methanol fuel cells (Figure 11e,f).

In addition, Chen and co‐workers^[^ ^133^, ^135^ ^]^ reported a facile refluxing method for incorporating ruthenium ions into the molecular skeletons of g‐C_3_N_4_. The formation of Ru‐N coordination bonds was confirmed by XPS analysis owing to the strong affinity of Ru ions to the pyridinic nitrogen in the structure of g‐C_3_N_4_. When working as an electrocatalyst for the hydrogen evolution reaction, Ru@g‐C_3_N_4_ shows greatly enhanced electrocatalytic performance in terms of overpotential, Tafel slope, and exchange current density. DFT calculations revealed that the Ru‐N active centers effectively facilitated the adsorption of hydrogen and positively shifted the conduction band of the Ru@g‐C_3_N_4_ hybrid, leading to a significantly lower energy barrier for hydrogen evolution. Besides, Zhu et al. further confirmed the potential of transition metals anchored in the g‐C_3_N_4_ for hydrogen production, fabricated the Co‐g‐C_3_N_4_/rGO SACs (Co‐CNG) with 20% of Co‐N and 80% of Co‐3N anchored on the g‐C_3_N_4_, condensed from the melamine.^[^ ^214^ ^]^ The abundant pyridinic nitrogen species incorporated into the g‐C_3_N_4_ structure of rGO provide ample anchoring sites for the atomic‐level dispersion of Ni species, which displayed even comparable HER performance (10 mA cm^−2^ at ≈47 mV) with commercial Pt/C. Theoretical analysis confirmed the downward shift of the d‐band center and the reduction of the HER free energy barrier in the Co–N model responsible for the excellent performance of the synthesized Co‐CNG catalyst. Zhao et al.^[^ ^136^ ^]^ investigated a single‐site Pt@g‐C_3_N_4_ electrocatalyst for application in Li‐O_2_ batteries. The as‐obtained Pt@g‐C_3_N_4_ exhibited excellent electrocatalytic activity, including high discharge specific capacities and durable stability. The correlation between the experimental and computational results proves that this high activity originates from the synergistic effects between the isolated Pt atom and holey ultrathin g‐C_3_N_4_, which provides a local built‐in electric field to the stable Pt atoms and accelerates efficient interfacial mass transfer. Wang et al.^[^ ^298^ ^]^ studied single cobalt atoms embedded in g‐C_3_N_4_ for CO oxidation using DFT calculations. For the CO oxidation process via various possible reaction mechanisms, Co@g‐C_3_N_4_ showed significantly reduced energy barriers for the rate‐determining steps and no CO poisoning. Combined with the excellent ORR/OER activities of Co@g‐C_3_N_4_, as demonstrated by both the experimental and theoretical analysis in numerous publications, Co@g‐C_3_N_4_ has potential as a multifunctional catalyst for fuel cell applications.

Beyond the well‐established HER and OER applications, g‐C_3_N_4_ demonstrates remarkable potential in electrocatalyzing various small molecule reactions. The unique nitrogen‐rich architecture enables the effective stabilization of single metal atoms through metal–N coordination, creating highly active centers for the oxidation of formic acid and ethanol. More importantly, the electronic modulation capability of g‐C_3_N_4_ optimizes the adsorption/desorption behavior of key intermediates in these multi‐step reactions. The material's ability to facilitate OH* generation at lower potentials proves particularly valuable in preventing catalyst poisoning by CO‐like species during formic acid oxidation. Furthermore, the predictable coordination environment of g‐C_3_N_4_ allows for rational design of catalytic sites tailored to specific reaction pathways, potentially enabling simultaneous optimization of activity and selectivity in complex organic molecule electrooxidation, a significant advantage over conventional carbon supports.

Among the various applications, the NRR benefits the most from the unique M–N coordination on g‐C_3_N_4_. First, the inherent nitrogen richness of the g‐C_3_N_4_ matrix grants its M–N_x_ sites a natural affinity for N_2_ molecules, enabling efficient adsorption and activation of the notoriously stable N ≡ N triple bond, which is the rate‐determining step. Second, the specific coordination environment effectively modulates the electronic structure of the active metal center, weakening the adsorption of hydrogen intermediates (H*) and thus strongly suppressing the unfavorable HER, which significantly enhanced Faradaic efficiency for NH_3_ generation. Finally, the well‐defined atomic “pocket” created by the M–N sites stabilizes the various intermediates generated during the multi‐step proton‐electron transfer process of NRR, thereby guiding the reaction pathway. Consequently, the M–N coordination in g‐C_3_N_4_ directly addresses the fundamental challenges of NRR, activation, selectivity, and pathway management, making its advantages more critical and less easily substituted than in other electrocatalytic reactions.

### 4.2 Photocatalysis

Ever since the pioneering work of Fujishina and Honda on photoelectrochemical water splitting with a TiO_2_ electrode,^[^ ^299^ ^]^ solar energy conversion and utilization have become one of the most intensive research areas, aiming to resolve the global crisis of energy shortage and environmental issues.^[^ ^300^, ^301^ ^]^ Artificial photosynthesis affords a straightforward approach toward renewable solar‐to‐chemical energy conversion, and the heart of these systems is a semiconductor material that can absorb photons, transform them into excited electronic states, and drive the solar fuel production reactions. g‐C_3_N_4_, a typical carbon‐based semiconductor, has emerged with inestimable superiority because of its physicochemical stability, unique electronic band structure, tunable molecular properties, and facile synthesis. The incorporation of SACs with g‐C_3_N_4_ has proven to be an efficient way to further adjust the bandgap of g‐C_3_N_4_, accelerating charge transfer and strengthening the active sites; hence, tremendous efforts have been devoted. In this section, we present a few selected examples of the application of SACs@g‐C_3_N_4_ in key solar energy conversion reactions, such as photocatalytic H_2_ production, photocatalytic CO_2_ reduction, photocatalytic decomposition, and mineralization of organic pollutants, to demonstrate the capability and great potential of SACs@g‐C_3_N_4_ for highly efficient photocatalytic systems. Furthermore, the relationship between the structure and performance of SACs@g‐C_3_N_4_ along with the photocatalytic mechanism is also discussed.

#### 4.2.1 Photocatalytic H_2_ Production

Photocatalytic water splitting into H_2_ fuel generally involves three key steps: i) light absorption of the semiconductors to generate photoexcited electron and hole pairs, ii) charge separation followed by the transfer of these photoexcited carriers to the semiconductor/solution interfaces, and iii) surface catalytic reactions driven from these carriers with various compounds (e.g., H_2_O).^[^ ^302^, ^303^, ^304^ ^]^ Note that upon the electron and hole pairs being photogenerated, the recombination phenomenon may occur during the transfer process, which significantly decreases the photocatalytic performance. Desired materials should fulfil stringent requirements, including good light‐harvesting properties, good charge separation efficiency, and suitable energetic positions for water reduction and oxidation.

The g‐C_3_N_4_ is a promising metal‐free semiconductor candidate with a bandgap of ≈2.7 eV for light absorption at λ < 460 nm, and its highest occupied molecular orbital (HOMO) and lowest unoccupied molecular orbital (LUMO) positions have been proven to be suitable for driving photocatalytic water splitting. However, although g‐C_3_N_4_ materials are utilized in photocatalytic water splitting, several factors still hinder their practical application, such as poor electronic conductivity, short electron diffusion length, and sluggish hole transfer kinetics. Loading co‐catalysts is considered to be an effective way to address these intractable challenges and to maximize the atomic efficiency and activity, SACs stand out as the most promising options, as they not only serve as co‐catalysts to suppress charge carrier recombination and enhance the interfacial reaction dynamics but also to optimize the electronic structure of g‐C_3_N_4_ and provide atomic‐level insights into the intrinsic active sites and catalytic pathways. Extensive effects have been devoted to various metals, including Pt,^[^ ^137^, ^305^, ^306^ ^]^ Pd,^[^ ^131^, ^163^ ^]^ and other non‐precious transition metals (Fe,^[^ ^209^, ^307^ ^]^ Co,^[^ ^208^, ^308^ ^]^ Cu,^[^ ^309^, ^310^ ^]^ Zn,^[^ ^143^ ^]^ Na,^[^ ^180^, ^196^ ^]^ and Al^[^ ^311^ ^]^), all of which demonstrate a significant impact on the enhancement of the photocatalytic H_2_ production (**Table** **2**).

**Table 2:** Selected highly efficient SAC@g‐C_3_N_4_ photocatalysts for hydrogen production.

Samples	Cocatalyst	Sacrificial agent	Light source	Wavelength of cut‐off filter [nm]	H_2_ production µmol g^−1^ h^−1^	AQE (%)	TOF [h^−1^]	Refs.
g‐C_3_N_4_‐Pt^2+^	–	TEOA	–	400	605	–	–	[109]
PtSA‐CN620	–	TEOA	300 W Xe lamp	420	174 500	0.544% (420 nm)	–	[127]
SA‐Pt/g‐C_3_N_4_‐8.7	–	TEOA	300 W Xe lamp	420	22 650	22.5% (420 nm)	–	[137]
Pt‐CN	–	TEOA	30‐W LED lamp	–	3420	0.84% (520 nm)	–	[138]
Pt‐CN	–	TEOA	300 W Xe lamp	–	6360	–	775	[84]
Pt‐SA/CN	–	Methanol	300 W Xe lamp	300	1403.3	–	250	[158]
Pt_0.1_‐CN	–	Methanol	300 W Xe lamp	420	118.5	–	–	[159]
0.1 Pd/C_3_N_4_	–	TEOA	300 W Xe lamp	400	728	–	–	[131]
Pd/g‐CN	–	TEOA	350 W Xe lamp	–	6688	≈4% (420 nm)	417	[157]
Co_1_/PCN	–	TEOA	300 W Xe lamp	300	216	3.02% (450 nm)	1.2	[97]
Co_1_‐phosphide/PCN	–	None	300 W Xe lamp	–	410.3	2.2% (500 nm)	–	[126]
Co@g‐C_3_N_4_	Pt	TEOA	300 W Xe lamp	420	2481	5.04% (420 nm)	–	[208]
Fe@g‐C_3_N_4_	Pt	TEOA	300 W Xe lamp	420	3390	6.89% (420 nm)	–	[209]
Fe‐g‐CN	Pt	TEA	a white light LED with Eosin Y	–	16 200	0.8%	–	[307]
Zn/g‐C_3_N_4_	Pt	Methanol	200 W Xe lamp	420	297.5	3.2% (420 nm)	–	[143]
Na_x_‐CNNTs	Pt	TEOA	300 W Xe lamp	420	7150	1.8% (420 nm)	–	[180]

**Figure 12:**
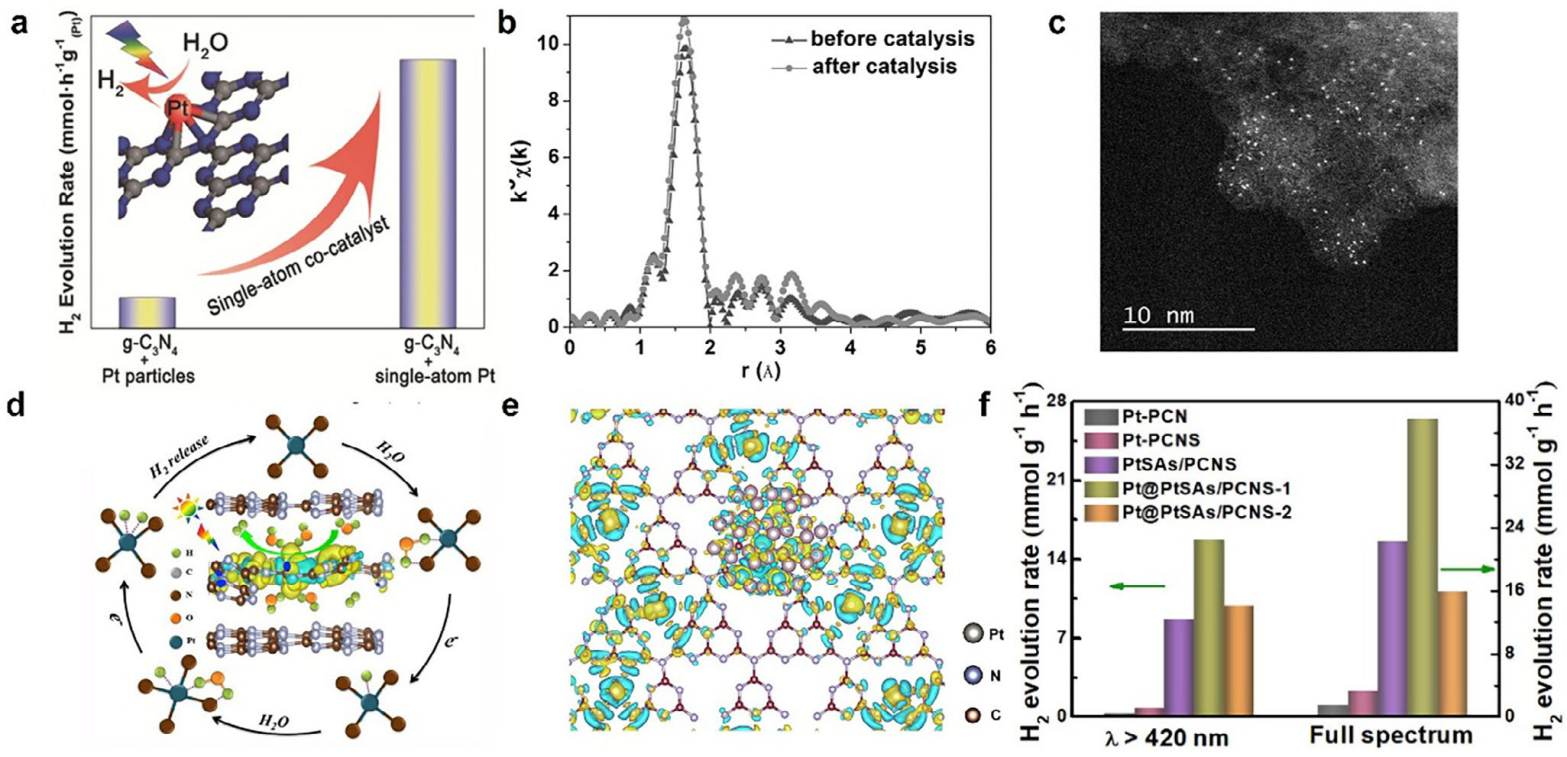
a) Photocatalytic activity comparison of g‐C_3_N_4_ with Pt nanoparticles and single‐atom Pt as the co‐catalyst, the inset is the schematic models of Pt‐CN. b) Comparison of FT‐EXAFS curves of the Pt‐CN before and after the photocatalytic reaction. c) HAADF‐STEM image of Pt‐CN after the photocatalytic reaction. Reproduced with permission.^[^ ^84^ ^]^ Copyright 2016, John Wiley and Sons. d) Illustration of the proposed mechanism for photocatalytic H_2_ production. Reproduced with permission.^[^ ^137^ ^]^ Copyright 2020, Elsevier. e) Difference charge density analysis of Pt@PtSAs/PCNS. f) The photocatalytic H_2_ evolution rate between Pt@PtSAs/PCNS and reference samples under visible light and full spectrum. Reproduced with permission.^[^ ^305^ ^]^ Copyright 2022, Elsevier.

For example, Xie and co‐workers^[^ ^84^ ^]^ reported a highly efficient photocatalytic system constructed using isolated single Pt atoms and g‐C_3_N_4_ nanosheets (Pt‐CN) for H_2_ evolution. The sub‐nanoporosity in 2D g‐C_3_N_4_ endows the trapping of Pt species to promote the subsequent uniform formation of Pt‐N/C bonds with the tri‐s‐triazine units in g‐C_3_N_4_, leading to the achievement of high dispersion and stability of single‐atom Pt. Functioning as a co‐catalyst, the single‐atom Pt shows a significantly enhanced photocatalytic H_2_ evolution performance, nearly ten times higher than that of Pt nanoparticles and 50 times that of bare g‐C_3_N_4_ (**Figure** **12**a). Ultrafast transient absorption analysis revealed that the boosted performance was attributed to the intrinsic change in the surface trap states of g‐C_3_N_4_ induced by the incorporation of isolated single Pt atoms, which remarkably strengthened the lifetime of the photoexcited electrons and thus provided more possibilities for them to participate in the H^+^ evolution reaction. Furthermore, the only sharp peak corresponding to the Pt–N bond in FT‐EXAFS (Figure 12b) and the clear observation of isolated single dots without aggregation in HAADF‐STEM (Figure 12c) clearly confirmed the excellent structural stability of single‐atom Pt co‐catalysts on g‐C_3_N_4_ even after the harsh photocatalytic reaction. Zeng et al.^[^ ^137^ ^]^ confined single Pt atoms via the interlayer subnanospace of layered g‐C_3_N_4_ and demonstrated that the location of Pt atoms is another key factor in improving the photocatalytic performance. It was revealed that the interlayer interactions between Pt and g‐C_3_N_4_ effectively altered the electronic structure of g‐C_3_N_4_, delocalizing the charge density of the isolated Pt atoms along the vertical direction, accelerating proton adsorption, and further decreasing the energy barriers for photocatalytic H_2_ production (Figure 12d). The obtained photocatalysts exhibited highly efficient photocatalytic H_2_ production activity with an ultrahigh apparent quantum yield. Yang and teammates constructed a strong electronic metal‐support interaction (EMSI) between the nucleus‐like Pt NPs and diffuse PtSAs on the g‐C_3_N_4_ system (Pt@PtSAs/PCNS) to improve the photocatalytic H_2_ production performance under visible light and full spectrum.^[^ ^305^ ^]^ Based on the built model from the XAS analysis, the DFT calculations on the structural variations of PCNS demonstrated Pt@PtSAs/PCNS system exhibited a higher stretching degree compared with PtSAs/PCNS, which greatly induced the Pt–N constriction and electron delocalization within the PtSAs moiety, manifesting the enhanced EMSI at the PtSAs‐PCNS interface (Figure 12e). Electronic structure analysis revealed that in the Pt@PtSAs/PCNS system, electrons transfer from PtSAs to PtNPs through the PCNS interface. This electron donation confirms the strong electronic coupling between Pt NPs and PtSAs, which likely facilitates enhanced photoexcited electron transfer from PCNS to the Pt@PtSAs active sites. Compared with the Pt‐PSNS, obtained through in situ deposition of 3% Pt onto pure PCNS, and PtSAs/PCNS, the Pt@PtSAs/PCNS‐1 exhibits excellent photocatalytic activity with an evolution rate reaching 15.75 mmol g^−1^ h^−1^ using triethanolamine as the sacrificial electron donor. The Pt@PtSAs/PCNS‐1 also delivered the highest photocatalytic H_2_ evolution performance among these samples, with the H_2_ evolution rate of 37.8 mmol g^−1^ h^−1^ and the TON of 423 h^−1^, even under full spectrum with Xe lamp irradiation (Figure 12f).

**Figure 13:**
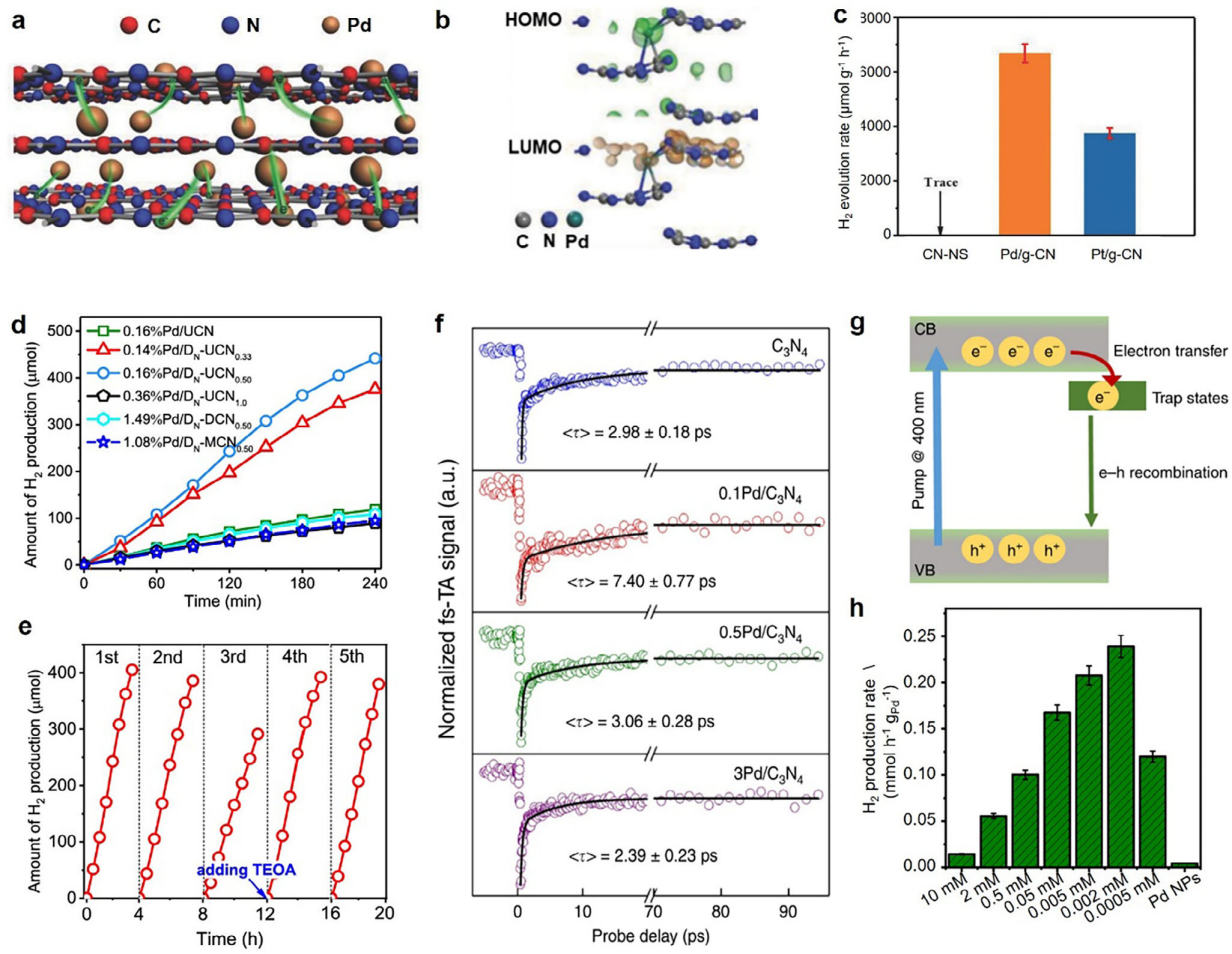
a) Conceptual illustration of Pd/g‐CN hybrid with the interlayer intercalation and surface anchor of Pd atoms, b) The HOMO/LUMO calculation of Pd/g‐CN. c) Photocatalytic H_2_ production activities between Pd/g‐CN and reference samples. Reproduced with permission.^[^ ^157^ ^]^ Copyright 2018, John Wiley and Sons. d) Photocatalytic H_2_ evolution activity of Pd/g‐C_3_N_4_ with different Pd mass loading. e) Photocatalytic H_2_ evolution stability over 0.16%Pd/DN‐UCN_0.50_ of the full solar light spectrum. Reproduced with permission.^[^ ^163^ ^]^ Copyright 2022, American Chemical Society. f) Representative femtosecond‐TA kinetics pump at 400 nm, probed at 520 nm for C_3_N_4_ and different loading Pd/C_3_N_4_ samples. g) A proposed mechanism underlying the involved photophysical processes. Reproduced with permission.^[^ ^131^ ^]^ Copyright 2019, Springer Nature. h) Normalized H_2_ evolution rates for different concentrations of Pd SAs. Reproduced with permission.^[^ ^313^ ^]^ Copyright 2025, Royal Society of Chemistry.

As described in the research conducted by Jiang and co‐workers, an enhanced photocatalytic H_2_ production activity was achieved by the constructed palladium‐doped g‐C_3_N_4_, originating from the effective alteration of the electron excitation manner and accelerating dynamic kinetics.^[^ ^312^ ^]^ The introduction of Pd species into the framework of g‐C_3_N_4_ to prepare photo‐SACs has shown excellent potential in photocatalytic hydrogen production and attracted widespread attention. For instance, Cao et al.^[^ ^157^ ^]^ reported an atomic‐engineering strategy to anchor isolated Pd atoms on both the bridge sites of adjacent layers and the surface sites of g‐C_3_N_4_ (**Figure** **13**a). Upon irradiation, this featured structure can provide directional charge‐transfer channels for simultaneously achieving vertical transport and in‐plane migration of the photoexcited electrons to target the active sites for photocatalytic H_2_ evolution. DFT simulations corroborated the photoexcited charge carriers’ transfer behavior through the HOMO‐LUMO structure, which verified the formation of a built‐in electric field within the Pd/g‐CN (Figure 13b). This built‐in electric field played a critical role in increasing the charge separation efficiency and further inducing directional transport; thus, the Pd/g‐CN photocatalyst showed highly efficient and stable photocatalytic H_2_ production performance, which is far more competitive than the optimized Pt/g‐CN benchmark (Figure 13c). Similarly, Ren et al. synthesized interlayer Pd SACs loaded on the cyano‐riced g‐C_3_N_4_, through coordination with the cyano group and sp^2^‐hybridized N atoms in the adjacent layer, and the photocatalytic H_2_ production performance was enhanced. Benefited from the midgap state induction from the synergistic interactions of the cyano groups and Pd–N coordination, resulting in boosted photoexcited electrons and maximal Pd atom utilization efficiency, the apparent quantum yield values of the optimal 0.16%Pd/DN‐UCN_0.50_ catalyst with a unique Pd–N_3_ coordination environment could be significantly improved (Figure 13d). The photocatalytic measurements show satisfactory stability after five continuous catalytic cycles, demonstrating the excellent stability of the interlayer‐bridged Pd–N_3_ structure (Figure 13e). Liu et al.^[^ ^131^ ^]^ fabricated single Pd atoms on g‐C_3_N_4_ catalysts to demonstrate their role in catalyzing H_2_ production reactions. The femtosecond‐TA analysis in Figure 13f shows that Pd/g‐C_3_N_4_ with a low metal loading of 0.1 wt.% exhibits the longest mean recovery lifetime, indicating a faster charge transfer process and efficient charge separation in comparison with other catalysts. Consistently, 0.1Pd/g‐C_3_N_4_ showed a remarkably high H_2_ production rate of up to 728 µmol g^−1^ h^−1^ under visible light irradiation, which was superior to that of its pristine counterpart. This is derived from the intrinsic optimization of the near‐band‐edge electron trap states of g‐C_3_N_4_ after the introduction of isolated Pd single atoms, which benefited the longer‐lived photoexcited electrons reaching the interfaces and participating in the H^+^ reduction reaction (Figure 13g). Further DFT calculations clarified that the HOMO potentials of g‐C_3_N_4_ units coordinated with atomic Pd dramatically decreased, whereas those without interaction with Pd atoms were close to those of bare g‐C_3_N_4_. More recently, Jeyalakshmi et al. reported a “spontaneous deposition” approach, worked as a controllable uniform loading of Pd SACs to fabricate Pd SACs loaded onto g‐C_3_N_4_ substrates.^[^ ^313^ ^]^ Through adjusting the amount of deposition concentrations in Pd precursor, a range of Pd SAs/C_3_N_4_ with distinct Pt species mass loading were synthesized. Photocatalytic hydrogen production measurement demonstrated that the optimized Pd SAs/C_3_N_4_ with a low loading of 0.05 wt.% Pd SAs achieved a remarkable hydrogen production efficiency of 0.24 mmol h^−1^ mg_Pd_^−1^, displaying ≈50 times larger than that of Pd nanoparticles deposited on g‐C_3_N_4_ (Figure 13h). These excellent results could be attributed to the accelerated charge transfer from the g‐C_3_N_4_ to the Pd/g‐C_3_N_4_ units through the constructed coordination interaction of Pd SAs with the g‐C_3_N_4_.

**Figure 14:**
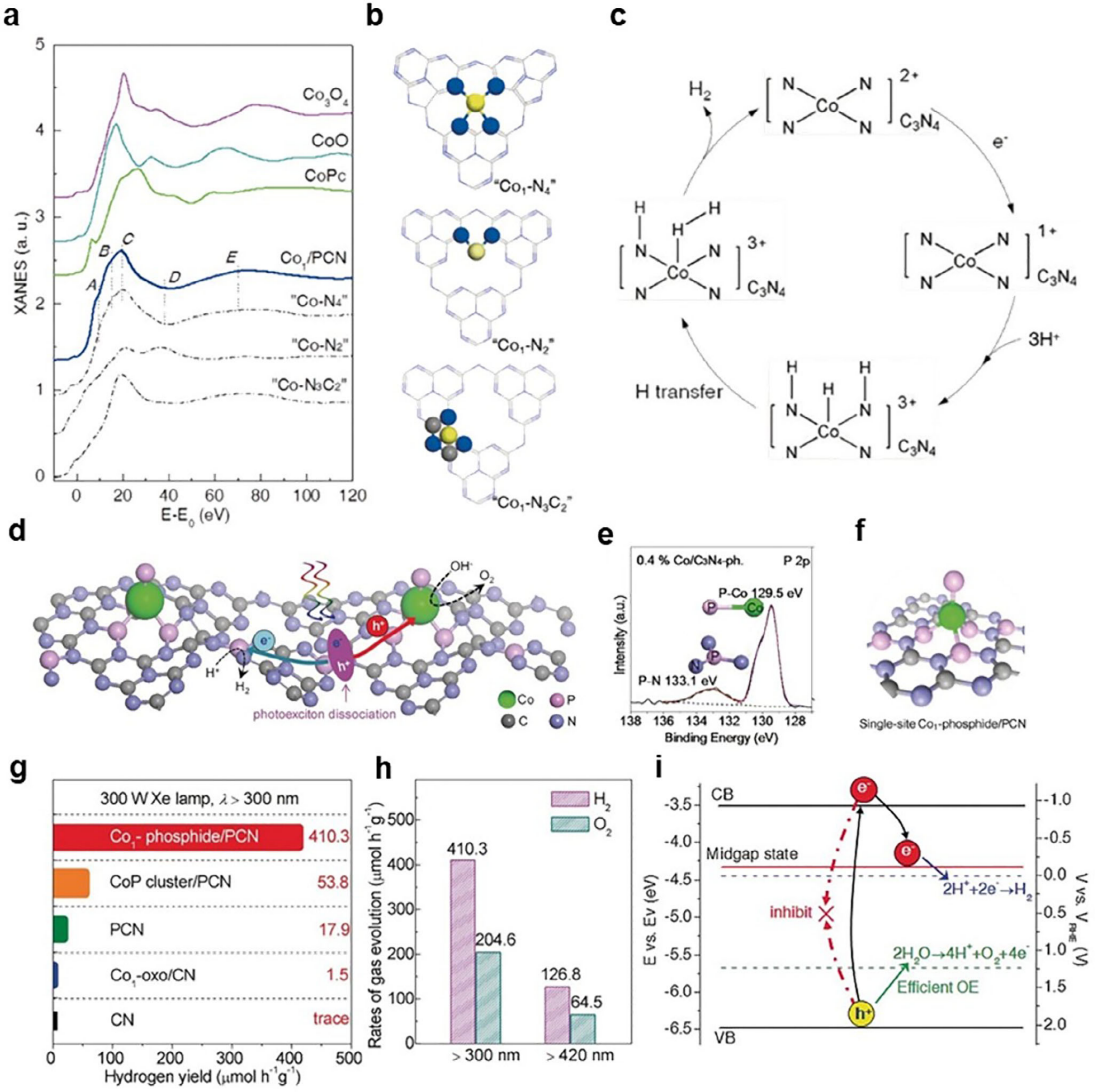
a) Co K‐edge XANES experimental spectra of Co_1_/PCN, CoPc, Co_3_O_4_, and CoO, and the calculated spectra for three representative model structures of Co_1_–N_4_, Co_1_–N_2_, and Co_1_–N_3_C_2_. b) Top view of the model structures of Co_1_–N_4_, Co_1_–N_2_, and Co_1_–N_3_C_2_. c) Proposed photocatalytic H_2_ evolution mechanism. Reproduced with permission.^[^ ^97^ ^]^ Copyright 2017, John Wiley and Sons. d) Schematic illustration of the solar‐driven overall water splitting on the Co_1_‐phosphide/PCN photocatalyst. e) High‐resolution P 2p XPS spectra of 0.4% Co/C_3_N_4_‐phosphidation sample. f) Schematic structural model for Co_1_‐phosphide/PCN. Green, pink, violet, and gray represent Co, P, N, and C atoms, respectively. g) H_2_ yield rates of Co_1_‐phosphide/PCN, CoP cluster/PCN, PCN, Co_1_‐oxo/C_3_N_4_, and g‐C_3_N_4_. h) Photocatalytic water‐splitting activities under simulated sunlight (λ > 300 nm) and visible (λ > 420 nm) light irradiation. i) Electronic band structure diagram for Co_1_‐phosphide/PCN photocatalyst. VB = valence band, CB = conduction band. Reproduced with permission.^[^ ^126^ ^]^ Copyright 2017, John Wiley and Sons.

Apart from these noble metals, various non‐precious transition metals have also been stabilized on g‐C_3_N_4_ for application in photocatalytic H_2_ production. However, the catalytic mechanisms in these nanosystems are still under investigation, mainly existing disputes regarding the roles of non‐precious transition metal SACs. Some research groups have reported that even though the incorporation of isolated single metals with g‐C_3_N_4_ can effectively optimize the electron/band structures, extend the visible‐light harvesting, and strengthen the feasibility of H_2_ activation, Pt cocatalysts are still needed to serve as accessible active sites for achieving highly efficient photocatalytic H_2_ production performance.^[^ ^208^, ^209^ ^]^ Whereas Cao et al.^[^ ^97^ ^]^ developed an atomically dispersed Co_1_–N_4_ composite as a prototypical photocatalyst without the assistance of noble cocatalysts for efficient H_2_ evolution. XANES simulations clarified that the atomically dispersed Co atoms were integrated into g‐C_3_N_4_ nanosheets via the Co_1_–N_4_ configuration in a void with four unsaturated N atoms in the presence of N vacancies (**Figure** **14**a,b). This Co_1_–N_4_ motif is involved as the active center following a homolytic‐like route, where two adsorbed H* stem from Co and N sites and the formation of H–H bond is activated by the redox process of Co, consequently resulting in a robust H_2_ evolution activity up to 10.8 µmol h^−1^ (Figure 14c). In the same group,^[^ ^126^ ^]^ they further demonstrated a single Co_1_–P_4_ geometry on g‐C_3_N_4_ nanosheets for overall solar‐driven water splitting (Figure 14d). Under the phosphatization effect, partial carbon atoms in the triazine units in g‐C_3_N_4_ were substituted by phosphorus atoms, and the isolated Co atoms were anchored on the P‐doped g‐C_3_N_4_ matrix by forming a Co_1_–P_4_ structure, as confirmed by P 2p XPS analysis (Figure 14e,f). The Co_1_–P_4_/PCN photocatalyst showed the best H_2_ production rate of 410.3 µmol h^−1^ g^−1^ under full spectrum irradiation (λ > 300 nm) and 126.8 µmol h^−1^ g^−1^ under visible light irradiation (λ > 420 nm) (Figure 14g,h). As illustrated in Figure 14i, Co_1_–P_4_ built a new mid‐gap state between the CB and VB, which not only broadens the visible light absorption but also functions as separation centers to trap photoinduced electrons from the CB, enabling efficient charge separation and transfer, prolonging the carrier lifetime, and boosting the water splitting activity.

In summary, SACs@g‐C_3_N_4_ photocatalysts are considered viable alternatives for H_2_ evolution under visible‐light irradiation. The M–N motifs can significantly strengthen the water molecular adsorption and activation behavior, along with optimizing the electronic structures of g‐C_3_N_4_. Noble‐metal SACs were comprehensively investigated and exhibited highly efficient H_2_ evolution activity, but this was not a good choice if considering a fully economical system for solar energy conversion. Thus, considerable effort should be devoted to exploiting non‐noble metal SACs on g‐C_3_N_4_ for visible‐light photocatalytic H_2_ production, which remains an ongoing challenge.

#### 4.2.2 Photocatalytic CO_2_ Reduction

By mimicking the natural photosynthetic process, photocatalytic reduction of CO_2_ into hydrocarbon fuels represents a clean, carbon‐neutral, and sustainable strategy to simultaneously address the increasing CO_2_ emissions and global energy shortage.^[^ ^314^, ^315^, ^316^ ^]^ As shown in Equations (5)—(11), CO_2_ reduction is a multi‐electron transfer process with various potential parallel reaction pathways, and different reduction products can be obtained, including CO, HCOOH, HCHO, CH_3_OH, and CH_4_.^[^ ^75^, ^317^, ^318^, ^319^ ^]^ Therefore, the development of robust catalysts with high activity and selectivity is the core mission for the efficient conversion of CO_2_ to fuels, considering the competitive reactions involved in this complicated process.

(5) $$CO_{2}+e^{-}\to C{O_{2}}^{-}\;E_{0}=-1.90V\left(\mathrm{vs}.\mathrm{NHEa}\mathrm{tpH}7\right)$$

(6) $$CO_{2}+2H^{+}+2e^{-}\to \mathrm{HCOOH}\;E_{0}=-0.61V\left(\mathrm{vs}.\mathrm{NHEa}\mathrm{tpH}7\right)$$

(7) $$CO_{2}+2H^{+}+2e^{-}\to \mathrm{CO}+H_{2}O\;E_{0}=-0.53V\left(\mathrm{vs}.\mathrm{NHEa}\mathrm{tpH}7\right)$$

(8) $$CO_{2}+4H^{+}+4e^{-}\to \mathrm{HCHO}+H_{2}O\;E_{0}=-0.48V\left(\mathrm{vs}.\mathrm{NHEa}\mathrm{tpH}7\right)$$

(9) $$\begin{array}{c} CO_{2}+6H^{+}+6e^{-}\to CH_{3}\mathrm{OH}+H_{2}O\;E_{0}=-0.38V\left(\mathrm{vs}.\mathrm{NHEa}\mathrm{tpH}7\right) \end{array}$$

(10) $$CO_{2}+8H^{+}+8e^{-}\to CH_{4}+H_{2}O\;E_{0}=-0.24V\left(\mathrm{vs}.\mathrm{NHEa}\mathrm{tpH}7\right)$$

(11) $$2H^{+}+2e^{-}\to \;H_{2}\;E_{0}=-0.41V\left(\mathrm{vs}.\mathrm{NHEa}\mathrm{tpH}7\right)$$

Recent advances in this area have demonstrated that g‐C_3_N_4_ is a promising candidate for driving photocatalytic CO_2_ reduction reactions.^[^ ^320^, ^321^, ^322^ ^]^ Antonietti and coworkers^[^ ^35^ ^]^ first revealed that the VB of g‐C_3_N_4_ is derived from nitrogen *p_z_* orbitals, and the CB predominantly consists of carbon *p_z_* orbitals, which are located at ≈–1.1 *V*_NHE_. To trigger a certain reaction, the energy potential of the photoinduced electrons in the CB should be greater than the required reaction potential. However, the high reaction potential for the dissociation of CO_2_ to CO_2_^–^ of –1.90 *V*_NHE_ eliminated almost all the semiconductor photocatalysts. Thus, this process is very unlikely to occur via the photoreduction route.^[^ ^323^, ^324^ ^]^ For other CO_2_ reduction routes, g‐C_3_N_4_ has the ability to drive thermodynamically. Dong et al.^[^ ^325^ ^]^ reported that g‐C_3_N_4_ synthesized from melamine hydrochloride can effectively convert CO_2_ into CO in the presence of water vapor without any cocatalysts and showed fascinating porous structure‐dependent reactivity for photoreduction under visible light irradiation. Mao et al.^[^ ^326^ ^]^ compared two types of g‐C_3_N_4_ pyrolyzed from urea and melamine as photocatalysts for CO_2_ reduction under visible light. The product derived from urea exhibited higher activities and selectivity for the formation of both CH_3_OH and C_2_H_5_OH, owing to the mesoporous flake‐like structure and large specific surface areas.

The main concerns of pristine g‐C_3_N_4_ for photocatalytic CO_2_ reduction are the fast recombination of photoexcited charge carriers, slow carrier transfer kinetics, and limited absorption of visible light, which are the same obstacles as the previously discussed photocatalytic H_2_ production. Therefore, tremendous efforts have been devoted to structural manipulation for enhanced photocatalytic performance, such as heteroatom doping, coordination interactions, covalent and noncovalent functionalization, copolymerization, and modulation of intralayer hydrogen bonding. Among them, doping and metal coordination are effective approaches for introducing active sites into the g‐C_3_N_4_ framework to adjust its electronic structure and energy band configuration. Yu and coworkers^[^ ^327^ ^]^ fabricated sulfur‐doped g‐C_3_N_4_ as an advanced metal‐free catalyst for photocatalytic reduction of CO_2_. Sulfur‐doped g‐C_3_N_4_ showed broad light absorption up to 475 nm and a narrow bandgap of 2.63 eV. DFT calculations revealed that the doping sites constructed an impurity state in the bandgap structure, whereby the photoexcited electrons could be effectively transferred from the impurity state to the CB or from the VB to the impurity state, restricting the recombination and leading to a greatly enhanced photoactivity toward CO_2_ reduction with the selective formation of CH_3_OH. In addition, other non‐metal elements were also investigated to incorporate into the framework of g‐C_3_N_4_, such as boron,^[^ ^328^, ^329^ ^]^ oxygen,^[^ ^330^ ^]^ and phosphorus,^[^ ^71^, ^331^, ^332^, ^333^ ^]^ all of which have been proven to be crucial for improving the CO_2_ reduction activity via participating and modifying the photocatalysis mechanism.

**Figure 15:**
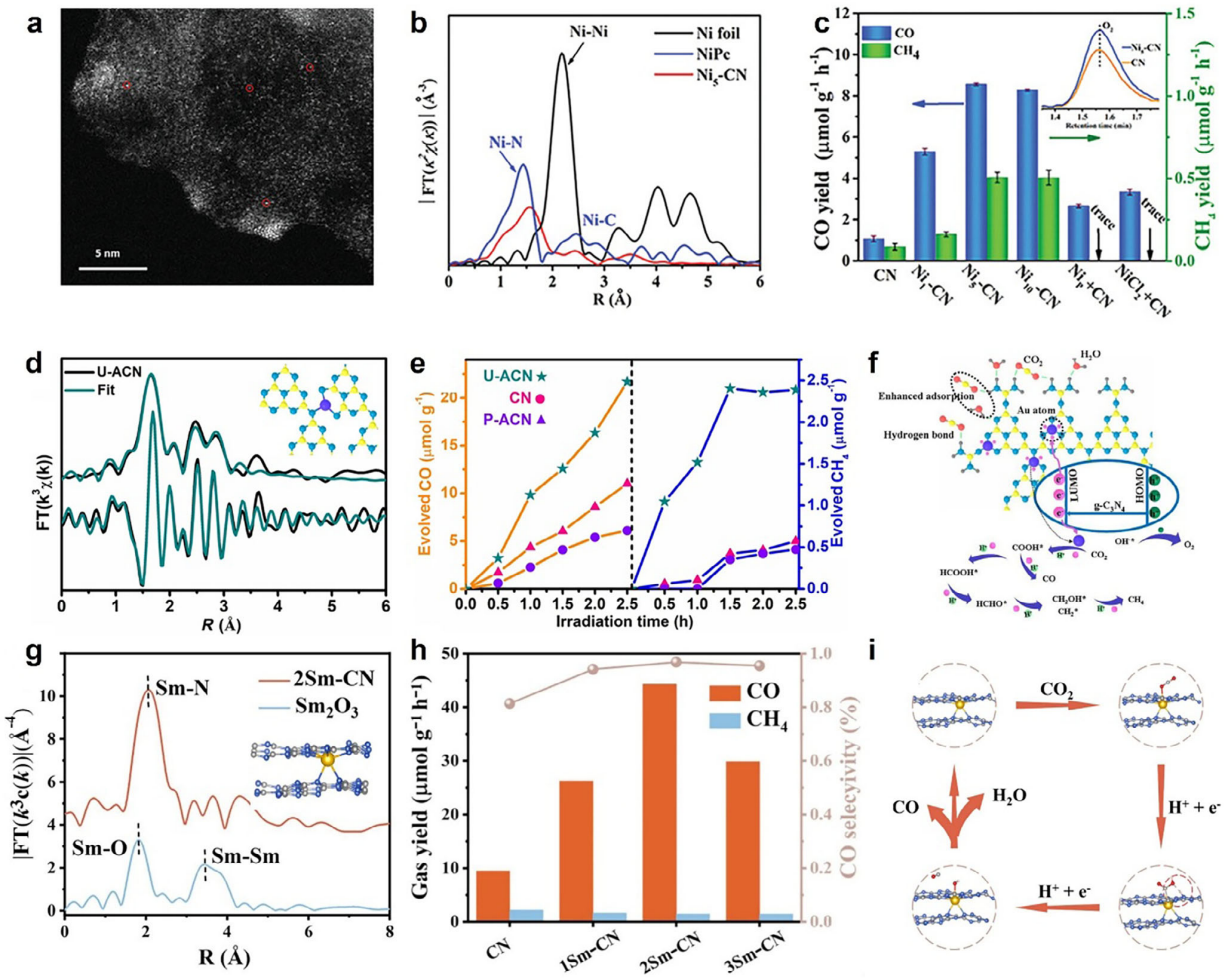
a) AC HAADF–STEM image and b) FT‐EXAFS spectra of the Ni_5_‐CN photocatalyst. c) Photocatalytic products of CO_2_ reduction over Ni_5_‐CN under visible light illumination for 1 h among different samples. Reproduced with permission.^[^ ^346^ ^]^ Copyright 2020, John Wiley and Sons. d) EXAFS fitting curve of the U‐ACN. e) CH_4_ (right blue) and CO (left in yellow) yield curves of U‐CAN, CN, and P‐CAN versus reaction time under visible‐light irradiation. f) Proposed illustration of electron distribution and the CO_2_ process of U‐ACN under visible‐light irradiation. Reproduced with permission.^[^ ^139^ ^]^ Copyright 2020, John Wiley and Sons. g) Sm L_3_‐edge k^3^‐weighted Fourier transform between 2SmCN and Sm_2_O_3_. h) Photocatalytic performance over different samples for the CO_2_ reduction reaction. i) Schematic diagram of photocatalytic reduction of CO_2_ over 2Sm‐CN. Reproduced with permission.^[^ ^322^ ^]^ Copyright 2025, Elsevier.

The integration of SACs on g‐C_3_N_4_ has recently drawn intensive attention for achieving high activity and selectivity towards photocatalytic CO_2_ reduction. It was found that the atomically dispersed SACs can not only optimize the electronic band structure of g‐C_3_N_4_ but also, more importantly, provide accessible active centers to strengthen CO_2_ adsorption, facilitate interfacial reaction dynamics, and reduce energy barriers. To date, a variety of atomically dispersed SACs have been successfully synthesized on g‐C_3_N_4_ from the non‐precious metals K,^[^ ^334^, ^335^, ^336^ ^]^ Co,^[^ ^120^, ^321^, ^337^, ^338^ ^]^ Cu,^[^ ^339^, ^340^ ^]^ and Mo^[^ ^341^, ^342^, ^343^ ^]^ to precious Au,^[^ ^139^ ^]^ and even rare metals,^[^ ^195^, ^322^, ^344^, ^345^ ^]^ as listed in **Table** **3**. For example, Huang et al.^[^ ^120^ ^]^ activated single Co^2+^ sites on g‐C_3_N_4_ using a simple deposition method as a photosensitizer for photocatalytic CO_2_ reduction. The effect of cobalt loading revealed that the amount of CO generated during CO_2_ reduction increased linearly with a maximum loading of 0.128 µmolmg^−1^, which showed a quantum yield of 0.40 %. The turnover numbers (TONs) were examined using the optimum Co^2+^@g‐C_3_N_4_ photocatalysts in the absence of triethylamine and showed a high value of over 200 for CO production after 24 h. Cheng et al. developed an unsaturated edge confinement method to synthesize porous few‐layer g‐C_3_N_4_ (namely, Ni_5_‐CN).^[^ ^346^ ^]^ This as‐obtained Ni_5_‐CN material with Ni SA anchored at the edge of g‐C_3_N_4_ nanosheet delivers a high single atomic active site density, confirmed by the related electron microscopy and XAS analysis (**Figure** **15**a,b), which also demonstrated excellent activity and product selectivity towards CO and CH_4_ formation under visible light illumination. Benefited from the improved CO_2_ adsorption achieved through the highly unsaturated Ni–N coordination, the constructed Ni_5_‐CN photo‐catalyst displayed enhanced activities of converting CO_2_ into CO (8.6 µmol g^−1^ h^−1^) and CH_4_ (0.5 µmol g^−1^ h^−1^) under visible light illumination (Figure 15c). Noble metals also have excellent activity in photocatalytic carbon dioxide reduction. For instance, Yang et al.^[^ ^139^ ^]^ fabricated Au single atoms on amino‐group‐modified g‐C_3_N_4_ (U‐ACN) via a mild and eco‐friendly urea reduction method (Figure 15d). Time‐of‐flight secondary ion mass spectrometry (ToF‐SIMS) was employed to reveal the composition of U‐ACN, which suggested that Au atoms formed chemical bonds with the neighboring atoms in the g‐C_3_N_4_ matrix. ToF‐SIMS mapping of the fragment ions further verified the presence of Au bonds and amino groups, both homogeneously dispersed across the U‐ACN matrix. The resulting U‐ACN displayed significantly strengthened activity for CO_2_ photoreduction, with much higher yields of CH_4_ and CO than that of pristine g‐C_3_N_4_ (Figure 15e). After that, the reaction mechanism was explored, and the photoreduction of CO_2_ process may proceed in two paths (Figure 15f): i) the CO_2_ molecules are adsorbed on the N atoms coordinated with Au and gradually reduced by the electrons from Au atoms. The Au–N_x_ species decreased the energy barriers of the adsorbed intermediates, thus favoring the 8e^–^ pathway (Equation (10)), thus facilitating CH_4_ production. (ii) the CO_2_ molecules adsorbed on the amino groups in the U‐ACN matrix are directly reduced by the electrons coming from g‐C_3_N_4_. More recently, rare metal SACs, possessing more sustainable and environmentally friendly and unique electronic and chemical properties, were considered as a promising alternative for constructing C_3_N_4_‐based CO_2_ photoreduction catalysts, which have attracted considerable attention. Wang and teammates prepared Sm SACs on C_3_N_4_ nanosheets (xSm‐CN) through an in‐situ pyrolysis strategy for CO_2_ photoreduction. During the synthesis, the mixture of Sm(NO_3_)_3_/melamine = 3.125 wt.% gradually thermally condenses into an asymmetric coordination structure of Sm–N_8_ between the C_3_N_4_ layers, which was confirmed by the Sm L_3_‐edge XANES and EXAFS profiles (Figure 15g). As the CO_2_ photoreduction performance of the Sm single‐atom catalyst (2Sm‐CN) shown in Figure 15h, the CO generation rate reached 44.27 µmol g^−1^ h^−1^ and a high CO selectivity of 96.8% under simulated sunlight without sacrificial agents, showing a 4.7 times CO generation rate compared to pristine C_3_N_4_ nanosheets. To emphasize the role of Sm SA with the 2Sm‐CN materials, DFT calculations were conducted to give a deep insight understanding of the enhanced mechanism. Theory analysis demonstrated that the excellent CO_2_ photocatalytic performance could be attributed to the promoted absorption and activation of CO_2_ on Sm single active sites, which maintained strong interactions with the oxygen atoms in CO_2_, and lowered the energy barrier for the key intermediate COOH*. Moreover, the abundant pyridinic N in the substrate provides the essential proton for the proton‐coupling mechanism of this COOH* intermediate, promoting the formation of the final product CO (Figure 15i).

**Table 3:** Selected highly efficient SAC@g‐C_3_N_4_ catalysts for photocatalytic CO_2_ reduction.

Samples	Reaction medium	Light source	Major product	Rate max.	TON	Other products	Rate max.	Refs.
g‐C_3_N_4_/CoPc‐COOH	DMF:water: triethylamine	20 W LED (λ > 400 nm)	CH_3_OH	538.75 µmol g^−1^ h^−1^	–	–	–	[170]
Coqpy@mpg‐C_3_N_4_	Acetonitrile‐BIH‐PhOH	100 W Xe lamp (λ > 400 nm)	CO	1.15 µmol (6 mg, 24 h)	128	H_2_	0.06 µmol (6 mg, 24 h)	[171]
Co^2+^@C_3_N_4_	Acetonitrile: TEOA	halogen lamp (λ > 350 nm)	CO	1.056 µmol (1 mg, 2 h)	–	–	–	[120]
Co‐POM	Acetonitrile‐triethanolamine	300 W Xe lamp (λ = 400 ‐ 800 nm)	CO	17 µmol g^−1^ h^−1^	–	CH_4_	0.7 µmol g^−1^ h^−1^	[356]
Co‐(bpy)_3_Cl_2_/g‐CN	Acetonitrile‐triethanolamine	300 W Xe lamp (λ > 420 nm)	CO	3.7 µmol (50 mg, 2 h)	4.3	H_2_	0.6 µmol (50 mg, 2 h)	[354]
Fe(qpy)/mpg‐C_3_N_4_	Acetonitrile‐triethanolamine	400 W Hg lamp (λ > 400 nm)	CO	12.4 µmol (8 mg, 17 h)	155	HCOOH	0.7 µmol (8 mg, 17 h)	[357]
Au‐ACN	ethanol	300 W Xe lamp (λ > 420 nm)	CO	21.7 µmol g^−1^ (2.5 h)	–	CH_4_	2.4 µmol g^−1^ (2.5 h)	[139]
Ru/mpg‐C_3_N_4_	Acetonitrile‐triethanolamine	450 W Xe lamp (λ > 400 nm)	HCOOH	19.345 µmol (8 mg, 5 h)	–	CO	2.997 µmol (8 mg, 5 h)	[353]
Ru‐complex/mpg‐C_3_N_4_	Acetonitrile‐triethanolamine	400 W Hg lamp (λ > 400 nm)	HCOOH	1.854 µmol (8 mg, 5 h)	–	–	–	[352]
RuP/C_3_N_4_	Dimethylacetamide‐ triethylamine	400 W Hg lamp (λ > 400 nm)	HCOOH	68 µmol (8 mg, 20 h)	–	CO/H_2_	18 µmol/0.9 µmol (8 mg, 20 h)	[347]
RuP/C_3_N_4_	Dimethylacetamide‐triethanolamine	400 W Hg lamp (λ > 400 nm)	HCOOH	8.8 µmol (8 mg, 1 h)	141	CO	0.7 µmol (8 mg, 1 h)	[349]
RuP/C_3_N_4_	Acetonitrile‐triethanolamine	400 W Hg lamp (λ > 400 nm)	HCOOH	–	≈170	–	–	[351]
RuRu’/Ag/mpg‐C_3_N_4_	EDTA·2Na+ Na_2_CO_3_	400 W Hg lamp (λ > 400 nm)	HCOOH	4.1 µmol (4 mg, 15 h)	–	H_2_	0.1 µmol (4 mg, 15 h)	[350]
RuRu’/Ag/C_3_N_4_	Dimethylacetamide‐triethanolamine	400 W Hg lamp (λ > 400 nm)	HCOOH	42.3 µmol (4 mg, 5 h)	3110	H_2_	0.3 µmol (4 mg, 5 h)	[348]

**Figure 16:**
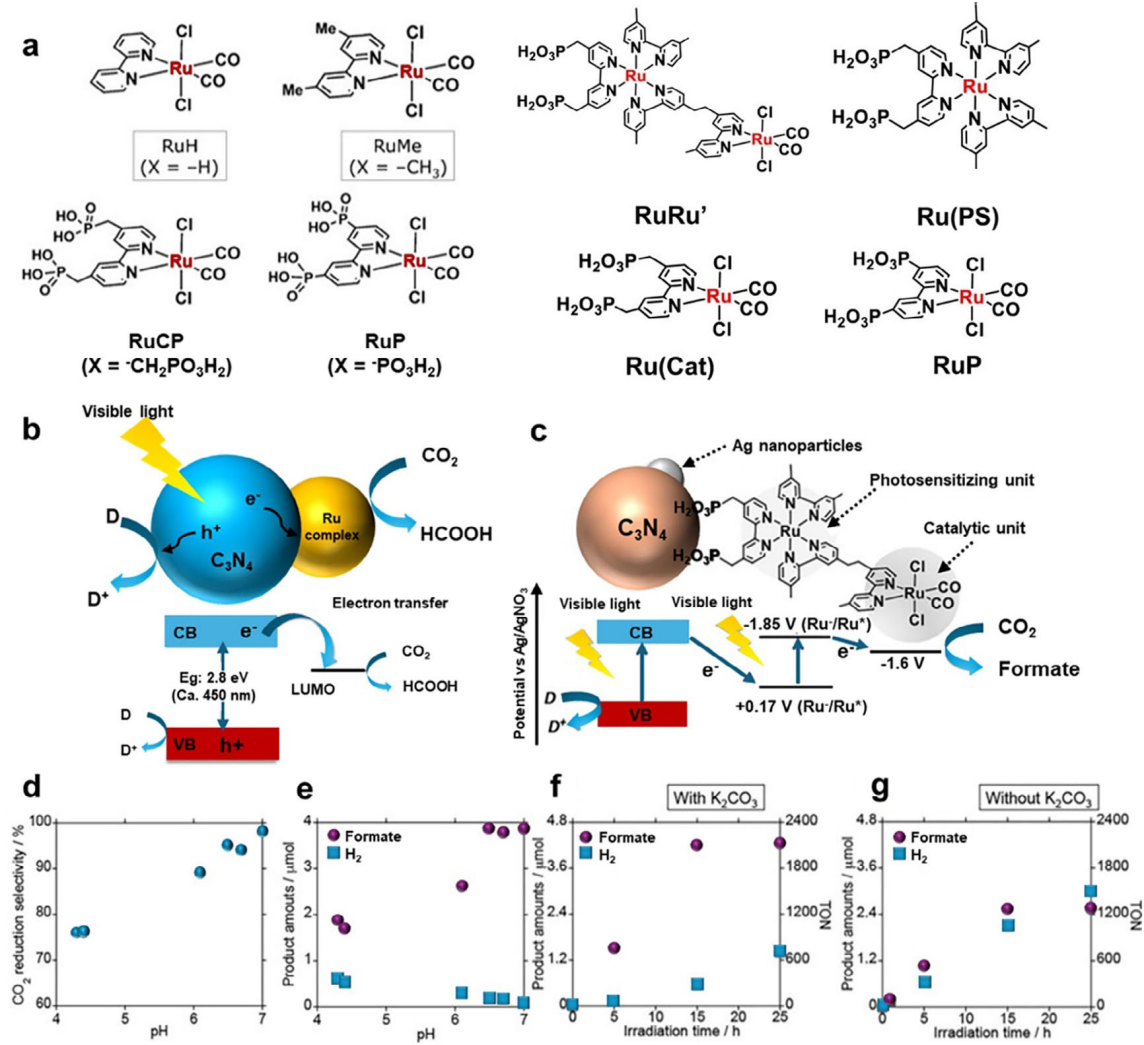
a) The structures of the studied Ru complexes. Reproduced with permission.^[^ ^348^, ^349^ ^]^ Copyright 2016, American Chemical Society, Copyright 2015, John Wiley and Sons. b) CO_2_ reduction using a Ru complex/C_3_N_4_ hybrid photocatalyst. Adapted with permission.^[^ ^349^ ^]^ Copyright 2015, John Wiley and Sons. c) Z‐Scheme electron transfer during CO_2_ reduction using a hybrid photocatalyst consisting of Ag‐loaded C_3_N_4_ and Ru^II^ binuclear complex (RuRu’). d) Selectivity for CO_2_ reduction versus pH value of the reaction solution during the photocatalytic reactions and e) amounts of formate and H_2_ generated versus pH value of the reaction solution. f) Time courses of formate and H_2_ formation during photoirradiation (λ > 400 nm) using RuRu’ (0.5 µmol g^−1^)/Ag(0.5wt.%)/NS‐C_3_N_4_ (4 mg) in aqueous solution (4 mL) containing EDTA 2Na (10 mM) with and g) without K_2_CO_3_ (0.1 M). Reproduced with permission.^[^ ^350^ ^]^ Copyright 2017, John Wiley and Sons.

Metal complexes are another way to involve atomically dispersed reactive sites for photocatalytic CO_2_ reduction. In this field, Maeda and collaborators have developed many pioneering and systematic studies.^[^ ^347^, ^348^, ^349^, ^350^, ^351^, ^352^, ^353^ ^]^ A series of Ru‐based metal complexes were studied on g‐C_3_N_4_ as a photosensitizer for photocatalytic CO_2_ reduction (**Figure** **16**a). For instance, a molecular Ru^II^ complex, RuP: *trans*(Cl)‐[Ru(bpyX_2_)(CO)_2_Cl_2_] (bpyX_2_ = 2,2′‐bipyridine with PO_3_H_2_ in the four‐positions, was immobilized on g‐C_3_N_4_. The RuP has been confirmed to play two important roles: i) as an electron acceptor for receiving photoexcited electrons from the CB of g‐C_3_N_4_ and ii) as a host for the active centers for CO_2_ reduction (Figure 16b).^[^ ^349^ ^]^ The reduction potential of RuP is found to be –1.4 V versus Ag/AgNO_3_, which is more positive than the CB minimum for g‐C_3_N_4_ of –1.65 V versus Ag/AgNO_3_, leading to fast electron transfer kinetics. As a result, RuP/g‐C_3_N_4_ showed a suitable reaction environment and efficient photocatalytic conversion of CO_2_ into HCOOH, demonstrating a high TON of 141 and AQY of 5.7% at λ = 400 nm under visible‐light irradiation. This also reveals that the structure and properties of the Ru^II^ complex are key factors in photocatalytic CO_2_ reduction. Rational design and construction of a heterostructure on a molecular scale can promote the injection of photoexcited electrons from g‐C_3_N_4_ into the Ru^II^ unit and strengthen the electronic interactions between the two units, thereby adjusting the catalytic functionality. In another work, they further modified g‐C_3_N_4_ with a Ru^II^ binuclear complex and Ag nanoparticles to construct a nature‐inspired artificial Z‐scheme CO_2_ photoreduction system (Figure 16c).^[^ ^350^ ^]^ The Ag nanoparticles were used to facilitate electron transfer from g‐C_3_N_4_ to the excited state of the Ru^II^ binuclear complex, and the two Ru^II^ metals were expected to drive an efficient two‐step photoexcitation: one acts as the photosensitizer to construct the Z‐Scheme with g‐C_3_N_4,_ and the other is the active site for absorbing and catalyzing CO_2_ molecules. All these components worked in concert and contributed to durable, selective CO_2_ reduction with high selectivity (≈98%) to HCOOH and an outstanding TON above 2000 in aqueous media. Furthermore, the influence of pH and the additive salts on the photocatalytic performance was investigated. The CO_2_ selectivity as a function of pH showed a linearly increasing relationship with the same trend as the HCOOH generation rate and pH (Figure 16d,e). This is primarily due to the negative shift of the CB and VB positions of g‐C_3_N_4_ with pH, which increases the driving force for the transfer of photoexcited electrons from the CB of g‐C_3_N_4_ to the Ru^II^ binuclear complex. In addition, a significant enhancement in activity and selectivity was achieved with the additives of K_2_CO_3_ (Figure 16f,g). Although the details of the impact of K^+^ cations during the photocatalytic reactions are still unclear, it seems that some interactions exist between the K^+^ cations and g‐C_3_N_4_ that positively affect the CO_2_ reduction process.

**Figure 17:**
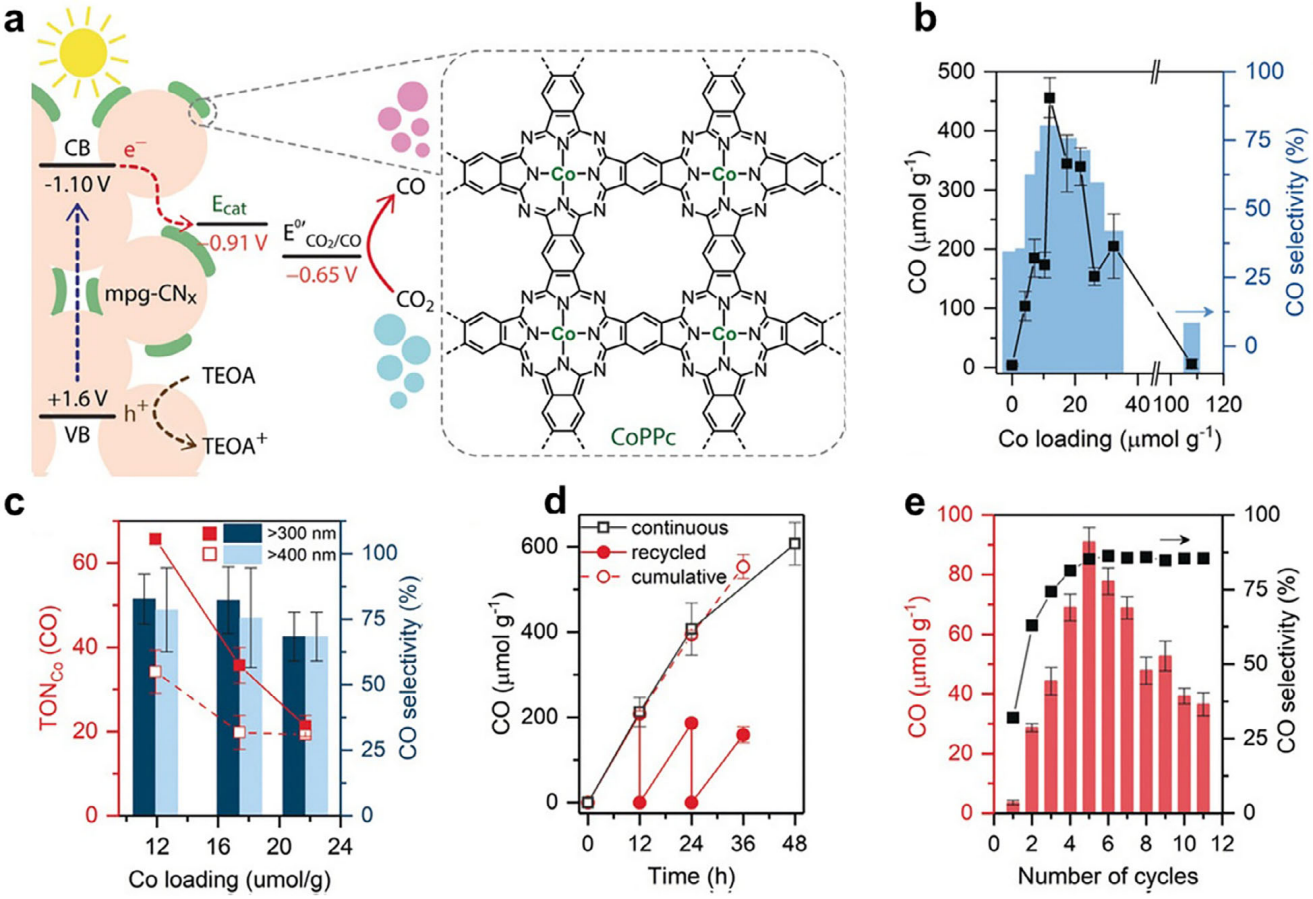
a) Schematic of photocatalytic CO_2_ reduction on mpg‐CN_x_|CoPPc under AM 1.5G irradiation. b) Calculated CO production amount (black trace) and selectivity (blue bars) on mpg‐CN_x_|CoPPc catalysts with different Co loading recording in CO_2_‐saturated MeCN/TEOA solution under AM 1.5G irradiation (100 mW cm^−2^, λ > 400 nm) for 24 h. c) Calculated TONs based on metal Co (TON_Co_) for CO production after reacting 48 h, the dashed red curves were recorded under visible irradiation (λ > 400 nm), the solid red curves were recorded under UV/Vis (λ > 300 nm) irradiation, and the bar plots represent the corresponding selectivity for CO production. d,e) The recycling performances of the mpg‐CN_x_|CoPPc catalyst under visible light irradiation. d) The solid red curve shows the CO production amount during three cycles, the dashed red curve shows the calculated cumulative CO amount, and the black curve shows the CO accumulated amount under continuous irradiation, with the catalyst loading of 2 mg of the mpg‐CN_x_|CoPPc_11.9_. e) Histogram with error bars of the CO production amount during ten 4 h recycling runs with the catalyst loading of 8 mg of the mpg‐CN_x_|CoPPc_27_, the black curve displays the corresponding selectivities. Reproduced with permission.^[^ ^355^ ^]^ Copyright 2019, John Wiley and Sons.

In addition to the Ru metal complex, several other metal complex was also loaded on g‐C_3_N_4_ for photocatalytic CO_2_ reduction, such as Co,^[^ ^170^, ^171^, ^354^, ^355^, ^356^ ^]^ and Fe^[^ ^357^ ^]^ complex. Roy et al.^[^ ^355^ ^]^ reported a photocatalytic system consisting of polymeric cobalt phthalocyanine (CoPPc) and mesoporous g‐C_3_N_4_ (mpg‐CN_x_) for visible‐light‐driven CO_2_ reduction (**Figure** **17**a). This hybrid catalyst exhibited remarkably selective conversion of CO_2_ into CO in the presence of organic solvents under AM 1.5G irradiation. The photocatalytic activity was found to be greatly dependent on the loading of CoPPc, and the optimal cobalt loading was assigned to 11.9 µmol Co g^−1^ (Figure 17b). Such a suitable loading well‐balanced the accessible active sites and the light‐absorbing areas, where the photogenerated electrons were transferred from mpg‐CN_x_ to CoPPc and effectively trapped by Co^II^ sites to form Co^I^, then the Co^I^ sites convert the adsorbed CO_2_ to CO. Consequently, the mpg‐CN_x_|CoPPc_11.9_ exhibited a TONs value of 51 under visible‐light irradiation and 84 under the full solar spectrum, and the recycling test and long‐term experiment demonstrated excellent photoreducing properties (Figure 17c–e). Ye and coworkers^[^ ^356^ ^]^ reported covalently linked Co‐porphyrin and g‐C_3_N_4_ for the photoreduction of CO_2_ to CO, which showed a similar electron transfer and trapping mechanism for enhancing the activity. Cometto et al.^[^ ^357^] fabricated an iron‐complex catalyst on mesoporous g‐C_3_N_4_. Despite its simplicity, this system has been proven to be a promising catalytic platform for photostimulated CO_2_‐to‐CO reduction.

It is concluded that single‐site catalysts are very useful for the adsorption and further activation of CO_2_ due to the accessible empty orbits on their coordinative environment and can effectively alter the electronic structures of g‐C_3_N_4_ for enhanced light‐harvesting efficiency. Additionally, metal complexes are another simple and efficient approach for further improvement and implementation in devices while using only earth‐abundant and low‐cost components.

#### 4.2.3 Other Photocatalytic Reactions

With the pressing issues of environmental pollution and emphasis on sustainable development, there is an increasing urgency to explore different kinds of semiconductor‐based photocatalysis technologies driven by solar energy,^[^ ^358^ ^]^ such as the photocatalytic degradation of organic compounds, including rhodamine B (RhB),^[^ ^172^, ^173^, ^179^, ^190^ ^]^ methyl orange,^[^ ^190^ ^]^ methylene blue,^[^ ^213^ ^]^ 4‐chlorophenol,^[^ ^173^, ^359^ ^]^ *p*‐nitrophenol,^[^ ^183^ ^]^ naproxen,^[^ ^210^ ^]^ sulfamethazine,^[^ ^211^ ^]^ bisphenol A,^[^ ^212^ ^]^ sulfaquinoxaline sodium,^[^ ^172^ ^]^ carbamazepine,^[^ ^172^ ^]^ alcohols,^[^ ^360^ ^]^ 1,4‐dihydropyridines.^[^ ^360^ ^]^

**Figure 18:**
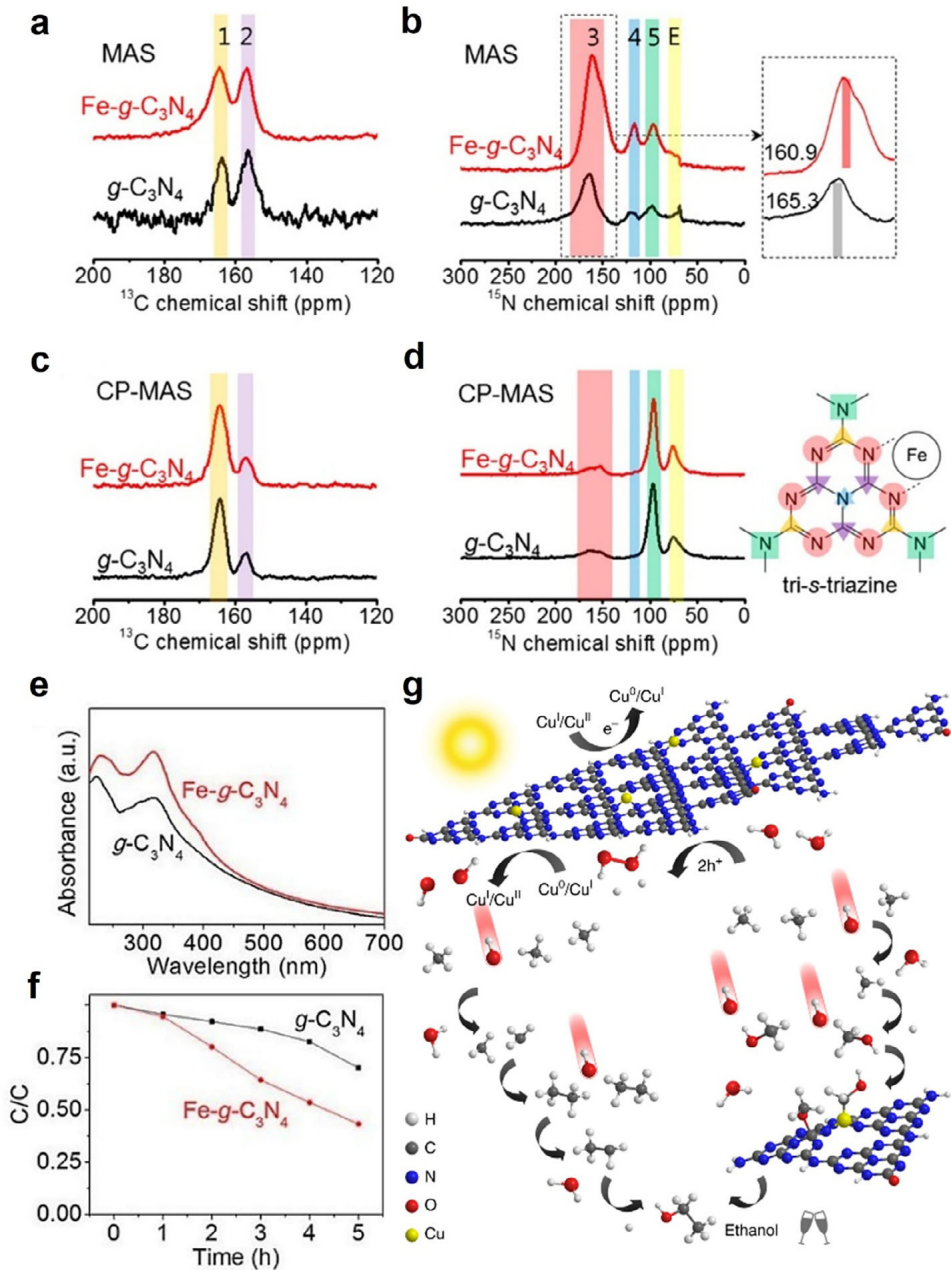
a) ^13^C MAS ssNMR and b) ^15^N MAS ssNMR spectra of Fe‐g‐C_3_N_4_ and g‐C_3_N_4_. c) ^13^C and d) ^15^N MAS ssNMR spectra with cross‐polarization (CP‐MAS) setup for Fe‐g‐C_3_N_4_ and g‐C_3_N_4_. e) UV–vis absorption spectra of Fe‐g‐C_3_N_4_ and g‐C_3_N_4_. f) Photocatalytic activity of Fe‐g‐C_3_N_4_ and g‐C_3_N_4_ for PNP degradation in aqueous solution under irradiation. Reproduced with permission.^[^ ^183^ ^]^ Copyright 2016, American Chemical Society. g) The hypothetical mechanism for photocatalytic anaerobic methane conversion over Cu‐0.5/PCN. Reproduced with permission.^[^ ^187^ ^]^ Copyright 2019, Springer Nature.

For instance, Oh et al.^[^ ^183^ ^]^ presented a divalent Fe‐coordinated g‐C_3_N_4_ system and carefully investigated its bond coordination, electronic structure variation, and influence on photocatalytic activity. As shown in **Figure** **18**a–d, the structural information was first studied using magic angle spinning solid‐state nuclear magnetic resonance spectroscopy (MAS ssNMR) and coupling with cross‐polarization (CP‐MAS) setup to monitor hydrogen‐coupled nuclei for distinguishing the different types of carbon and nitrogen. The overall structure of the intrinsic g‐C_3_N_4_ matrix did not interfere with the functionalization of the Fe species, and the slight chemical downshift in the ^15^N NMR spectra revealed a reduced overall electronegativity of g‐C_3_N_4_ through electron sharing. They demonstrated that the isolated Fe atoms were anchored and stabilized at the center of the structural cavities created by *tri‐s*‐triazine units coordinated by the edge side of nitrogen, which is in accordance with the above‐mentioned analysis from other characterizations. The obtained Fe‐g‐C_3_N_4_ showed enhanced adsorption in the UV–vis range and a redshifted absorption wavelength due to the generation of more electron‐hole pairs under light irradiation (Figure 18e). Photodegradation of *p*‐nitrophenol indicated that the pristine and Fe‐g‐C_3_N_4_ decomposed by 27% and 55% within 5 h, respectively, suggesting that divalent Fe functioned as the carrier trapping sites for significantly improving the exciton separation efficiency and the interfacial electron transfer process (Figure 18f). In addition, Ni(I)‐N active sites on g‐C_3_N_4_ achieved highly efficient photocatalytic H_2_O_2_ production, which was attributed to the activation of molecular O_2_.^[^ ^191^ ^]^ Engineering cobalt into the framework of g‐C_3_N_4_ activates the peroxymonosulfate (PMS) into sulfate radical (SO_4_^•−^) was considered as the major active radicals for the monochlorophenols (MCPs) isomers degradation.^[^ ^361^ ^]^ Moreover, Copper‐modified g‐C_3_N_4_ exhibited a promising ability towards the direct valorization of methane to its alcohol derivative under irradiation (Figure 18g),^[^ ^187^ ^]^ which was capable of managing the generation and in situ decomposition of H_2_O_2_ to produce ·OH and provide abundant Cu active centers for the adsorption and activation of methane. This feature effectively prevents excess OH for deep mineralization, accelerating the photocatalytic conversion of anaerobic methane to ethanol. Importantly, the synergy of isolated Cu atoms and adjacent C atoms was found to play a critical role in the methane–methanol–ethanol pathway. Therefore, increased attention should be directed toward SACs@g‐C_3_N_4_ systems to fundamentally understand their composition‐structure‐topology‐property relationships through combined experimental and theoretical approaches, which will ultimately guide the rational design and exploitation of these advanced materials for novel photocatalytic applications.

### 4.3 Organic Chemical Transformations

In the modern chemical industry, heterogeneous catalysis is currently undergoing a revolution to achieve the goals of adapting diverse renewable feedstocks and reducing their impact on the environment.^[^ ^362^, ^363^, ^364^, ^365^ ^]^ There are two main concerns regarding the development of novel functional catalysts besides activity: i) selectivity, especially at high conversions, and ii) stability, which should maintain catalytically active sites even under harsh conditions.^[^ ^366^ ^]^ Precious metals such as Pt, Au, and Pd are currently the major commercial catalysts. However, their high cost and scarcity remain the primary obstacles to further progress. Great efforts have been made to tackle this issue specifically. The now popularized concept of single‐site heterogeneous catalysts brings up a new avenue to maximum atom‐utilization efficiency. It is explicitly stated that the performance of SACs is key to the host materials, as it determines the coordination environment and the related electronic properties of the metal center.^[^ ^25^, ^367^ ^]^ In this section, we summarize recent advances in the theoretical and experimental investigations of SACs@g‐C_3_N_4_ for a variety of reactions, including hydrogenation reactions, Suzuki coupling reactions, and alkene epoxidation reactions. Moreover, the relationship between the structure and catalytic selectivity, stability, and activity of SACs@g‐C_3_N_4_ along with the catalytic mechanism is also discussed.

#### 4.3.1 Hydrogenation Reactions

Selective hydrogenation is one of the most important processes for a wide range of industrial processes. Pd‐based systems are commonly employed due to their high activities, but they tend to induce over‐hydrogenation pathways and undesired isomerization. Thus, several promoters, such as organic ligands and lead, have been fabricated to optimize the adsorption energies of the reactants and products, as well as to decrease the size of the ensemble of surface atoms where the reactions occur. To this end, tailoring the particle size is another effective approach for improving the performance of conventional catalysts. A single‐site heterogeneous catalyst represents an ultimate case of Pd‐based catalysts, where isolated Pd atoms stabilized on a substrate catalyze hydrogenation reactions.

**Figure 19:**
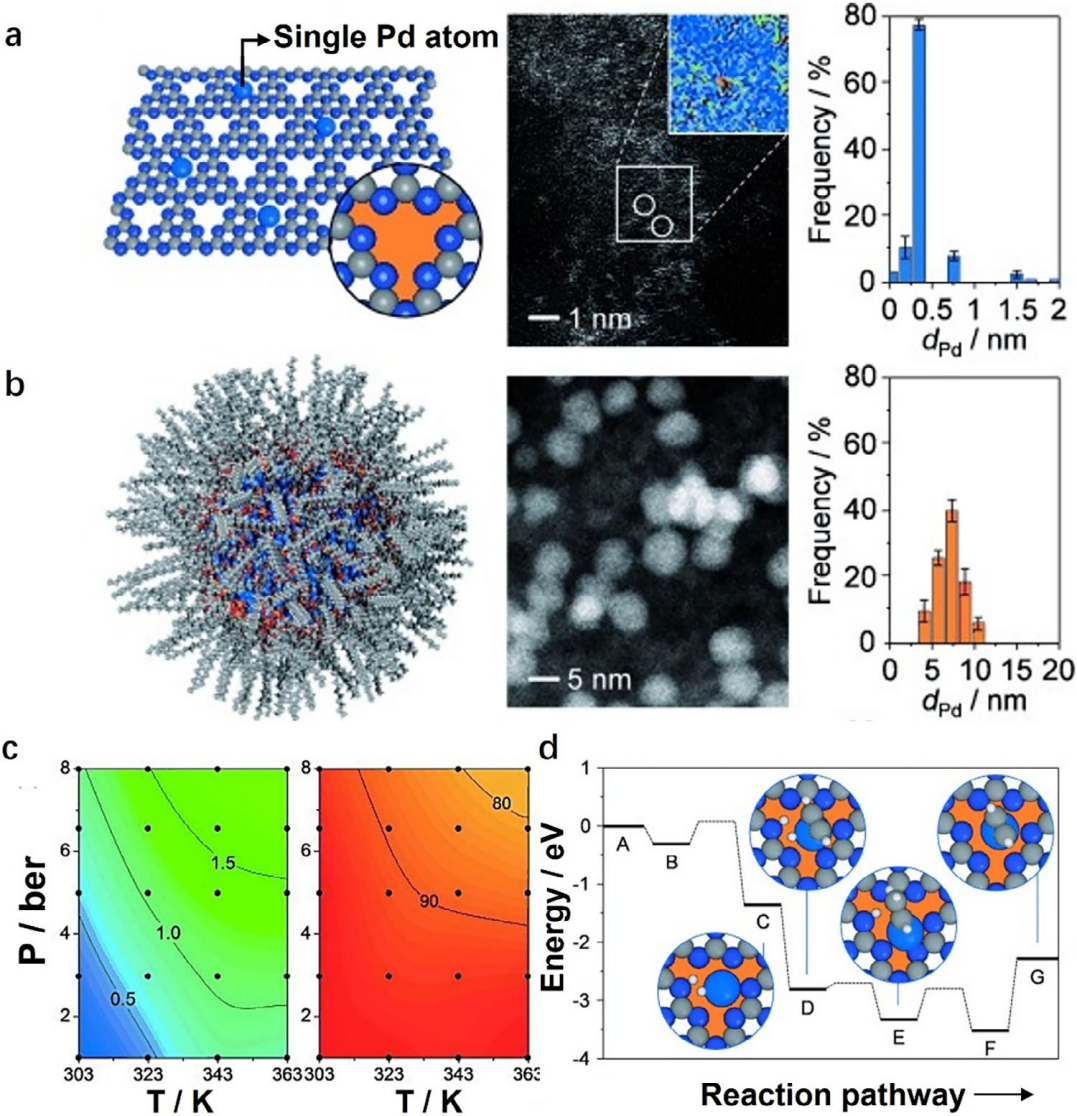
Schematic illustration of the catalysts (left), AC‐STEM images (middle), and the calculated histogram of the particle size distribution (right) of a) [Pd]mpg‐C_3_N_4_, b) Pd‐HHDMA/TiS. c) Rates of the hydrogenation of 1‐hexyne (in 10^3^ mol_product_ mol_Pd_^−1^ h^−1^. left) and the selectivity to 1‐hexene (in %, right) at different temperatures and pressures on [Pd]mpg‐C_3_N_4_. d) Energy profile for acetylene hydrogenation on the structure of an isolated Pd single atom on g‐C_3_N_4_. The dark gray, purple, red, white, and blue balls represent the C, N, O, H, and Pd atoms, respectively. Reproduced with permission.^[^ ^68^ ^]^ Copyright 2015, John Wiley and Sons.

Flytzani Stephanopoulos and co‐workers^[^ ^368^, ^369^ ^]^ for the first time reported a single crystal comprising isolated Pd atoms with Cu (111), which can activate molecular H_2_, rendering it more efficient for selective hydrogenation of acetylene and styrene under ultra‐high vacuum conditions. Javier Perez‐Ramirez and co‐workers^[^ ^68^ ^]^ anchored the single Pd atoms into the cavities of mesoporous polymeric g‐C_3_N_4_ and employed this novel composite for tri‐phase hydrogenations of nitroarenes and alkynes. The AC‐STEM images in **Figure** **19**a demonstrate the atomic dispersion of isolated Pd atoms on g‐C_3_N_4_ ([Pd]mpg‐C_3_N_4_), without the formation of aggregates or particles. Further detailed particle size distribution analysis showed an average metal size of 0.3–0.4 nm, matching well with the van der Waals diameter of a single Pd atom. For comparison, the benchmark catalysts of ligan‐modified Pd‐HHDMA/TiS in industry were also investigated, which revealed a spherical morphology with an approximate size of 8 nm under the same metal loading as [Pd]mpg‐C_3_N_4_ (Figure 19b); that is, [Pd]mpg‐C_3_N_4_ can expose more (almost all) surface atoms to participate in the reactions. As a result, when [Pd]mpg‐C_3_N_4_ was used as a catalyst for the hydrogenation of 1‐hexyne, a typical alkyne compound, it showed a remarkably enhanced rate at 303 K and 1 bar, 3 orders of magnitude higher than other catalytic systems, such as Au, Ag, and CeO_2_‐based catalyst (Figure 19c). Furthermore, the selectivity of 1‐hexyne to 1‐hexene was nearly 100% at 363 K and 2 bar, demonstrating that the novel [Pd]mpg‐C_3_N_4_ catalysts hold great promise for use in the manufacture of fine chemicals and pharmaceuticals. This exceptional hydrogenation performance of [Pd]mpg‐C_3_N_4_ was then rationalized by DFT calculations at a molecular level, as illustrated in Figure 19d, where acetylene was used as a surrogate alkyne. The isolated Pd sites functioned as active centers, leading to facile hydrogen activation and alkyne adsorption, resulting in high activity and selectivity.

In a subsequent work, Büchele et al.^[^ ^370^ ^]^ studied different types of nitrogen‐doped carbons with tunable nitrogen contents as hosts for Pd atoms. It was confirmed that the nitrogen species and contents are crucial for achieving the atomic dispersion of Pd and further influence its average oxidation states. The synthesis‐property‐performance relationships were then established to guide the development of more efficient SACs. Although the comparable catalytic performance is achievable over isolated Pd on nitrogen‐doped carbons, the g‐C_3_N_4_ is still an optimal host for stabilizing isolated Pd atoms, and various studies disclosed the as‐obtained Pd@g‐C_3_N_4_ possessed high stability and selectivity on hydrogenation of 2‐methyl‐3‐butyn‐2‐ol to 2‐methyl‐3‐buten‐2‐ol,^[^ ^113^, ^115^ ^]^ 1‐hexyne to 1‐hexene,^[^ ^108^ ^]^ acetylene to ethylene,^[^ ^220^ ^]^ 4‐vinylphenol^[^ ^371^ ^]^ and carbon dioxide to formic acid,^[^ ^110^ ^]^ and semi‐hydrogenation of alkynes,^[^ ^372^, ^373^, ^374^, ^375^ ^]^ alkynols,^[^ ^376^ ^]^ respectively. Furthermore, Chen et al.^[^ ^116^ ^]^ investigated the impact of heteroatom doping g‐C_3_N_4_ with boron, fluorine, sulfur, and phosphorus on the interaction and associated catalytic performance of isolated Pd atoms. The stability of the isolated Pd atoms was determined by the dopant content, and phosphorus doping resulted in an appreciable increase in the electron density of g‐C_3_N_4_, thereby reducing the oxidation state of Pd. Evaluation of the semi‐hydrogenation of 2‐methyl‐3‐butyn‐2‐ol showed a significantly higher reaction rate and a desired selectivity of nearly 100% for Pd@P‐g‐C_3_N_4_.

**Figure 20:**
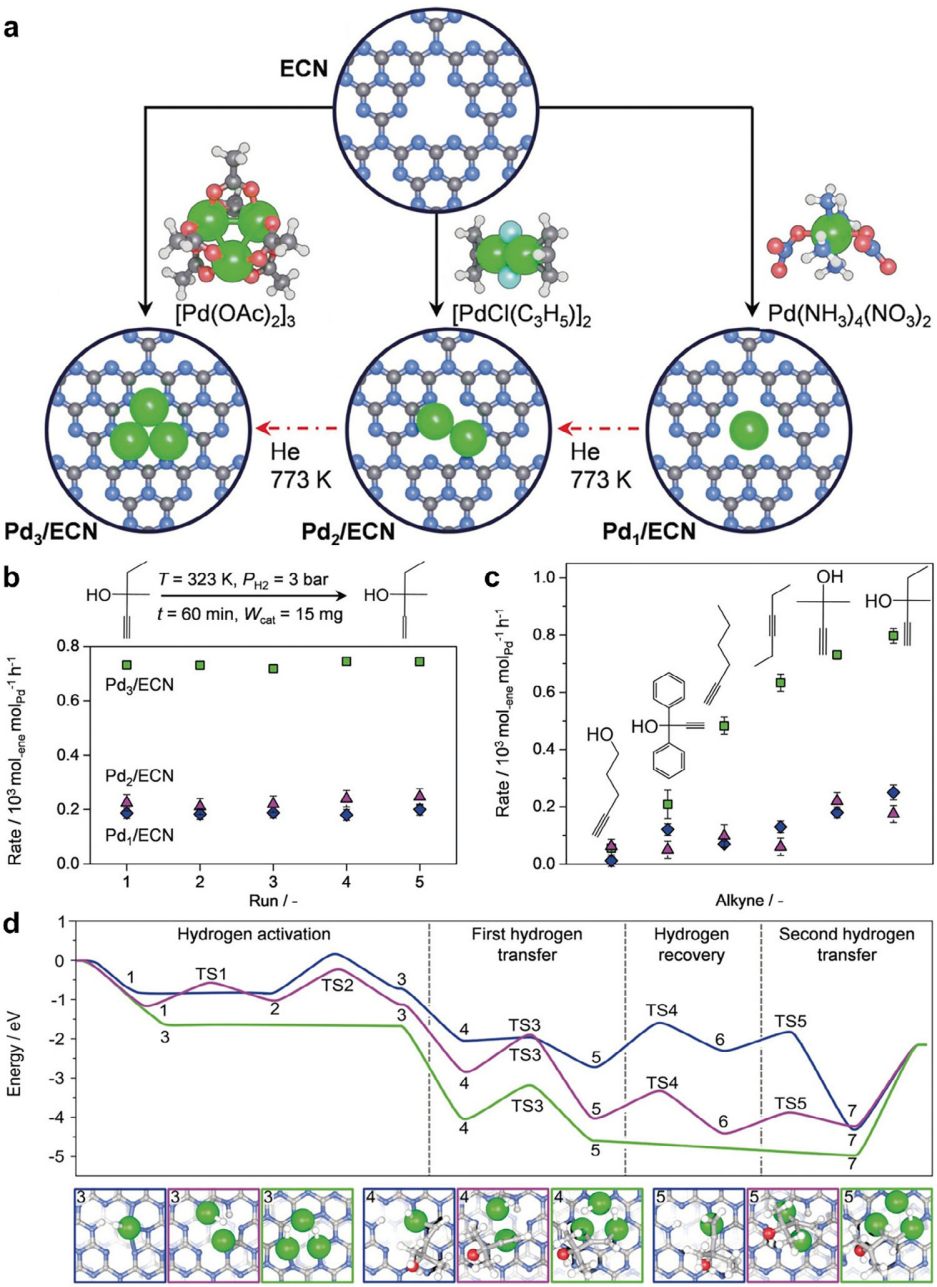
a) Approaches to prepare low‐nuclearity Pd catalysts based on carbon nitride. b) Variation in the rates of 2‐methyl‐3‐buten‐2‐ol formation in the hydrogenation of 2‐methyl‐3‐butyn‐2‐ol (depicted) over five sequential runs and c) the calculated reaction paths over the surface configurations (with all atoms at the surface) of the Pd ensembles in Pd_x_/ECN. d) Rates of alkene formation in the hydrogenation of distinct functionalized alkynes (illustrated). Reproduced with permission.^[^ ^117^ ^]^ Copyright 2019, John Wiley and Sons.

To demonstrate the versatile coordination chemistry of g‐C_3_N_4_, Vorobyeva et al.^[^ ^117^ ^]^ exploited the multidentate coordination of the heptazine heterorings of g‐C_3_N_4_ to accommodate isolated Pd atoms, dimers, and trimers (**Figure** **20**a). Controlled incorporation of low‐nuclearity clusters was achieved by depositing dimeric and trimeric Pd complexes on g‐C_3_N_4_ (Pd_x_/ECN). The AC‐STEM images verified the high metal dispersion in the Pd_x_/ECN systems, and the EXAFS analysis revealed that the average Pd–N coordination number was reduced on Pd dimers and trimers with respect to Pd_1_/ECN, owing to the less pronounced interaction with the g‐C_3_N_4_ matrix. All of these catalysts exhibited full chemoselectivity (100%) towards the hydrogenation of 2‐methyl‐3‐butyn‐2‐ol. Importantly, the Pd_3_/ECN possessed an extremely high reaction rate, three orders of magnitude higher than that of Pd_1_/ECN and Pd_2_/ECN (Figure 20b‐c). This was linked to the homolytic H_2_ activation path on Pd_3_/ECN, which required at least an ensemble of three atoms and provided a bridge site for the absorption of the alkyne. However, Pd_1_/ECN and Pd_2_/ECN underwent a heterolytic route, and an H atom was transferred to the g‐C_3_N_4_ matrix (Figure 20d). The coordination sphere of Pd changed following the mechanism to accommodate triple‐bond adsorption and the subsequent H transfer. A higher barrier was generated for this step, as well as for the second H recovery to the metal center. The slightly increased performance of Pd_2_/ECN is mainly derived from the possibility of both Pd atoms being located in surface positions, leading to the existence of a bridge site and a more exothermic hydrogen activation than Pd_1_/ECN. Moreover, Chen and co‐workers reported a precursor‐preselected strategy to construct Pd diatomic catalysts (Pd_2_‐mpg‐C_3_N_4_) for harnessing ensemble effects between the dual‐Pd species.^[^ ^371^ ^]^ Isotopic‐labeling experiments and operando nuclear magnetic resonance demonstrated that the Pd_2_‐mpg‐C_3_N_4_ with diatomic Pd as the active site follows the previously confirmed mechanism, using water as the proton source, achieving an excellent yield of 92% for photocatalytic hydrogenation of 4‐vinylphenol to 4‐ethylphenol under atmospheric conditions and visible‐light irradiation. Additionally, the Pd@C_3_N_4_ materials with complex valence of Pd species could also deliver excellent hydrogenation performance for unsaturated organic molecules,^[^ ^377^ ^]^ exhibiting a superior commercial application potential.

Apart from metal Pd, Jia et al. synthesized a mesoporous g‐C_3_N_4_‐supported isolated single‐atom Ni catalyst (Ni−N/CN) by a mild solvothermal deposition approach, which showed outstanding activity and selectivity for semihydrogenation of various alkynes with water as the proton source. DFT calculation corroborated that isolated Ni atoms can diffuse between the site coordinating with four nitrogen atoms and the site coordinating with three nitrogen atoms. During the semihydrogenation reaction, photoinduced electron transfer to Ni^2+^ occurs simultaneously with water dissociation, yielding Ni^+^, where a proton is transferred from water to the uncoordinated pyridine N, thereby transferring the surface (N)H to the alkyne, achieving a highly selective semihydrogenation of the alkyne.^[^ ^373^ ^]^ Arcudi and coworkers reported a reduction treatment strategy for stabilizing atomically dispersed Co into the framework of g‐C_3_N_4_ nanosheets obtained by thermal exfoliation. Benefited from the heterogeneous nature of CN and the unique coordination environment of Co‐CN, the constructed photocatalysts could be easily isolated, which could achieve excellent selectivity, stability, and long life‐running for up to 960 hours without loss of activity, and maintaining ≥99.9% selectivity to ethylene (relative to ethane and H_2_).^[^ ^374^ ^]^ He et al.^[^ ^378^ ^]^ investigated the potential of Pt@g‐C_3_N_4_ for the hydrogenation of nitrobenzene into aniline using DFT calculations. The overall activation energy barriers on Pt@g‐C_3_N_4_ were found to be lower than those on the Pt (111) surface, and a single H‐induced dissociation of the N–O bands energetically favored the hydrogenation reaction. Moreover, the g‐C_3_N_4_‐supported SACs with Ru,^[^ ^141^ ^]^ Ag,^[^ ^379^ ^]^ and Pt^[^ ^375^ ^]^ as the active sites also show outstanding activity and selectivity for hydrogenation in the field of photocatalysis.

Therefore, SACs@g‐C_3_N_4_ represent the next generation of highly efficient catalysts for hydrogenation reactions, demonstrating substantial potential for industrial implementation. To fully realize this potential, focused research should prioritize precise control of metal‐carrier interactions through coordination engineering, strategic exposure of active metal centers at surface layers, and provide atomistic‐level mechanistic insights into the catalytic hydrogenation pathways.

#### 4.3.2 Suzuki Coupling Reactions

**Figure 21:**
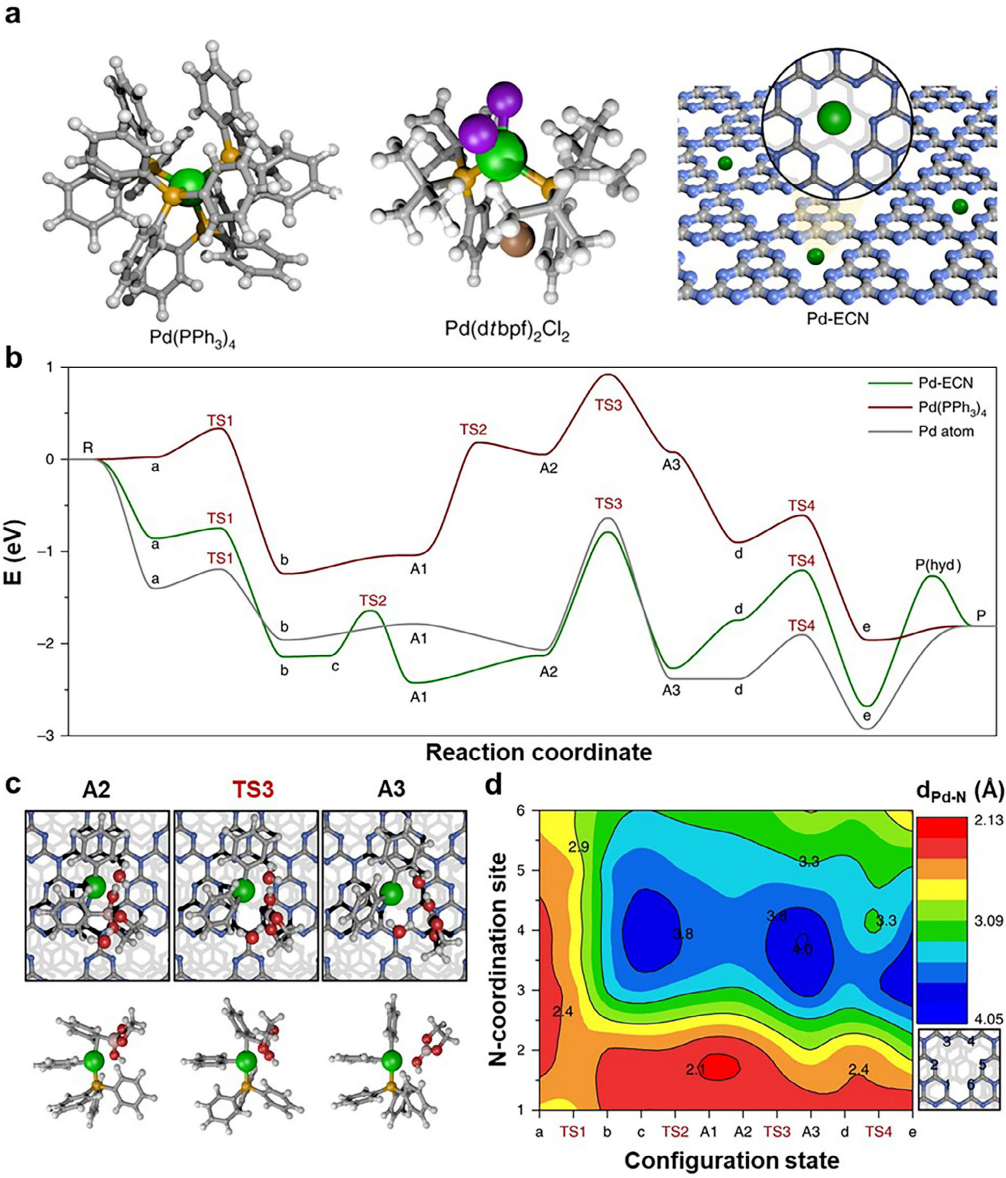
a) Structures of the homogeneous Pd catalysts and the heterogeneous Pd‐ECN catalyst. b) Energy profiles of the Suzuki coupling of bromobenzene with phenylboronic acid pinacol ester over an isolated Pd single atom, Pd(PPh_3_)_4_, and Pd‐ECN catalysts. c) The transmetallation step (A2 → A3) for the indicated configuration of Pd‐ECN (top) and Pd(PPh_3_)_4_ (bottom). The dark gray, blue, red, white, and green balls represent the C, N, O, H, and Pd atoms, respectively. d) Contour plot of the interatomic Pd–N distances for each N‐coordination site (schematically identified in the bottom right) at each of the intermediate and transition states indicated in (b). Reproduced with permission.^[^ ^114^ ^]^ Copyright 2018, Springer Nature.

Suzuki–Miyaura coupling has emerged as an indispensable tool for the construction of carbon‐carbon bonds and is widely used to synthesize natural products, bioactive compounds, and some advanced materials.[^380^, ^381^, ^382^ ^]^ In traditional cases, homogeneous palladium complexes with organic ligands are commonly employed as catalysts, such as Pd(PPh_3_)_4_ (tetrakis(triphenylphosphine)palladium) and Pd(dtbpf)_2_Cl_2_ ([1,1′‐bis(di‐tert‐butylphosphino)ferrocene]dichloropalladium(ii)) (**Figure** **21**a), thereby recognizing the challenges of product purification and catalyst reusability that remain to be overcome for their industrial applications. An alternative strategy is to tether these homogeneous catalysts to suitable substrates. however, it suffers from the metal leaching or clustering phenomenon during the catalytic process, leading to progressively worsening performance.^[^ ^383^ ^]^

To address these issues, Chen et al.^[^ ^114^ ^]^ stabilized isolated Pd single atoms on g‐C_3_N_4_ (Pd‐ECN) as heterogeneous SACs for Suzuki coupling reactions, which aim to not only inherit the merits of uniform active sites and high chemoselectivity but also capture the advantages of easy separation and distinguished recyclability. As illustrated in Figure 21b–d, the reaction path of Pd‐ECN in the continuous Suzuki coupling of bromobenzene with phenylboronic acid pinacol ester was simulated by DFT and compared with the benchmark homogeneous and heterogeneous systems. The initial coordination number of Pd in the lattice was normalized to six, at which the value showed a low barrier for the adsorption and activation of bromobenzene. Then, the isolated Pd on g‐C_3_N_4_ was required to open its coordination sphere to drive exothermic subreactions. Absorption of hydrated potassium phenylboronate acid pinacol ester led to a configuration in which the isolated Pd decreased its coordination to the lattice, while the subsequent displacement of Br^–^ slightly recovered the coordination number. Notably, the adaptive coordination environment originating from the lattice N in g‐C_3_N_4_ plays a vital role in accommodating continuously variable charges over Pd along the reaction coordinate, giving rise to high stability. The subsequent transmetallation step was considered to be the rate‐determining step in the cross‐coupling reaction. Similar to the case of Pd(PPh_3_)_4_ and isolated Pd atoms in vacuum, two terms occurred on Pd‐ECN, the Pd^0^/Pd^2+^ cycle and the alternative Pd^2+^/Pd^4+^ cycle, which showed a high reaction profile. The Pd atoms were then restored to the initial coordination state after the final exothermic formation of the C─C bond and the endothermic elimination of the product. The overall pathway of Pd‐ECN was identified to closely follow the molecular mechanism, with only a few differences. This is rationally attributed to the fact that g‐C_3_N_4_ scaffolds to a certain extent mimicked the beneficial functions of the organic ligands in the metal complex and simultaneously avoided the possibility of catalyst deactivation due to the inevitable lability. Consequently, Pd‐ECN demonstrated excellent catalytic performance and robust stability, surpassing the state‐of‐the‐art homogeneous systems for Suzuki coupling. Furthermore, Han et al. incorporated single Cu atoms into the framework of g‐C_3_N_4_ through a facile preorganization strategy.^[^ ^381^ ^]^ Theoretical analysis shows that he presence of single Cu atoms within g‐C_3_N_4_ substrates enhances the activity of surface Pd nanoparticles by improving proton collection capacity and promoting carrier transfer, thereby improving the photocatalytic performance of the Suzuki cross‐coupling reaction.

#### 4.3.3 Alkene Epoxidation

Epoxides constitute valuable intermediates in the fine chemical industry, tremendously stimulating intensive studies of critical alkene epoxidation reactions.^[^ ^384^, ^385^, ^386^, ^387^ ^]^ To achieve these transformations, broad use of oxidants or co‐reagents is usually required to facilitate electrophilic addition.^[^ ^388^ ^]^ Safety and cost concerns have led to alternative schemes, most notably, catalytic oxygen transfer from molecular oxygen to alkenes to form epoxides without the utilization of reducing agents or radical initiators.^[^ ^389^, ^390^ ^]^

**Figure 22:**
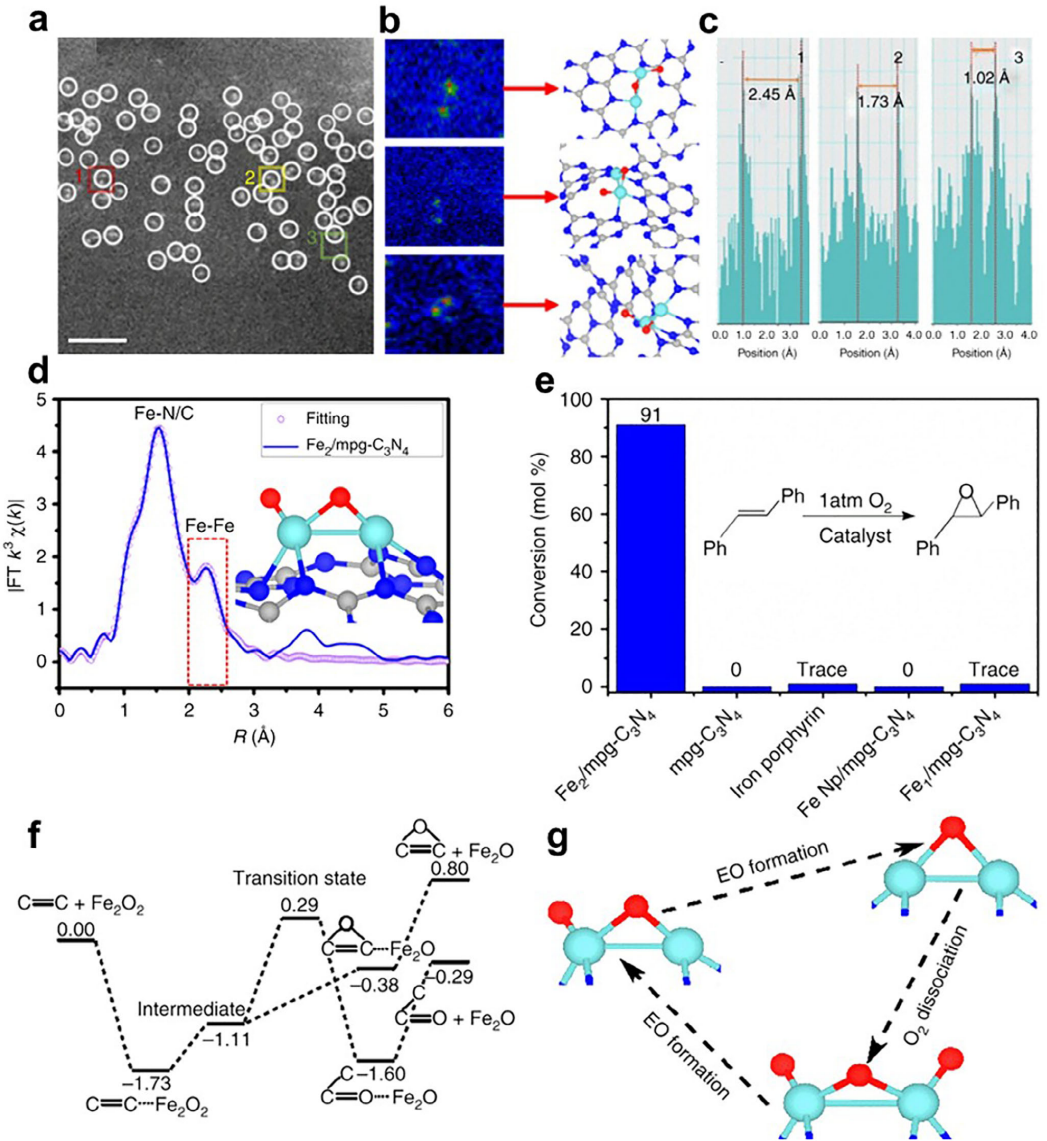
a) Typical AC HAADF‐STEM image of the Fe_2_/mpg‐C_3_N_4_ samples. The scale bar is 1 nm. b, c) Intensity profiles analyzed in the areas of 1, 2, and 3 in (a), and (d) Corresponding fits of the EXAFS spectrum of Fe_2_/mpg‐C_3_N_4_ at R space. e) Histogram of the conversion performances of Fe_2_/mpg‐C_3_N_4_, mpg‐C_3_N_4_, Iron porphyrin, Fe NP/mpg‐C_3_N_4_, and Fe_1_/mpg‐C_3_N_4_ catalysts over epoxidation of trans‐stilbene. f) Energy profile for the epoxidation of trans‐stilbene at the Fe_2_O_2_ site, the unit is eV. g) Consumption and regeneration of the active one coordinated oxygen species, respectively. Reproduced with permission.^[^ ^142^ ^]^ Copyright 2018, Springer Nature.

To date, several homogeneous catalysts, such as iron‐and ruthenium‐substituted polyoxometalates, have reached this rewarding goal, whereas heterogeneous catalysts have rarely been reported.^[^ ^391^, ^392^ ^]^ Li and coworkers^[^ ^142^ ^]^ demonstrated a “precursor‐preselected” wet‐chemistry approach to fabricate Fe_2_ clusters on mesoporous g‐C_3_N_4_ supports. An organic Fe_2_ molecule served as the metal source to coordinate with mpg‐C_3_N_4_, followed by pyrolysis to stabilize the clusters and remove ligands. The HAADF‐STEM image in **Figure** **22**a reveals a clear homogeneous distribution of isolated metallic diatoms with no obvious Fe particles, giving the first indicating the formation of diatomic Fe_2_ clusters. Importantly, depending on the orientation, the detailed features of the Fe_2_ clusters were identified to be diverse, as illustrated by the intensity profiles in Figure 22b. The projected Fe–Fe distance between two adjacent bright dots differed from 1.02 to 2.45 Å, consistent with the bond length of the Fe_2_ dimer (Figure 22c). Following FT‐EXAFS in the R space, two distinct signals are assigned to the Fe–N/O and Fe–Fe coordination paths, respectively, confirming the incorporation of Fe_2_ clusters on mpg‐C_3_N_4_. The local structure of Fe_2_/mpg‐C_3_N_4_ was simulated and is shown in the inset of Figure 22d, where two of the Fe atoms in the Fe_2_ cluster are both anchored by nitrogen atoms in mpg‐C_3_N_4_ and slightly oxidized, linked to oxygen atoms. Typical *trans‐*stilbene epoxidation reactions were employed to evaluate the catalytic properties, and a conversion of 91% and selectivity of 93% were achieved on Fe_2_/mpg‐C_3_N_4_ using O_2_ as the oxidant without any additives (Figure 22e). This excellent performance was further explored using DFT calculations. Interestingly, the isolated Fe site with a one‐coordinated oxygen atom was recognized as the real active center, managing the absorption, epoxidation, and elimination processes sequentially and controlling the activity and selectivity. Upon coordinated oxygen consumption, two more such active centers can be generated through O_2_ dissociation at the other Fe sites. The overall process is schematically presented in Figure 22f,g, and the active oxygen species were the key point during the alkene epoxidation on Fe_2_/mpg‐C_3_N_4_.

## 5 Conclusion and Prospects

The development of efficient, robust, and economical SACs plays an important role in advancing catalysis science and understanding catalytic mechanisms, as well as structure‐property relationships. SACs@g‐C_3_N_4_ represents a unique class of single‐site metal catalysts that combines the merits of both SACs and g‐C_3_N_4_ components and broadens their applications outside of conventional catalysis. In this review, we first summarize the recent progress made in the synthetic methodologies for preparing SACs@g‐C_3_N_4_, which can be classified into three categories for detailed discussion: ALD, impregnation‐reduction synthesis, and pyrolysis. Successful stabilization of SACs on g‐C_3_N_4_ should consider the following aspects: i) suitable metal sources and g‐C_3_N_4_ (precursors) based on the fabrication strategies; ii) a moderate anchoring and stabilization approach to effectively achieve atomic dispersion of SACs against migration and aggregation on g‐C_3_N_4_ substrates; iii) optional secondary treatment to confirm the exclusive single‐site metal catalysts in the final products. There is no doubt that synthetic methods are of great importance in accelerating SACs@g‐C_3_N_4_ studies, from fundamentals to applications. However, during vigorous development, challenges, and opportunities remain.

The SACs@g‐C_3_N_4_ offer significant advantages as a promising material in terms of techno‐economic feasibility and cost‐effectiveness. Compared to conventional support materials like carbon nanotubes or reduced graphene oxide, the precursors of g‐C_3_N_4_‐based materials are exceptionally low‐cost. The synthesis typically involves simple thermal polymerization, an easily scalable and energy‐efficient process that doesn't require sophisticated equipment or hazardous reagents. This translates to a significantly reduced manufacturing cost, a critical factor for large‐scale deployment. In terms of cost‐effectiveness, the high atomic utilization of expensive noble metals (e.g., Pt, Ru) or the substitution with earth‐abundant alternatives (Fe, Co, Ni) anchored on g‐C_3_N_4_ dramatically lowers the catalyst's cost per active site. The robust M–N_x_ coordination ensures excellent stability, reducing the frequency of catalyst replacement in processes like water electrolysis, CO_2_ reduction, or organic hydrogenation. While the initial catalytic activity in some applications may still trail behind premium benchmarks, the outstanding combination of low capital expenditure, minimal raw material cost, and operational durability positions SACs@g‐C_3_N_4_ as a highly economically viable platform for the future of green chemistry and energy technologies.

A major challenge is to increase the metal loading in the SACs@g‐C_3_N_4_ system. The stabilization of SACs@g‐C_3_N_4_ is essentially a balancing exercise between the bonding strengths of metal‐metal and metal‐g‐C_3_N_4_. All the synthetic strategies work on strengthening the critical coordinating steps of metal species with the nitrogen atoms on g‐C_3_N_4_, simultaneously restraining the competition from the formation of metal‐metal bonds. In this context, a sparse dispersion of metal precursors is typically used to overcome the tendency of the metal coalitions and aggregation into particles during precursor reduction. In most studies, the metal loading is generally lower than 5 wt.%, leading to the production of a low number of active sites and insufficient performance unfit for practical applications. However, in theory, one tri‐s‐triazine can coordinate one metal atom, which means that two g‐C_3_N_4_ moieties can coordinate one metal atom, and the maximum metal loading can reach ≈20 wt.% for Fe, Co, Ni, Cu, Zn, and even far higher for noble metals. Thus, modulating the existing synthetic methods and developing novel strategies for obtaining high metal loading with exclusive single‐atomic dispersion is urgently needed. In addition to the high metal loading, another tough goal is to optimize the percentage of surface‐exposed SACs on g‐C_3_N_4_, which is key to ensuring that the isolated metal atoms function as the active centers participating in the catalytic reaction to a large extent. Porous nanostructures can simultaneously accommodate numerous active sites and provide a sufficient number of reaction interfaces. Hence, a novel synthesis process for the construction of porous nanostructured SACs@g‐C_3_N_4_ is highly desirable.

The next priority is to precisely control and adjust the coordination number and local structures of the metal active centers in SACs@g‐C_3_N_4_. A well‐defined structure and even coordination environment are always of critical importance in the fabrication of SACs owing to the precise information on the identification of catalytically active sites and investigation of the catalytic mechanism at the atomic level. g‐C_3_N_4_ with period‐ordered cavity structures is considered an ideal substrate for stabilizing isolated SACs. Most importantly, theoretical prediction and experimental studies revealed that g‐C_3_N_4_ can optimize the electronic structures and catalytic properties of the SACs. Many pioneering efforts are required to elaborate on the coordination number and boosting mechanism of SACs on g‐C_3_N_4_. However, the fine structure of the metal coordination in g‐C_3_N_4_ is still unclear, and the coordination ability of g‐C_3_N_4_ to different metals, as well as their chemical interactions, remains to be further explored. Defect engineering or heteroatom doping of g‐C_3_N_4_ can potentially enhance the interactions between isolated metal atoms and the g‐C_3_N_4_ substrate, thereby improving the activity and stability of SACs@g‐C_3_N_4_. The implementation of these processes makes it possible to tune the coordination environment of the SACs and regulate the reactivity of the metal active sites. Thus far, it has rarely been reported owing to the difficulty in controlling the structures of SACs on deficient or doped g‐C_3_N_4_ and identifying the location of the active site. It is of great significance to adjust, modify, and analyze the structure of the entire SACs@g‐C_3_N_4_ and correlate the exact structural configuration with the catalytic properties, which will provide guidance for designing efficient strategies for optimizing the intrinsic activity of SACs@g‐C_3_N_4_ in specific catalytic reactions.

In the second half of this review, we highlight the achievements of SACs@g‐C_3_N_4_ in a variety of emerging applications, including electrocatalysis, photocatalysis, and organic chemical transformations. For each field of application, we discuss the specific roles of SACs and g‐C_3_N_4_, elucidate their unique structure‐dependent catalytic performance, and reveal the underlying mechanism through concrete examples. In general, SACs@g‐C_3_N_4_ maximizes the utilization efficiency of metal resources, thereby reducing catalyst costs and promoting the economic feasibility of catalytic systems. The fully exposed active sites and distinct electronic structures endow significantly enhanced catalytic performance, and the structural simplicity and homogeneity offer a vast opportunity for studying the catalytic process and mechanism at the atomic scale. Based on a comprehensive investigation of SACs@g‐C_3_N_4_, future development opportunities for SACs@g‐C_3_N_4_ are specified below.

- In electrocatalysis, SACs@g‐C_3_N_4_ is known for its high activity, high selectivity, and durable stability. However, g‐C_3_N_4_ exhibits poor electrical conductivity owing to its intrinsic porous structure. To address this issue, carbon materials, including graphene, carbon nanotubes, and activated carbons were incorporated into the SACs@g‐C_3_N_4_ system as conductive components. The integration mode between SACs@g‐C_3_N_4_ and the carbons (covalent bonding, non‐covalent bonding, π–π interactions, etc) is extremely crucial to the electrochemical properties, considering the number of active sites, the bulk and charge transfer resistances, and the stability of the overall catalyst under harsh electrochemical conditions. Therefore, there is an urgent need to balance the conflicting factors. The electrochemical process is usually complicated and involves multielectron transfer pathways. SACs@g‐C_3_N_4_ cannot simultaneously drive the adsorption, activation, and catalysis of the reaction molecules because of the simple isolated metal active sites, which decrease the catalytic efficiency and limit the applications for several electrocatalytic reactions, such as methanol oxidation. An alternative approach is to fabricate SACs@g‐C_3_N_4_ catalysts with more than one type of metal in every active center. Considering the promoted kinetics and probable synergistic effects, the atomically dispersed catalysts with more than one metal are highly desired, which also holds the promise to offer new catalytic pathways, reduce energy barriers, and enhance catalytic efficiency. Although the catalytic mechanism for some electrochemical conversion processes has been investigated by DFT calculations, it is still difficult to determine a clear reaction pathway and elaborate on the mechanistic details of the catalyst surface. This is due to the lack of comprehensive structural knowledge of SACs@g‐C_3_N_4_ and experimental insight into metal‐reactant interactions under practical conditions. The continued efforts should focus on a deep understanding of the structure‐property relationship and catalytic mechanism of the reactions via a combination of experiments and theory, which will shed light on the optimization of SACs@g‐C_3_N_4_ and provide a research basis for its rational design toward highly efficient electrochemical energy conversion processes.
- For photocatalysis, the SACs@g‐C_3_N_4_ is expected to be an extremely promising material for various photocatalytic applications. As a fascinating conjugated semiconductor, g‐C_3_N_4_ meets the stringent requirements of photocatalysts with good light‐harvesting properties, stability under operational conditions, and suitable energy band positions. The factors hindering its practical use mainly consist of poor electron‐hole separation efficiency, short electron diffusion length, and deficient hole transfer from the g‐C_3_N_4_ surface to the solution. An efficient and effective strategy is to incorporate a metal co‐catalyst, and SACs serve as the ideal choice because of the elimination of the Schottky barrier existing in the traditional metal‐semiconductor junction, which benefits from the absence of an interface. Considerable efforts have been made in the past decade; however, some controversies regarding the photocatalytic mechanism of SACs@g‐C_3_N_4_ remain unresolved. First, the isolated metal atoms on g‐C_3_N_4_ are charged because of the coordinated bonds of M–N_x_, and upon irradiation, the variation in the chemical state of the metal centers, accompanied by its impact on the absorption and activation behaviors toward reacting molecules, is lacking, which is vital to optimize the photocatalytic performance and unveil the corresponding mechanism. Therefore, the dynamic process of the SACs@g‐C_3_N_4_ reaction system must be explored through in situ*/operando* characterization techniques and theoretical analysis. Second, the effects of the interaction between SACs and g‐C_3_N_4_ on the charge separation and transfer processes are necessary for a comprehensive view of the reaction kinetics. Finally, to achieve substantial progress in the utilization of SACs@g‐C_3_N_4_ materials in photoelectric devices, several important issues should be resolved, especially for their efficient deposition on a conductive substrate. In comparison with the well‐established synthetic strategies for SACs@g‐C_3_N_4_ powders, the fabrication of SACs@g‐C_3_N_4_ films is more difficult and controls their morphology, nanostructure, optical, and catalytic properties. The study in this arena is of great practical significance, but still in its infancy, and intensive attention and effort should be devoted.
- For other unconventional heterogeneous catalysis, relevant investigations involving SACs@g‐C_3_N_4_ materials are still in the initial stages. To date, only a few reactions have demonstrated improvements with the use of SACs@g‐C_3_N_4_, including hydrogenation reactions, Suzuki coupling reactions, and alkene epoxidation reactions. However, considering the current revolution in the modern chemical industry, next‐generation catalysts are urgently needed for industrial catalysis processes to achieve the goals of adapting diverse renewable feedstocks and reducing their impact on the environment. SACs@g‐C_3_N_4_ represents the most potential candidate by virtue of the lowest consumption of metal resources together with high activity, stability, and selectivity. Hence, in the next few decades, it is anticipated that there will be a large number of important research results on SACs@g‐C_3_N_4_ for applications related to heterogeneous catalysis. Moreover, novel concepts in the single‐atomic catalysis system also raise the possibility of exploring heterogeneous reactions to replace traditional homogeneous reactions, which is pivotal for promoting sustainability.
- For advanced analysis tools. In situ*/*operando characterizations allow researchers to dynamically track the evolution of single‐atom active sites, identify the true nature of catalytic centers by capturing transient reaction intermediates, and directly correlate the electronic structure and coordination environment of metal atoms with their catalytic activity, thereby offering an understanding of reaction mechanisms and the dynamic metal‐support interactions. However, the application of in situ/operando studies to SACs@g‐C_3_N_4_ under dynamic catalytic conditions faces significant challenges, primarily due to the difficulty in detecting single‐atom sites with sufficient sensitivity and spatial resolution while differentiating active centers from spectator species. The dynamic nature of catalysis further complicates the capture of transient intermediates and real‐time structural evolution, all amidst interfering signals from the complex reaction environment and potential beam‐induced damage. Overcoming these issues requires the sophisticated integration of complementary characterization techniques with high temporal resolution and theoretical modeling to clearly resolve the active sites and mechanistic pathways, which is crucial for advancing the rational design of these promising catalytic systems.

With this and all the great progress during the last 2–3 years in mind, we have good reasons to believe that SACs@g‐C_3_N_4_ remains the most active new frontier in the field of modern chemistry and materials. It is also very exciting to note that an increasing number of scientific researchers, especially young ones, are energetically engaged; thus, we firmly believe that the efforts devoted to the study of SACs@g‐C_3_N_4_ will make it possible to overcome the current difficulties and limitations and pay off for society in the near future.

## Conflict of Interest

The authors declare no conflict of interest.
